# Supplementary material for: Cholesterol Metabolism Is a Druggable Axis that Independently Regulates Tau and Amyloid-β in iPSC-Derived Alzheimer’s Disease Neurons
Source: Cell Stem Cell. 2019 Mar 7;24(3):363–375.e9. doi: 10.1016/j.stem.2018.12.013 (PMC6414424; doi:10.1016/j.stem.2018.12.013)
Supplement: Document S1. Figures S1–S6 and Tables S2, S5, and S6 [file mmc1.pdf]

Cell Stem Cell, Volume *24*

Supplemental Information

Cholesterol Metabolism Is a Druggable Axis that Independently Regulates Tau and Amyloid-β in iPSC-Derived Alzheimer's Disease Neurons

Rik van der Kant, Vanessa F. Langness, Cheryl M. Herrera, Daniel A. Williams, Lauren K. Fong, Yves Leestemaker, Evelyne Steenvoorden, Kevin D. Rynearson, Jos F. Brouwers, J. Bernd Helms, Huib Ovaa, Martin Giera, Steven L. Wagner, Anne G. Bang, and Lawrence S.B. Goldstein

Supplemental Figure 1. Characterization of neuronal cultures and statin effects on pTau and viability, Related to Figure 1.

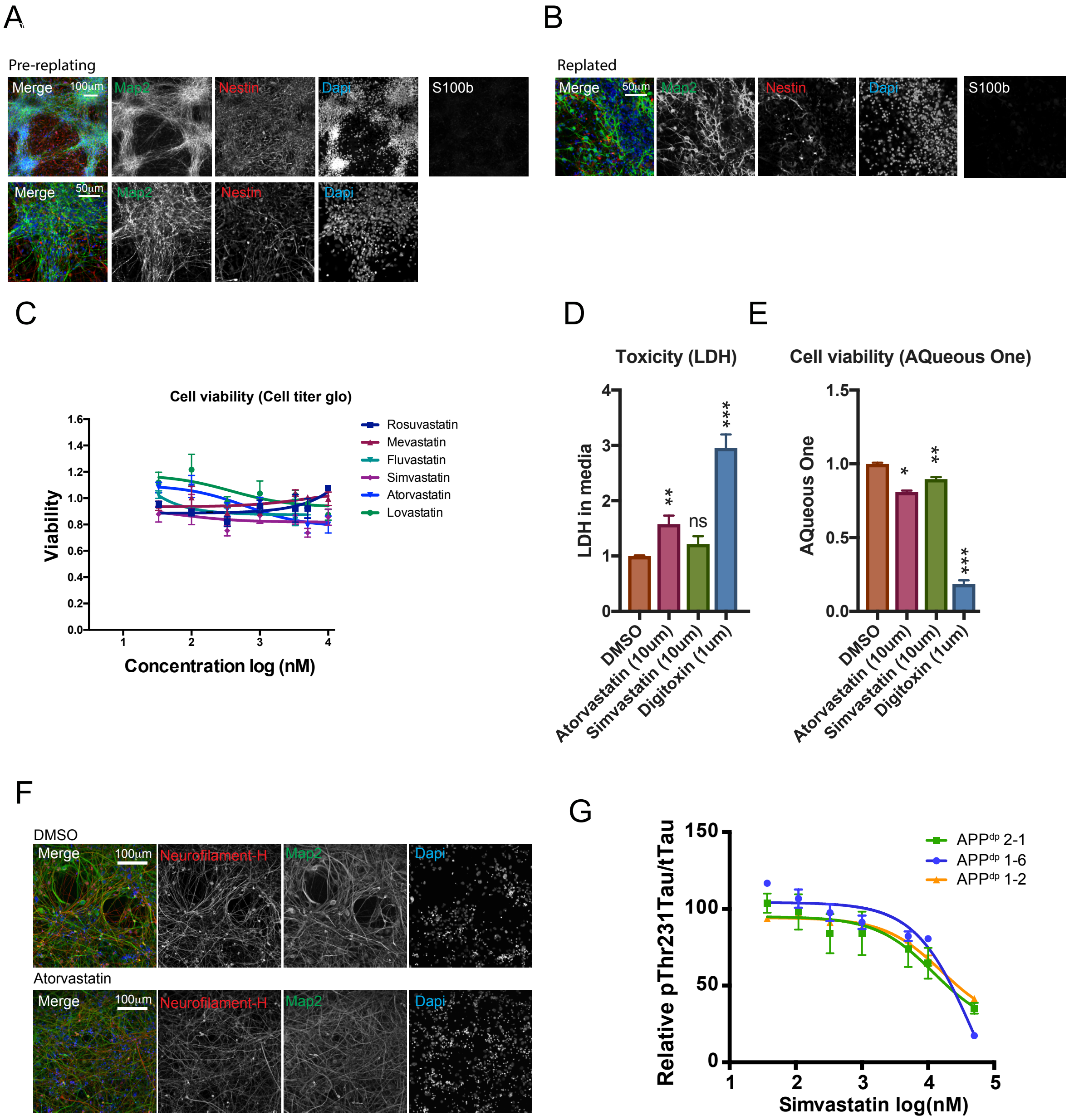

(**A-B**) Three week differentiated neuronal cultures (**A**) and differentiated neurons at point of screening (three weeks of differentiation, replating and two additional weeks of maturation) Scale bar represents 100µm. (**B**). Cultures were fixed and stained with antibodies for mature neurons (MAP2), neuronal progenitor cells (Nestin) and astrocytes (S100b). In keeping with previous observations at these time points the majority of cells are MAP2 neurons with a smaller group of nestin-positive neuronal precursor cells and absence/very low abundance of

astrocytic cells (Yuan et al., 2011). Scale bar represents 100µm. (**C**) Dosage effects of statins (five day treatment) on neuronal viability as measured by CeLLTiter-Glo luminescent cell viability assay. (**D-E**) Effects of a single dose of statins and a known toxic agent (Digitoxin) (five day treatment) on toxicity (**D**) determined by lactate dehydrogenase (LDH) released into the media (mean±SEM, n≥3) and (**E**) cell viability (mean±SEM, n≤3) determined by AQueous one cell viability assay (mean±SEM, n≥3). **(F)** Neuronal cultures (differentiated for three weeks, replated, matured for two more weeks) were treated with atorvastatin or DMSO for five days and stained for MAP2 and Neurofilament-H. Scale bar represents 100µm. **(G)** Dosage effects of simvastatin on pThr231Tau/tTau ratio in an additional FAD line from patient 1 (APP^{dp}1-2) and in an independent FAD APP^{dp} patient line (APP^{dp}2-1) (mean±SEM, n≥3). Dosage effects are compared to the effect of simvastatin on APP^{dp}1-6 from figure 1d. *** p<0.001, **p<0.01, *p<0.05.

Supplemental Figure 2. Effect of cholesterol-metabolism targeting compounds on neuronal viability and cholesterol metabolism, Related to Figure 2.

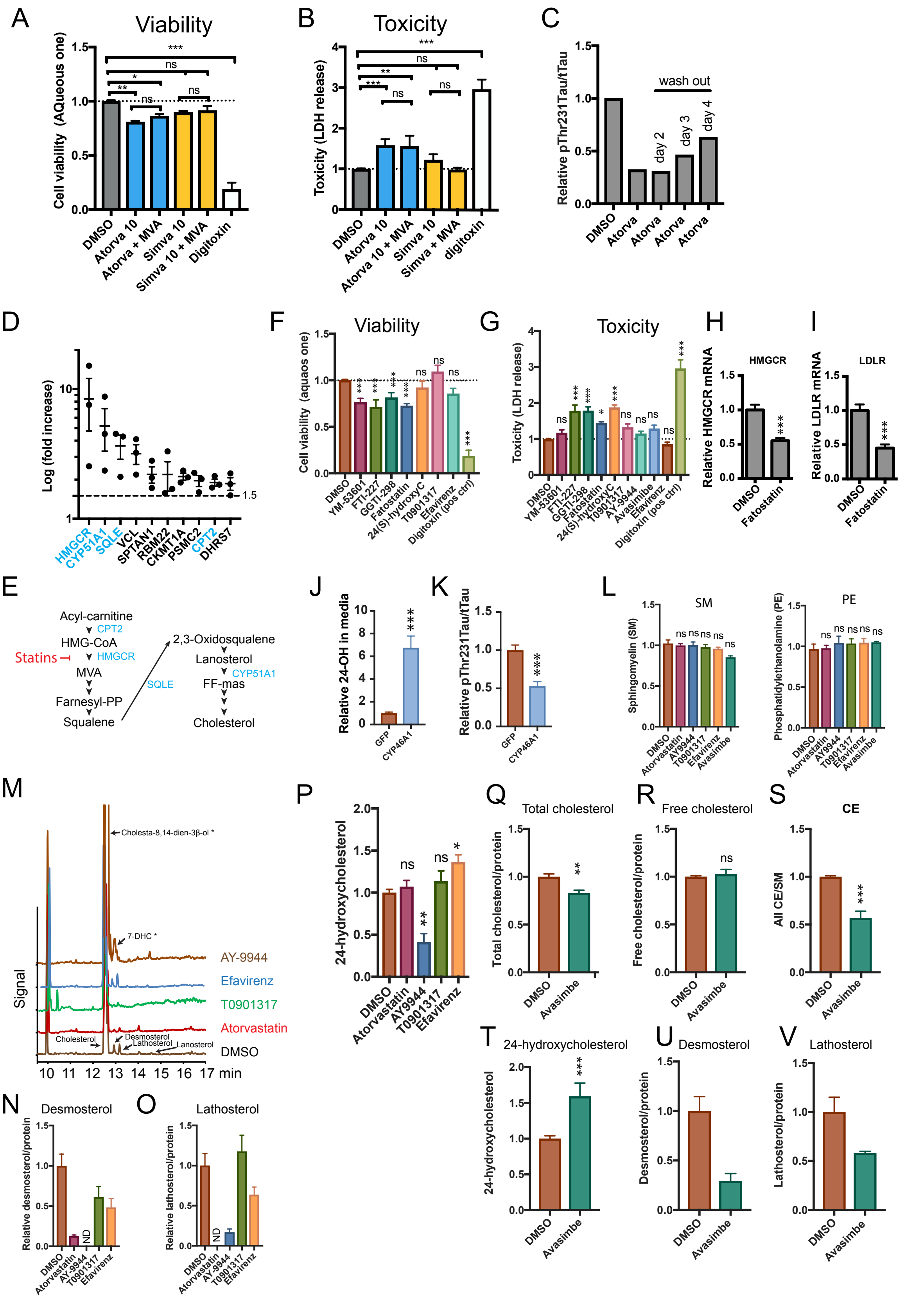

Effects of indicated compounds at same concentrations as in Figure 2 and a known toxic agent (Digitoxin) (five day treatment) on cell viability determined by (**A**) AQueous one cell viability assay (mean±SEM, n≥3). (**B**) lactate dehydrogenase (LDH) released into the media (mean±SEM, n≥3). (**C**) Effect of atorvastatin and atorvastatin washout on pThr231Tau/tTau ratio in APP^{dp}1-6. Neurons were treated for 5 days with DMSO or Atorvastatin and directly harvested, or after 5 days atorvastatin was washed-out and cells were harvested 2-4 days after wash out. (**D**) Purified neurons (NDC line B11 and B10) were treated for three days with atorvastatin or DMSO and harvested for quantitative mass spectrometry. In total three runs from three independent experiments were performed (two different experiments with B11 and one experiment with B10). All proteins that were increased >1.5 fold in all three runs were selected as hits and plotted by their effect on fold change (mean±sem). For complete dataset see Table S4. Proteins with a role in cholesterol synthesis are indicated in blue. HMGCR, 3-Hydroxy-3-Methylglutaryl-CoA Reductase; CYP51A1, Cytochrome P450 Family 51 Subfamily A Member 1 aka Lanosterol 14α-demethylase; SQLE, Squalene Epoxidase; VCL, Vinculin; SPTAN1, spectrin alpha, non-erythrocytic 1; RBM22, RNA Binding Motif Protein 22; CKMT1A, Creatine Kinase, Mitochondrial 1A; PSMC2, 26S proteasome regulatory subunit 7; CPT2, carnitine palmitoyltransferase 2; DHRS7, Dehydrogenase/Reductase 7. **(E)** Schematic depicting the identified hits in (D) with a role in cholesterol synthesis. **(F)** Cell viability (AQueous one) and **(G)** compound toxicity (LDH release) of conditions in Figure 2c. Digitoxin was used as a positive control for cell death (mean±SEM, n≥3). **(H-I)** APP^{dp}1-6 Neurons were treated for 5 days with 20µM fatostatin and mRNA levels of SREBP-target genes (3-hydroxy-3-methylglutaryl-coenzyme A reductase, HMGCR **(H)** and Low-Density Lipoprotein Receptor, LDLR **(I)**) were determined by quantitative real-time polymerase chain reaction (RT-qPCR) and normalized over housekeeping genes (RPLP27) (mean±SEM, n≥12). (**J-K**) Differentiated neurons were transduced with lentivirus carrying CYP46A1 or GFP expression vectors. After

13 days 24-hydroxycholesterol in media (**J**) and neuronal pThr231Tau/tTau ratio was determined (**K**). (**L**) APP^{dp}1-6 neurons were treated with Atorvastatin (10 μM), AY-9944 (10 μM), T0901317 (10μM), Efavirenz (10μM) and Avasimibe (10μM) and lipid analysis was performed to measure the proportion of sphingomyelin (SM) and Phosphatidylethanolamine (PE) (mean±SEM, n≥3). (**M-O**) APP^{dp}1-6 neurons were treated with Atorvastatin (10 μM), AY-9944 (5 μM), T0901317 (10μM) and Efavirenz (10μM) for three days and lipid analysis was performed to measure cholesterol precursors. **(M)** Typical traces from lipid GM-MS showing detected cholesterol precursors in neuronal cultures treated with indicated inhibitors (zoomed-in to show precursor peaks). **(N-O**) Overall precursor levels were low abundant and could be quantified only for desmosterol (**N**) and lathosterol (**O**) (mean±SEM). Efavirenz had previously been shown to increase precursor levels, but in our cultures did not increase (rather modestly decrease) precursor levels. *In the AY-9944 treated neurons 7DHC and Cholesta-8,14-dien-3β-o accumulated as previously reported (Giera et al., 2007). N≥4 for all treatments, expect for desmosterol levels in atorvastatin treated samples, which were below detection in two out of four samples. ND=not detected. (**P**) 24-hydroxycholesterol (five day treatment) levels in media from APP^{dp}1-6 neuronal cultures treated with indicated compounds (mean±SEM, n≥3). (**Q-V**) APP^{dp}1-6 neurons were treated with avasimibe (5μM) and lipid analysis was performed to measure (**Q**) total cholesterol (mean±sem, N≥8), three day treatment, (**R**) free cholesterol (mean±SEM, n≥4), three day treatment (**S**) CE (mean±SEM, n≥8) three day treatment (**T**) 24-hydroxycholesterol in media (mean±SEM, n≥4) (five day treatment) and (**U**) desmosterol and (**V**) lathosterol both (mean±SEM, n≥3) three day treatment. *** $p<0.001$, **$p<0.01$, *$p<0.05$.

Supplemental Figure 3. The effect of CE-reducing drugs on pTau in neurons with different genetic backgrounds, Related to Figure 3.

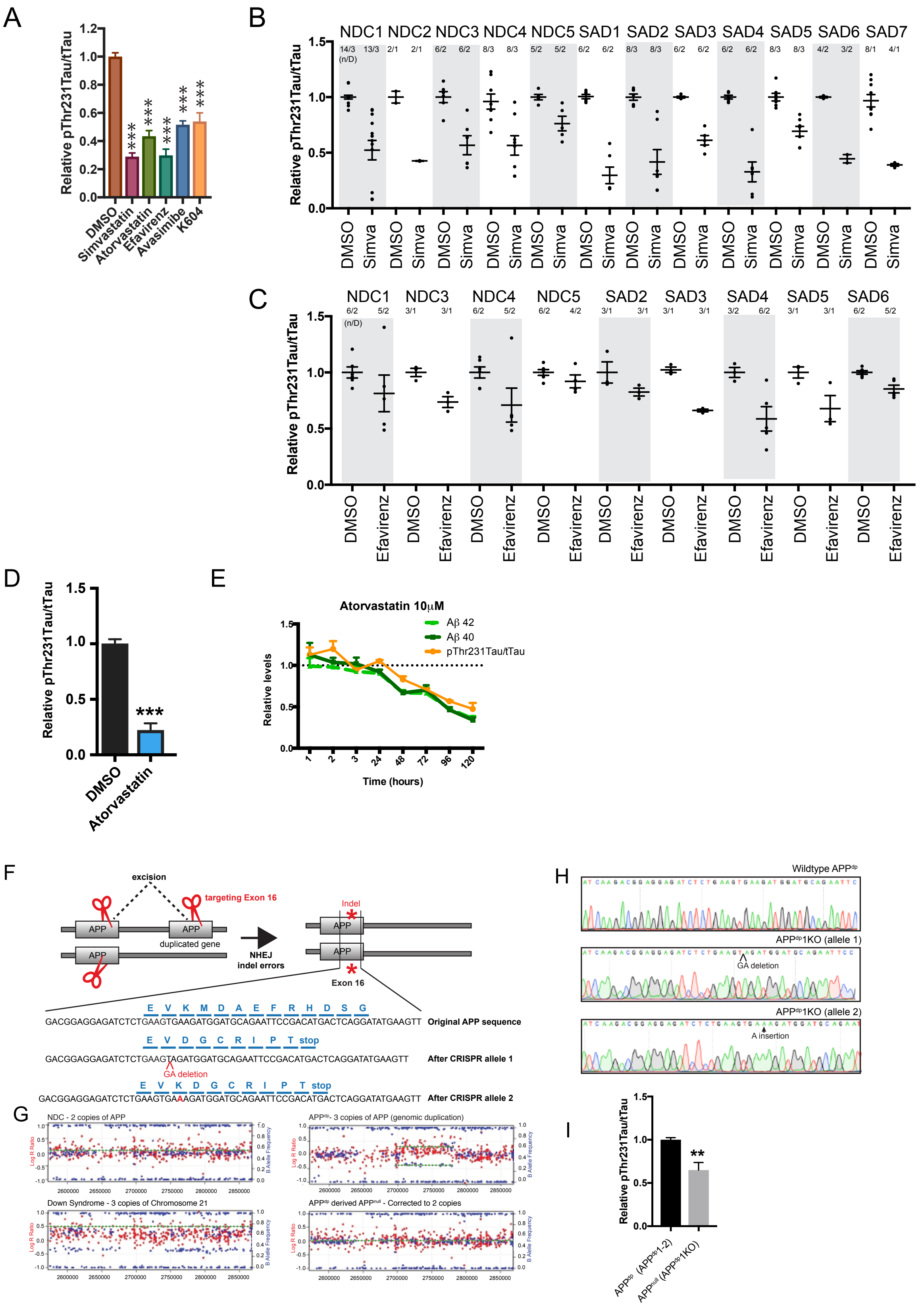

(A) NDC CV4a neurons were treated with indicated compounds; Simvastatin (10 μM), Atorvastatin (10 μM), Efavirenz (25μM), Avasimibe (10μM) and K604 (50μM) pThr231Tau/tTau levels were determined by ELISA (mean±SEM, n≥3). **(B)** Effect of simvastatin (10μm) (**B**) or Efavirenz (10μm) (**C**) treatment on pThr231Tau/tTau in neuronal lines from indicated NDC and SAD subject iPSC-derived neurons (mean±SEM). Same as in main figure 3b-c but here plotted for individual lines. Indicated (n/D) are number of measurements (n) and number of independent differentiations (D). (**D**) Effect of atorvastatin (10μm) treatment on *in vitro* cultured mouse cortical neurons (mean±SEM, n≥3)**.** (**E**) APP^{dp} 1-6 neurons were treated for indicated time points with atorvastatin. Cell lysates and media were analyzed by ELISA for pThr231Tau/ tTau and Aβ levels. (**F**) Schematic overview of the gene-editing strategy to generate an isogenic APP^{null} line in APP^{dp}-genetic background. The original patient has a duplication of APP (three copies of the gene for APP). Cutting by CRISPR/Cas9 at indicated sites (red scissors) causes the excision of the duplication site between cut-sites and correction of copy number. Non-homologues end-joining (NHEJ) insertion/deletion errors (indel) generated during the repair introduce respective premature stop-codons in the two remaining alleles as indicated. (**G**) Copy number analysis (digital karyotyping) of the region with APP duplication for a non-demented control genome, Down syndrome genome (duplication of the entire chromosome 21) and APP^{dp} line before (3 copies of APP) and after ("corrected to 2 copies of APP") gene editing showing copy number correction. **(H)** Sequencing results from TOPO-cloning of the individual alleles verifying a "GA" deletion on one allele and an "A" insertion in the other allele, compared to wildtype sequence (APPdp) (upper box) generating a premature stop. (**I**) pThr231Tau/tTau ratio in isogenic purified APP^{dp} (APP^{dp}1-2) and APP^{null} (APPdp1KO) neurons (five days after sort) (mean±SEM, n≥3). *** $p<0.001$, **$p<0.01$, *$p<0.05$.

Supplemental Figure 4. Characterization of APP-Δcholesterol neurons, Related to Figure 4.

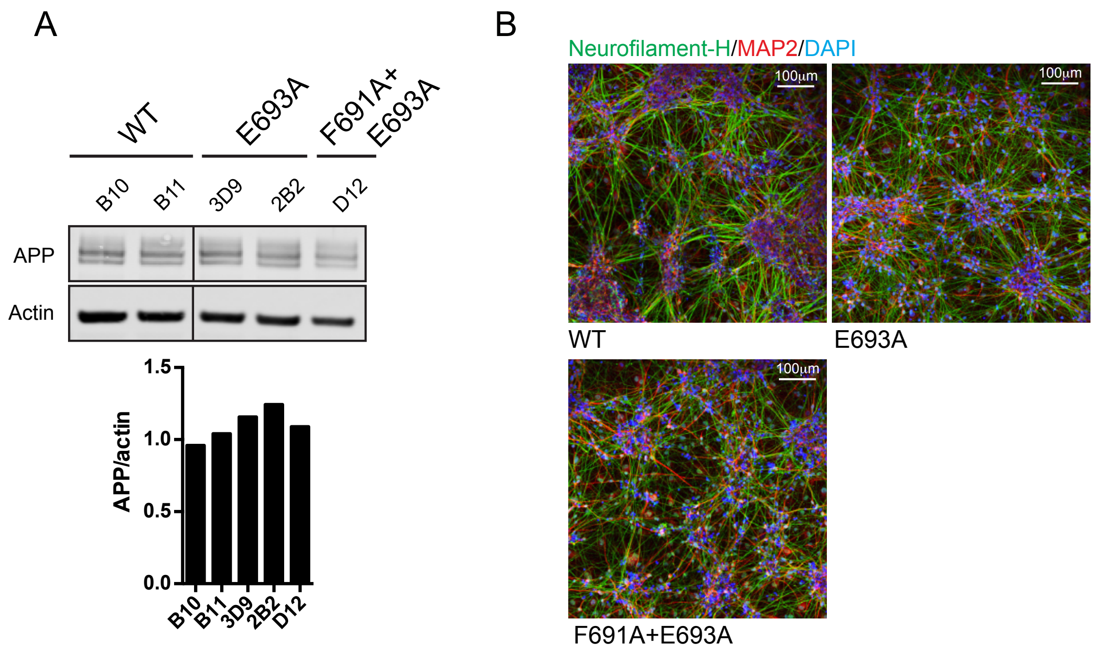

(**A**) Analysis of APP-protein level in control and mutant lines, showing normal expression of full-length APP. Image is a composite of different loading positions on same blot, stich is indicated by vertical line. (**B**) Immunofluorescence staining of wild type and indicated APP-Δcholesterol neurons showing neuronal morphology and polarization. Scale bar represents 100μm.

Supplemental Figure 5. Characterization of drug-treatment effects on neuronal cultures and pTau-mediated turnover by the proteasome, Related to Figure 5.

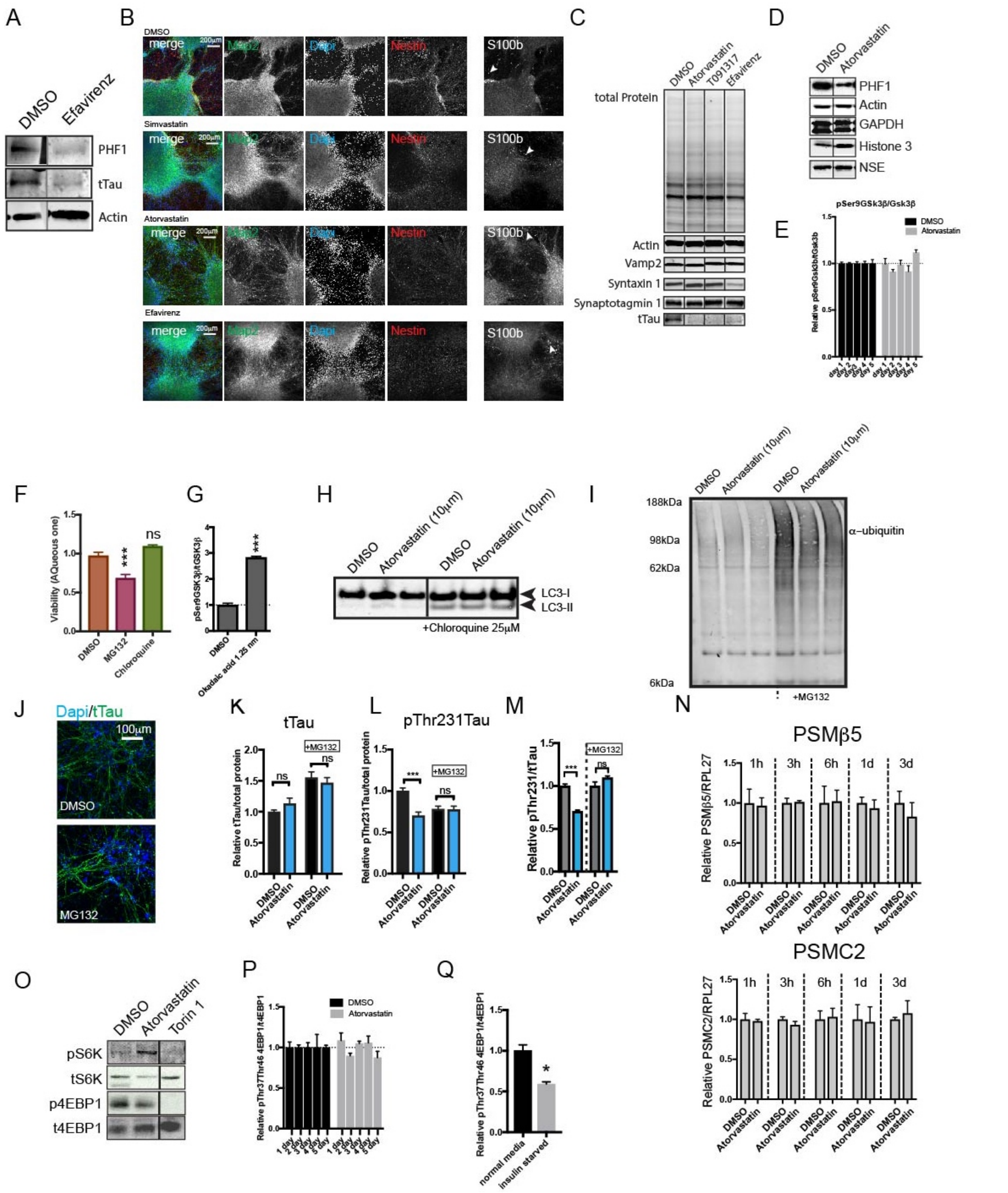

(**A**) The effect of Efavirenz (five day treatment, 10µM, APP[dp]1-6 neurons) on Tau levels and Tau phosphorylation as assessed by western blot (see quantification in main figure 5B). Image

is a composite of different loading positions on same blot, stich is indicated by vertical line. (**B**) Neuronal (MAP2), precursor (Nestin) and astrocyte (S100b) staining of three-week differentiated non-replated neurons (conditions used for western blot) after five day treatment with DMSO, Simvastatin (10µm), Atorvastatin (10µm) or Efavirenz (25µm) in CV4a neurons. High non-specific background staining for S100b was observed in this line, arrows indicate examples of low abundant above-background S100b positive cells. Scale bar represents 200µm. (**C**) Western blot analysis of indicated synaptic proteins after treatment with DMSO, Atorvastatin (10µm), T0901317 (10µm) Efavirenz (25µm) (five day treatment, APP^{dp}1-6 neurons). Image is a composite of different loading positions on same blot, stiches are indicated by vertical lines. (**D**) Western blot analysis of housekeeping genes and neuron-specific enolase (NSE) after treatment with DMSO or Atorvastatin (10µm) (five day treatment, APP^{dp}1-6 neurons). Image is a composite of different loading positions on same blot, stiches are indicated by vertical lines. (**E**) APP^{dp}1-6 neurons were treated for indicated time points with atorvastatin. Cell lysates were analyzed by ELISA for pSer9GSK3b/ tGsk3b (mean±SEM, n≥3). **(F)** APP^{dp}1-6 neurons were treated for 3 days with 25µM chloroquine (inhibitor of lysosomal acidification and autophagosomal degradation) or 5µM MG-132 (proteasomal inhibitor) and cell viability was determined (mean±sem, N≥3). **(G)** APP^{dp}1-6 neurons were treated for 3 days with 1.25nM okadaic acid (phosphatase inhibitor) and pGSK3b/GSK3b levels were determined by ELISA as a measure of phosphatase inhibition (mean±sem, N≥3). **(H-I)** APP^{dp}1-6 neurons were treated for 3 days with 10µM atorvastatin in the presence of 25µM chloroquine (**H**) or 5µM MG-132 (**I**). Lysates were collected and assessed by western blot staining with antibodies against (**H**) microtubule-associated protein 1A/1B-light chain 3 (LC3) to show inhibition of autophagic flux by chloroquine and staining with antibodies for (**I**) ubiquitin to show accumulation of ubiquitinated proteins in MG-132 treated samples. (**J**) APP^{dp}1-6 neurons were treated for 3 days with 5µM MG-132, fixed and stained with a total Tau antibody. (**K**) tTau/total protein levels

and **(L)** pThr231Tau/total protein in APP^{dp} neurons co-treated with atorvastatin (10μm) and the proteasome inhibitor (MG132, 5μM) for 3 days as measured by ELISA. Results are averages from two independent APP^{dp} lines (APP^{dp} 1-6 and 2-1) (mean±SEM, n≥3 per line). (**M**) pThr231Tau/tTau levels in WT neurons co-treated with atorvastatin (10μm) and a proteasome inhibitor (MG132, 5μM) for 3 days as measured by ELISA. Results are averages from two independent WT lines (CV4a and line 151) (mean±sem, N≥3 per line). MG-132 is unstable in solution and was therefore added fresh every day in all experiments. (**N**) APP^{dp}1-6 Neurons were treated for indicated time points with 10μM atorvastatin and mRNA levels of proteasomal subunits PSMβ5 and PSMC2 were determined by quantitative real-time polymerase chain reaction (RT-qPCR) and normalized over housekeeping genes (RPLP27) (mean±SEM, n≥3). (**O**) APP^{dp}1-6 neurons were treated for 5 days with DMSO, 10μM atorvastatin or Torin1 (an mTor inhibitor) and phosphorylation status of mTor target proteins was determined by western blot. Image is a composite of different loading positions on same blot, stiches are indicated by vertical lines. (**P**) APP^{dp}1-6 neurons were treated for indicated time points with DMSO or 10μM atorvastatin and phosphorylation status of the mTor target 4EBP1 was determined by ELISA (mean±SEM, n≥3) (**Q**) Positive control for 4EBP ELISA. APP^{dp}1-6 neurons were treated for three days with insulin containing media or insulin-free media and phosphorylation status of the mTor target 4EBP1 was determined by ELISA (mean±SEM, n≥3). *** p<0.001, **p<0.01, *p<0.05.

Supplemental Figure 6. Astrocytic and neuronal viability in response to statins and efavirenz, Related to Figure 6.

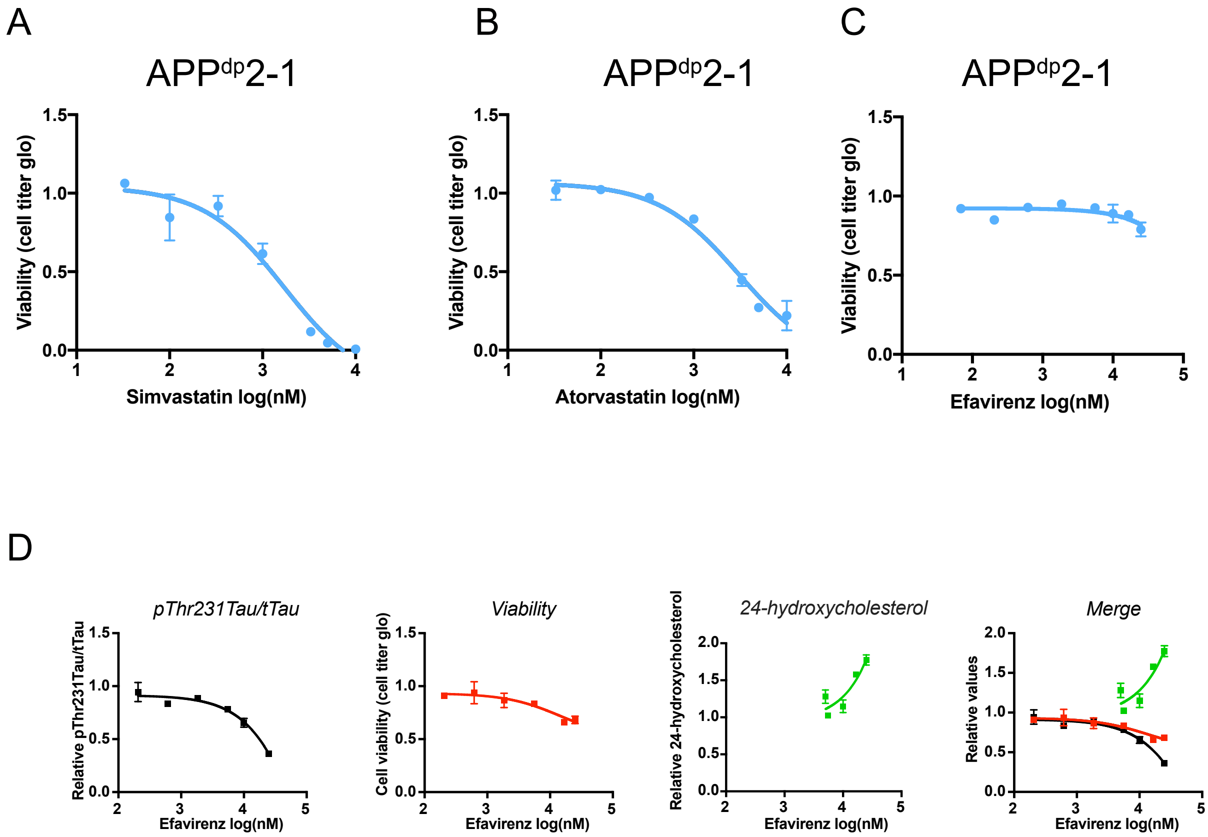

iPSC-derived APP^{dp}2-1astrocytes were treated for three days with increasing concentrations of **(A)** simvastatin, **(B)** atorvastatin and **(C)** efavirenz and viability was determined (cell titer glo). **(D)** APP^{null} (APPdp1KO) neurons were treated for five days with increasing concentrations efavirenz and pThr231Tau/Tau levels, 24-hydroxycholesterol levels and viability was measured (mean±SEM, n≥3).

Supplemental Table 2. Coefficient of variation for screening campaign, Related to Figure 1.

	ptau/total tau ratio			Cell Titer Glo		
plate #	ave	std dev	cv	ave	std dev	cv
1	4.89	1.05	0.21	427566	87006	0.20
2	4.38	0.76	0.17	439798	105410	0.24
3	4.80	0.68	0.14	417468	46284	0.11
4	4.42	0.71	0.16	413676	65711	0.16
5	6.05	0.72	0.12	540691	134415	0.25
6	6.88	0.60	0.09	538648	128503	0.24
7	5.87	1.64	0.28	252201	42936	0.17
8	5.79	1.69	0.29	237674	55155	0.23
9	5.03	1.12	0.22	193892	56904	0.29
10	5.30	1.14	0.21	276671	43889	0.16
11	4.36	1.01	0.23	202913	60936	0.30
12	5.18	1.15	0.22	215490	47718	0.22
13	5.15	1.23	0.24	221407	75762	0.34
14	4.30	0.65	0.15	224197	41101	0.18
15	6.07	1.47	0.24	233372	57994	0.25
16	7.76	0.69	0.09	534782	49171	0.09
17	6.57	1.30	0.20	377836	67072	0.18
18	4.38	1.04	0.24	333269	51268	0.15
19	4.99	1.35	0.27	336305	76970	0.23
20	5.09	0.94	0.19	421618	90686	0.22
			AVERAGE CV			AVERAGECV
			**0.20**			**0.21**

Coefficient of variation (CV) for pThr231Tau/tTau and cell titer glo in DMSO-treated samples for each of the 20 384 well plates used for the screening campaign (primary screen, conformation, dose response experiments)

Supplemental Table 5. Absolute levels of cholesterol, Related to Figure 2.

	free cholesterol ng/ug protein		total cholesterol ng/ug protein	
	mean	stdev	mean	stdev
DMSO	85.9	1.6	119.1	10.1
Atorvastatin	89.5	6.3	99.4	8.3
AY-9944	67.2	2.5	76.6	4.5
T0901317	73.0	6.9	104.2	8.2
Efavirenz	75.1	6.6	88.1	4.5
Avasimibe	88.2	8.4	98.7	9.8

Table depicts the absolute levels of free cholesterol (N=8) and total cholesterol (N=4) over protein corresponding to the relative levels plotted in figure 2d and e.

Supplemental Table 6. Oligonucleotides used in this study, Related to STAR methods.

Oligonucleotides	Source	Identifier
5'tattgcatttagaaattaaaattctttttcttaatttgttttcaaggtgttcttt gcagccgatgtgggttcaaacaaaggtgcaatcattggactcatggtgg gcggtgttgtcatagc3'	This Paper	E693A Repair Oligo
5'ttatattgcatttagaaattaaaattctttttcttaatttgttttcaaggtgttc gctgcagccgatgtgggttcaaacaaaggtgcaatcattggactcatgg tgggcggtgttgtcat3'	This Paper	E693A+F691A Repair Oligo
5'CTTCCTCGAACTGGGGAAGC3'	This Paper	APP Chol mut region forward primer
5'TCACGGTAAGTTGCAATGAATGA3'	This Paper	APP Chol mut region reverse primer
5'CCAACCAGTTGGGCAGAGAA3'	This Paper	APP Chol mut genomic DNA Sequencing Primer
5'CGTGGAATGGCAATTTTAGGTCC3'	This Paper	HMGCR-F (qPCR)
HMGCR-R (qPCR): 5'ATTTCAAGCTGACGTACCCCT3'	This Paper	*HMGCR-R (qPCR)*
LDLR-F (qPCR): 5'GTCTTGGCACTGGAACTCGT3'	This Paper	*LDLR-F (qPCR)*
LDLR-R (qPCR): 5'CTGGAAATTGCGCTGGAC3'	This Paper	LDLR-R (qPCR)
5'GCTACCGGTGAACCAGCG3'	This Paper	PSMB5 FW (qPCR)
5'CAACTATGACTCCATGGCGGA3'	This Paper	PSMB5 RV (qPCR)
5'TGAGAGTGGGCGTGGATAGA3'	This Paper	PSMC2 FW (qPCR)
5'GTACCGGGTGGACCAAAGAG3'	This Paper	PSMC2 RV (qPCR)
5'AAACCGCAGTTTCTGGAAGA3'	This Paper	RPL27-F (qPCR)
5'TGGATATCCCCTTGGACAAA3'	This Paper	RPL27-R (qPCR)
5'gtacaaaaaagcaggcttaaatggactacaaagaccatgacgg3'	This paper	CYP46A1 PCR primer 1
5'GTACAAGAAAGCTGGGTAGCggatccTCAGC AGGGGGGTGGT3'	This paper	CYP46A1 PCR primer 2
