# Supplementary material for: Cholesterol Metabolism Is a Druggable Axis that Independently Regulates Tau and Amyloid-β in iPSC-Derived Alzheimer’s Disease Neurons
Source: Cell Stem Cell. 2019 Mar 7;24(3):363–375.e9. doi: 10.1016/j.stem.2018.12.013 (PMC6414424; doi:10.1016/j.stem.2018.12.013)
Supplement: Document S2. Article plus Supplemental Information [file mmc5.pdf]

Article

Cell Stem Cell

Cholesterol Metabolism Is a Druggable Axis that Independently Regulates Tau and Amyloid-β in iPSC-Derived Alzheimer's Disease Neurons

Graphical Abstract

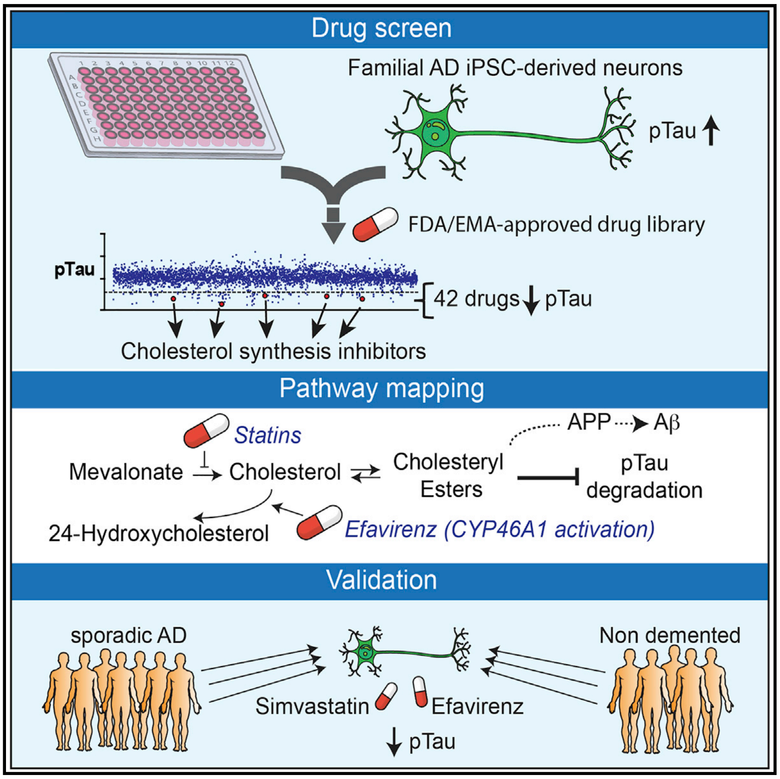

Authors

Rik van der Kant, Vanessa F. Langness, Cheryl M. Herrera, ..., Steven L. Wagner, Anne G. Bang, Lawrence S.B. Goldstein

Correspondence

lgoldstein@ucsd.edu

In Brief

van der Kant et al. performed a repurposing drug screen in iPSC-derived AD neurons and identified compounds that reduce aberrant accumulation of phosphorylated Tau (pTau). Reduction of cholesteryl ester levels or allosteric activation of CYP46A1 by lead compounds enhanced pTau degradation independently of APP and Aβ.

Highlights

- Drug screening identifies inhibitors of aberrant phosphorylated Tau (pTau) accumulation
- Cholesteryl esters (CE) were identified as upstream regulators of pTau proteostasis
- The effects of CE on Tau proteostasis are correlated with, but independent of, APP and Aβ
- Lead compound enhance CE-dependent pTau proteasomal turnover specifically in neurons

van der Kant et al., 2019, Cell Stem Cell *24*, 363–375
March 7, 2019 © 2018 The Authors. Published by Elsevier Inc.
https://doi.org/10.1016/j.stem.2018.12.013

CellPress

Cholesterol Metabolism Is a Druggable Axis that Independently Regulates Tau and Amyloid-β in iPSC-Derived Alzheimer's Disease Neurons

Rik van der Kant,[1,2,10] Vanessa F. Langness,[1,10] Cheryl M. Herrera,[1] Daniel A. Williams,[1,11] Lauren K. Fong,[1] Yves Leestemaker,[3] Evelyne Steenvoorden,[4] Kevin D. Rynearson,[5] Jos F. Brouwers,[6] J. Bernd Helms,[6] Huib Ovaa,[3] Martin Giera,[4] Steven L. Wagner,[5,7] Anne G. Bang,[8] and Lawrence S.B. Goldstein[1,9,12,*]

[1]Department of Cellular and Molecular Medicine, University of California, San Diego, La Jolla, CA 92093, USA
[2]Department of Functional Genomics, Center for Neurogenomics and Cognitive Research, Amsterdam Neuroscience, VU University Amsterdam de Boelelaan 1087, 1081 HV Amsterdam, the Netherlands
[3]Oncode Institute and Department of Cell and Chemical Biology, Leiden University Medical Center, Einthovenweg 20, 2333 ZC Leiden, the Netherlands
[4]Center for Proteomics and Metabolomics, Leiden University Medical Center, Albinusdreef 2, 2333 ZA Leiden, the Netherlands
[5]Department of Neurosciences, University of California, San Diego, La Jolla, CA 92093, USA
[6]Department of Biochemistry and Cell Biology, Faculty of Veterinary Medicine, Utrecht University Yalelaan 2, 3584 CM Utrecht, the Netherlands
[7]Research Biologist, VA San Diego Healthcare System, La Jolla, CA 92161, USA
[8]Conrad Prebys Center for Chemical Genomics, Sanford Burnham Prebys Medical Discovery Institute, 10901 North Torrey Pines Road, La Jolla, CA 92037, USA
[9]Sanford Consortium for Regenerative Medicine, La Jolla, CA 92037, USA
[10]These authors contributed equally
[11]Deceased
[12]Lead Contact
*Correspondence: lgoldstein@ucsd.edu
https://doi.org/10.1016/j.stem.2018.12.013

SUMMARY

Genetic, epidemiologic, and biochemical evidence suggests that predisposition to Alzheimer's disease (AD) may arise from altered cholesterol metabolism, although the molecular pathways that may link cholesterol to AD phenotypes are only partially understood. Here, we perform a phenotypic screen for pTau accumulation in AD-patient iPSC-derived neurons and identify cholesteryl esters (CE), the storage product of excess cholesterol, as upstream regulators of Tau early during AD development. Using isogenic induced pluripotent stem cell (iPSC) lines carrying mutations in the cholesterol-binding domain of APP or APP null alleles, we found that while CE also regulate Aβ secretion, the effects of CE on Tau and Aβ are mediated by independent pathways. Efficacy and toxicity screening in iPSC-derived astrocytes and neurons showed that allosteric activation of CYP46A1 lowers CE specifically in neurons and is well tolerated by astrocytes. These data reveal that CE independently regulate Tau and Aβ and identify a druggable CYP46A1-CE-Tau axis in AD.

INTRODUCTION

Pathological accumulation of phosphorylated Tau (pTau) and accumulation of amyloid-beta (Aβ) fragments are the two major biochemical hallmarks of Alzheimer's disease (AD). Effective strategies to remove Aβ in AD-patient brains have been developed but have not yet shown efficacy to slow cognitive decline in clinical trials. This finding has led to the idea that targeting Tau or combinatorial strategies that target both Tau and Aβ are required to treat AD. While Aβ generation has been studied in much detail, the processes that drive pTau accumulation in AD are poorly defined. Late stage Tau pathology, such as aggregation of accumulated Tau in neurofibrillary tangles (NFT), and subsequent neurodegeneration can be modeled in mice, or non-neuronal human cells, by (over)expression of human mutant Tau. However, Tau mutations do not occur in AD. Instead, in AD, endogenous "wild-type" pTau accumulates downstream of familial AD (FAD) mutations (in APP, PSEN1, and PSEN2 genes) or, in the case of sporadic late-onset AD (SAD), downstream of an unknown combination of genetic and environmental risk factors. How these factors drive early accumulation of pTau in AD is not understood. Recent advances in induced pluripotent stem cell (iPSC) technology (Shi et al., 2017) have made it possible to generate functional human neurons from patients and healthy controls to study early pathophysiological regulation of endogenous Tau. In iPSC-derived neurons from both FAD- and SAD-patients, pTau aberrantly accumulates at early time points (Choi et al., 2014; Israel et al., 2012; Moore et al., 2015; Muratore et al., 2014; Ochalek et al., 2017; Shi et al., 2012). Accumulation of pTau in FAD neurons can be reversed by inhibition of β-secretase, the enzyme that converts APP to β-CTF indicating a direct relationship between APP processing and Tau (Israel et al., 2012; Moore et al., 2015). Interestingly, inhibition of γ-secretase (to prevent generation of Aβ from β-CTF) did not reduce pTau, indicating that the effect of APP processing on pTau in early

This is an open access article under the CC BY license (http://creativecommons.org/licenses/by/4.0/).

AD neurons is not solely mediated by extracellular Aβ (Israel et al., 2012; Moore et al., 2015).

Identification of other cellular pathways that contribute to pTau accumulation early in FAD and SAD neurons is key to understanding Tau pathology in AD. In addition to increased Aβ and pTau levels, CE also accumulate in FAD and SAD. CE are increased in mouse models expressing human (mutant) APP (Chan et al., 2012; Tajima et al., 2013; Yang et al., 2014) and CE, as well as CE-storage organelles (lipid droplets), have been shown to accumulate in the SAD brain (Chan et al., 2012; Foley, 2010; Hamilton et al., 2015; Yang et al., 2014). CE are generated when cholesterol is converted to CE by the ER-resident Acyl-CoA cholesterol acyltransferase (ACAT) through ligation of a long-chain fatty acid to (excess) cholesterol, and CE can be converted back to cholesterol by acidic lipases in the lysosome (Ikonen, 2008; Puglielli et al., 2003). CE enhance the production of Aβ *in vivo* and *in vitro* indicating that CE can contribute to AD pathogenesis (Di Paolo and Kim, 2011; Hutter-Paier et al., 2004; Huttunen et al., 2009; Puglielli et al., 2001, 2003). CE-dependent regulation of Aβ generation is mediated by altered trafficking of APP through the early secretory pathway (Huttunen et al., 2009). Whether CE also affect Tau phosphorylation or Tau proteostasis is unknown, but inhibition of cholesterol esterification by genetic deletion of ACAT1 prevents early stage Tau pathology in Tau mutant mice through unknown mechanisms (Shibuya et al., 2015). A possible way by which CE could affect Tau pathology is through regulation of the ubiquitin-proteasome system (UPS). Cholesterol and cholesterol metabolites extensively interact with the UPS to regulate the ubiquitination and degradation of cholesterol-metabolic enzymes (Sharpe et al., 2014), and the UPS is a major regulator of pTau proteostasis. (Lee et al., 2013). Activity of the UPS is decreased in AD (Keck et al., 2003; Keller et al., 2000), and UPS (re)activation delays Tau aggregation and neurodegeneration *in vitro* and *in vivo* (Han et al., 2014; Lokireddy et al., 2015; Myeku et al., 2016).

Here, we tested a library of >1,600 compounds for their potency to inhibit pTau accumulation in cultured FAD iPSC-derived neurons and find that neuronal CE regulate the proteasome-dependent degradation of pTau. Using neurons derived from multiple AD- and non-demented control (NDC) iPSC lines, as well as isogenic CRISPR/Cas9 gene-edited lines, we demonstrate that the effect of CE on pTau is correlated with, but independent of APP processing and Aβ. Whereas the effect of CE on pTau is mediated by proteasomal upregulation, the effect of CE on Aβ secretion is mediated by a cholesterol-binding domain in APP. We identify a number of strategies to reduce pTau in a CE-dependent manner and find that allosteric activation of CYP46A1 is a neuron-specific CE-lowering strategy particularly well tolerated by human astrocytes. Collectively, our data identify a CYP46A1-CE-Tau axis as an early druggable pathway in AD.

RESULTS

A Drug Screen in iPSC-Derived Human FAD Neurons to Identify Compounds that Reduce pTau Accumulation

pThr231Tau is an early marker of AD pathology that correlates well with cognitive decline (Buerger et al., 2002; Luna-Muñoz et al., 2007). pThr231Tau accumulates in APP duplication (APP^{dp}) iPSC-derived FAD neurons (Israel et al., 2012). To identify compounds that reduce pTau accumulation in these FAD neurons, we screened a collection of 1,684 approved and preclinical drugs for their efficacy to lower neuronal pThr231Tau. For our screen, neural progenitor cells (NPCs; line APP^{dp}1-6) (Israel et al., 2012) were differentiated to neurons (Figures S1A and S1B) for 3 weeks, replated in 384 well plates, and allowed to mature for 2 weeks before treatment with compound at 5 μM for 5 days. The screen was performed in duplicate, and a ratiometric readout of pThr231Tau/total Tau (tTau) level and cell viability was determined (Figure 1A). In the primary screen, 158/1,684 compounds (9.4%) significantly reduced pThr231/tTau by a Z score <-2 in at least one of the duplicates (Figure 1B; Tables S1 and S2) and were selected for confirmation. In a repeat of the primary assay with selected compounds, 96/158 compounds were confirmed to reduce pThr231/tTau by a Z score <-2 in at least one additional replicate (Table S3). Of the 96 confirmed compounds, 42 were clearly non-toxic hits with $Z > -1$ for viability (Figure 1C). Our screen identified six microtubule-interacting compounds that reduced pThr231Tau/tTau (14% of hits) that have previously been shown to regulate pTau in other systems (Dickey et al., 2006; Merrick et al., 1996; Xie et al., 1998). Our hit-list also included four inhibitors of cholesterol synthesis; atorvastatin, simvastatin, fluvastatin, and rosuvastatin. Because cholesterol metabolism has been heavily linked to AD pathogenesis (Di Paolo and Kim, 2011) we selected these compounds for further study. We confirmed that these four statins, as well as two additional statins (lovastatin and mevastatin), reduced pThr231Tau/tTau in a dose-dependent manner with minor effects on cell viability or neuronal number (Figures 1D and S1C–S1F). Simvastatin reduced pThr231Tau in a similar dose-dependent manner in additional lines from the same patient (APP^{dp}1-2) and an independent patient APP^{dp} line (APP^{dp}2-1) (Figure S1G), indicating that the effect of statins is conserved across individual APP^{dp} lines and patients. In addition to pThr231Tau/tTau, atorvastatin also reduced pS396/S404Tau, pS202/T205Tau and levels of a pThr231 phosphorylation-dependent conformational Tau epitope (TG3) as assessed by immunofluorescence (Figure 1e). These data show that in addition to screening for Aβ (Brownjohn et al., 2017; Kondo et al., 2017), iPSC-derived AD neurons can be applied to screen for pTau modulators. In addition, these data show that statins reduce pTau levels across a number of phosphorylation epitopes and across individual FAD (APP^{dp}) patients.

The Effect of Statins on pTau Is Mediated by Cholesteryl Esters

To understand how pTau is regulated by statins, we studied the relationship between the mevalonate-cholesterol synthetic pathway and pTau levels in more detail. Statins inhibit 3-hydroxy-3-methylglutaryl-CoA reductase (HMGCR), an early rate limiting step in the cholesterol synthetic pathway that converts HMG-CoA to mevalonate (MVA) (Figure 2A). As expected, MVA supplementation rescued the effect of atorvastatin treatment on pThr231Tau/tTau indicating that the effect of statins on pTau is specific to their effects on the mevalonate pathway (Figure 2B). While atorvastatin was slightly cytotoxic at higher concentrations (Figures S2A and S2B) toxicity did not explain the effect of atorvastatin on pThr231Tau/tTau, as MVA supplementation completely rescued the effect of atorvastatin on

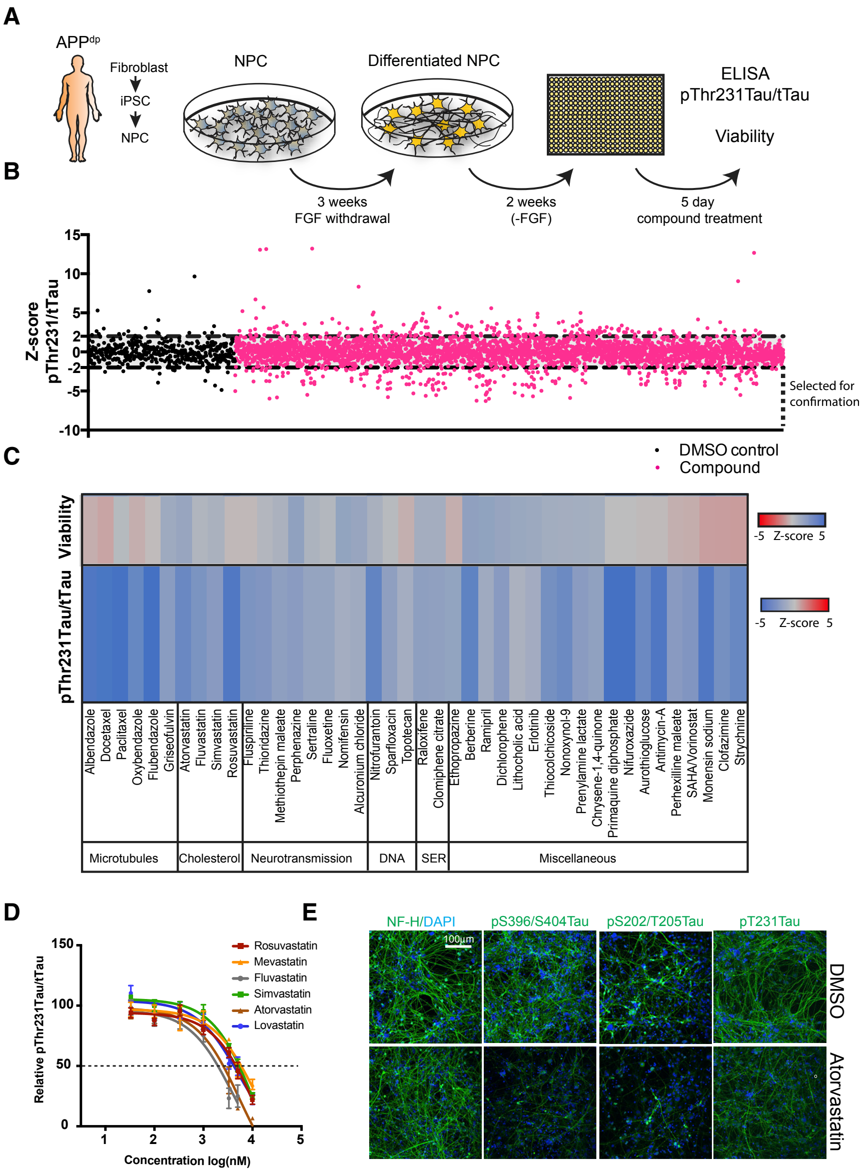

Figure 1. Identification of Compounds that Decrease pTau Levels in FAD iPSC-Derived AD Neurons

(A) Screening strategy overview: APP^{dp}1-6 NPC's were differentiated for 3 weeks, replated into 384 well plates, and after 2 weeks, treated with 5 μM of compound for 5 days; pThr231Tau/tTau ratio and cell viability was measured.

(B) 1,684 compounds (in pink) were screened in duplicate for their effect on pThr231Tau/tTau ratio as expressed by Z score. 158 compounds that decreased pThr231/tTau by $Z \leq -2$ were selected for confirmation. Vehicle alone controls (DMSO) are shown in black.

(C) 42 confirmed non-toxic hits grouped by drug category. SER, selective estrogen reuptake (n = 4).

(D) Dosage effects of different statins on pThr231Tau/tTau ratio in APP^{dp}1-6 (mean ± SEM, n $\geq$ 3).

(E) APP^{dp}1-6 neurons were treated with vehicle (DMSO, upper row) or atorvastatin (10 μM, lower row) for 5 days and fixed and stained with antibodies for indicated antigens. NF-H, neurofilament H, axonal marker. The pThr231Tau antibody used is TG3, which detects a conformational epitope of pThr231Tau. Scale bar, 100 μm.

See also Figure S1.

pThr231Tau/tTau ratio without rescuing the minor effect of atorvastatin on cell viability (Figures S2A and S2B). Removal of atorvastatin after 5 days of treatment allowed recovery of pThr231Tau/Tau indicating reversible dynamic regulation (Figure S2C). Statin-treated neurons elicited a (normal) physiological response to statin treatment exemplified by upregulation of cholesterol-synthetic proteins (Figures S2D and S2E; Table S4). Statins have recently been reported to induce the degradation of mutant p53 by reduction of mevalonate-5-phosphate (MVP) (Parrales et al., 2016). The effects of statins on pTau are not mediated by MVP, as both MVP and its direct downstream metabolite, MVA-5PP, rescued the effect of statin treatment (Figure 2B). More distal of MVA-5PP, the MVA pathway branches into non-sterol isoprenoid (protein prenylation) pathways and a cholesterol synthetic pathway (Figure 2A). Inhibition of the respective non-sterol isoprenoid pathways (by geranyl- or farnesyl-transferase inhibitors GGTI-298 and FTI-227, respectively) did not significantly reduce pThr231Tau/tTau (Figures 2C, S2F, and S2G). In contrast, inhibition of the cholesterol-synthetic arm of the mevalonate pathway using a squalene synthase inhibitor (YM-53601) or Δ7-dehydrocholesterol reductase (DHCR7) inhibitor (AY-9944) did significantly decrease pThr231Tau/tTau (Figures 2C, S2F, and S2G), indicating that pTau is regulated by the cholesterol-synthetic branch of the MVA pathway. Alternative ways to reduce neuronal cholesterol such as inhibition of sterol regulatory element-binding protein (SREBP)-mediated transcriptional activation of cholesterol synthetic genes by fatostatin (Kamisuki et al., 2009) (Figures 2C, S2H, and S2I) or induction of cholesterol export by liver X receptor (LXR) agonists (Figure 2A) T0901317 and 24-hydroxycholesterol (Figures 2C, S2F, and S2G) also reduced pThr231Tau/tTau. Activation of homologous nuclear receptors such as PPARα (by GW50156) and PPARγ (by Rosiglitazone), which regulate fatty acid metabolism but not cholesterol export, did not decrease pThr231Tau/tTau (Figures 2C, S2F, and S2G). Another strategy to reduce the pool of neuronal cholesterol is the activation of cholesterol 24-hydroxylase (CYP46A1), a neuron-specific enzyme that converts cholesterol to 24-hydroxycholesterol (Anderson et al., 2016; Mast et al., 2017b; Moutinho et al., 2016). As expected, activation of CYP46A1 by efavirenz (Figures 2C, S2F, and S2G) or overexpression of CYP46A1 (Figures S2J and S2K) also reduced pThr231Tau/tTau. Together, these data show that mechanistically different cholesterol-lowering drugs all reduce pTau, strongly indicating that pTau levels are

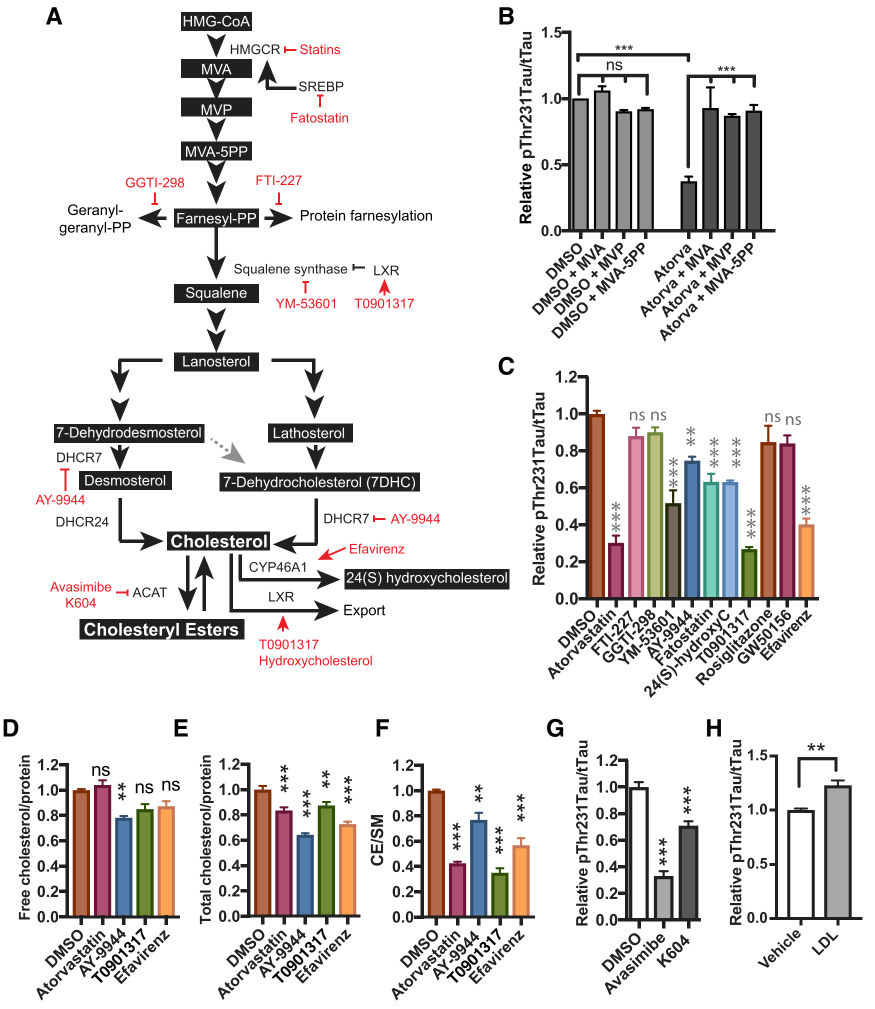

Figure 2. The Effect of Statins on pTau Is Mediated by Cholesteryl Esters

(A) Overview of the mevalonate pathway and inhibitors used in this study.

(B) APP^{dp}1-6 neurons were treated with DMSO or atorvastatin (10 μM) for 5 days. For indicated conditions, mevalonate (MVA, 0.5 mM), mevalonate-5-phosphate (MVP, 0.5 mM), or mevalonate-5-pyrophosphate (MVA-5PP, 1 mM) was added to the media at a single dose at t = 0 (mean ± SEM, n ≥ 3).

(C) APP^{dp}1-6 neurons were treated with inhibitors of specific steps in the mevalonate pathway; atorvastatin (10 μM), FTI-227 (10 μM), GGTI (10 μM), YM-53601 (20 μM), AY-9944 (10 μM), fatostatin (20 μM), 24-hydroxycholesterol (10 μM), T0901317 (10 μM), rosiglitazone (50 μM), GW501516 (10 μM), and efavirenz (10 μM). pThr231Tau/tTau levels were determined by ELISA (mean ± SEM, n ≥ 3).

(D–F) APP^{dp}1-6 neurons were treated with atorvastatin (10 μM), AY-9944 (5 μM), T0901317 (10 μM), or efavirenz (10 μM) for 3 days and lipid analysis was performed to measure (D) free cholesterol (mean ± SEM, n ≥ 8), (E) total cholesterol (mean ± SEM, n ≥ 4), and (F) CE (mean ± SEM, n ≥ 8).

(G) pThr231Tau/tTau levels after 5-day treatment of APP^{dp}1-6 neurons with avasimibe (10 μM) or K604 (25 μM) (mean ± SEM, n ≥ 3).

(H) Treatment of APP^{dp}1-6 neurons with LDL (25 μg/mL) (mean ± SEM, n ≥ 3).

See also Figure S2.

controlled by neuronal cholesterol levels or downstream cholesterol metabolites. To determine whether cholesterol itself or a cholesterol metabolite controls pTau levels, we performed extensive lipid analysis for key selected compounds from previous experiments (Figures 2D–2F and S2L–S2P; Table S5). None of the pTau-reducing drugs affected phospholipid levels (sphingomyelin [SM] and phosphatidylethanolamine [PE]) (Figure S2L). Cholesterol precursor levels were altered in accordance with the enzymatic target of the different compounds (Figures S2M–S2O); atorvastatin reduced desmosterol and lathosterol levels, AY9944 reduced desmosterol and lathosterol levels and increased 7DHC levels, and T0901317 and efavirenz had minor (slightly inhibitory) effects on precursor levels. 24-Hydroxycholesterol (a direct downstream metabolite of cholesterol) was increased in media from efavirenz-treated neurons, was decreased in AY-9944-treated samples, and was unaltered in atorvastatin- and T0901317-treated neurons (Figure S2P). Surprisingly, however, although these compounds behaved as expected, only AY-9944 (that only had minor effect on pTau, Figure 2C) reduced free cholesterol levels (Figure 2D). It is important to note that our iPSC-derived neurons are cultured in media without an exogenous source of cholesterol, and thus neuronal cholesterol levels cannot be compensated by enhanced uptake from the media. Whereas changes in free cholesterol levels were not detected for most compounds, all compounds significantly reduced the levels of total cholesterol (free + esterified cholesterol) (Figure 2E) through a strong reduction of CE (Figure 2F). This finding suggests that conversion of CE to cholesterol compensates for the loss of cholesterol through inhibition of synthesis or induction of export, and reductions in CE, not free cholesterol, mediate the effects of the different compounds on pTau. In line with this observation, direct inhibition of cholesterol esterification by the ACAT inhibitors avasimibe (aka CI-1011) (Figures 2G and S2Q–S2V) (Huttunen et al., 2009, 2010; Lee et al., 1996) or K604 (Figure 2G) also reduced pTau. Exogenous addition of cholesterol and CE (in the form of LDL) increased pThr231Tau/tTau (Figure 2H) levels. Together, our data show that CE regulate pTau levels in human FAD neurons. We investigated the mechanisms underlying CE-dependent regulation of pTau in more detail.

Regulation of pTau by CE Is Correlated with, but Independent of, APP and Aβ

APP^{dp} neurons have an extra copy of APP and increased levels of both Aβ and pTau (Israel et al., 2012; Moore et al., 2015). To understand the relationship between APP copy number, Aβ, and CE-dependent regulation of pTau in more detail, we also treated NDC neurons with the normal two copies of APP with cholesterol-targeting drugs. Simvastatin, atorvastatin, efavirenz,

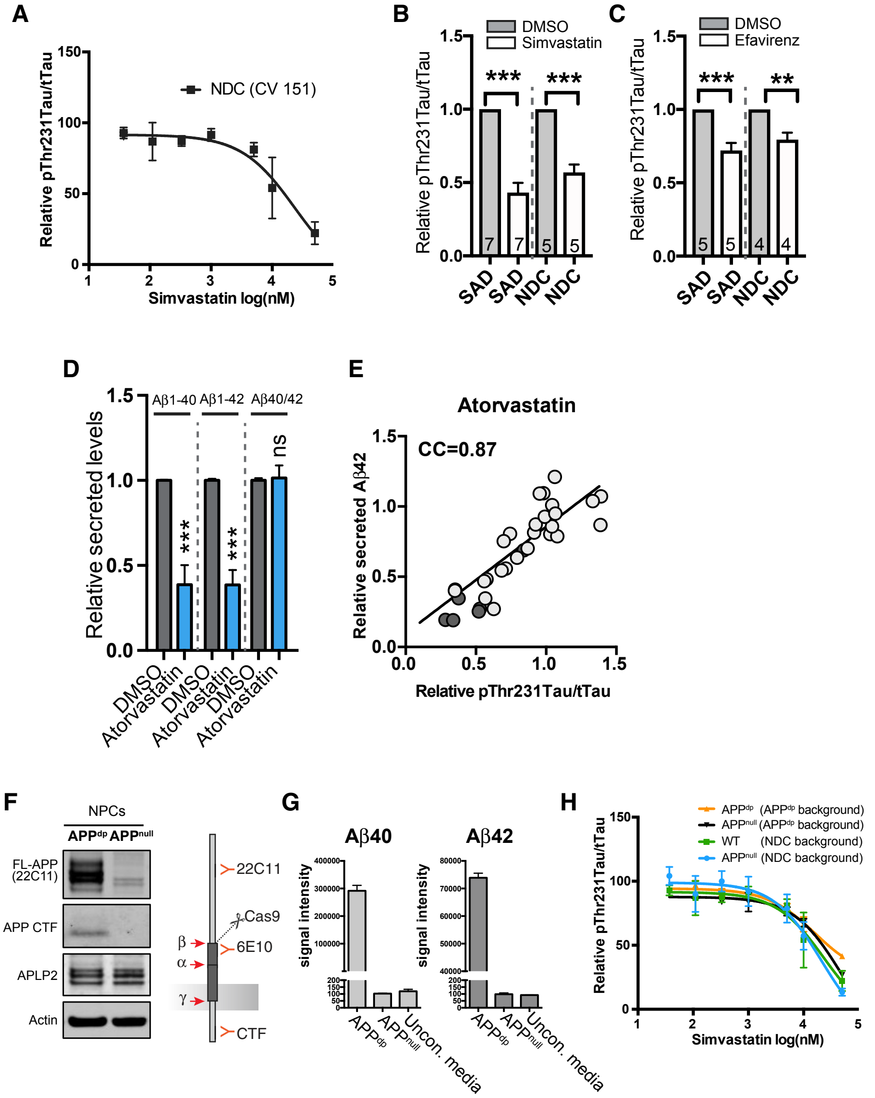

Figure 3. pTau and Aβ Are Co-regulated by CE through Separate Pathways

(A) Dosage effect of simvastatin treatment on pThr231Tau/tTau on non-demented control (NDC) neurons (CV 151 line).

(B and C) Effect of simvastatin (10 μM) (B) or efavirenz (10 μM) treatment (C) on pThr231Tau/tTau in neuronal lines from SAD and NDC subjects (mean ± SEM, number of individual patients indicated in bars).

(D) Secreted Aβ levels from APP^{dp}1-6 neurons treated with atorvastatin (10 μM) for 5 days normalized to DMSO-treated neurons (mean ± SEM, $n \geq 3$).

(E) Correlation of Aβ42 and pThr231Tau/tTau levels in atorvastatin-treated neurons at different time points, dosages, and in different cell lines (light circles, APP^{dp}1-6; dark circles, APP^{dp}2-1). CC, correlation coefficient.

(F and G) Characterization of APP^{null} line. (F) Western blot with antibodies against APP in isogenic APP^{dp} (line APP^{dp} 2-1) and APP^{null} (line APP^{dp}1KO). Full-length APP (FL) and APP CTF are no longer detected in the APP^{dp}1^{null} line. The FL-APP (22C11) cross reacts with APLP2 explaining the remaining signal in the FL-APP (22C11 blot).

(G) ELISA analysis shows an absence of Aβ40 and Aβ42 in conditioned media from the APP^{null} line. Positive control is APP^{dp}1-2, negative control is unconditioned media. The detection antibody for the ELISA is 6E10, as indicated in (F).

(H) Dosage effect of simvastatin treatment on pThr231Tau/tTau on neurons with indicated genotypes (mean ± SEM, $n \geq 3$). Isogenic knockouts used were in an APP^{dp} patient genetic background (lines APP^{dp}1-2 [^{dp}] and APP^{dp}1KO [^{null}] or non-demented control [NDC] genetic background [CV line 151 (wild-type [WT])] and IB6 [^{null}]). Mean ± SEM; $n \geq 5$. See also Figure S3.

and the ACAT inhibitors avasimibe and K604 all reduced pThr231Tau/tTau in the NDC neurons (Figure S3A). For simvastatin, we further tested an extensive dose range and found that it reduced pThr231Tau/Tau in NDC neurons in a similar dose-dependent manner as in APP^{dp} neurons (Figure 3A). A single dose of statin or efavirenz also decreased pThr231Tau/tTau in sporadic AD (SAD) patient- and non-demented control (NDC) neurons (Figures 3B, 3C, S3B, and S3C). pThr231Tau/tTau was also reduced by simvastatin in cultured hippocampal mouse neurons (Figure S3D). Together, these data indicate that CE-dependent regulation of pTau is conserved across individual patients and healthy subjects (and even across species) and is not dependent on baseline APP copy number.

In addition to our findings on pTau, CE reduction has previously been reported to reduce Aβ levels (Hutter-Paier et al., 2004; Huttunen et al., 2009; Puglielli et al., 2001), and Aβ secretion from our iPSC-derived neurons was also decreased by statins (Figure 3D). We observed a strong correlation between Aβ42 secretion and pThr231Tau/tTau in response to atorvastatin treatment across time points and drug doses (Figures 3E and S3E).

To test whether the effect of CE on pTau was mediated by alterations in APP processing and/or the reduction of Aβ, we generated an isogenic APP^{null} line in an APP^{dp} patient genetic background (Figures 3F, 3G, and S3F–S3H). No APP expression or Aβ secretion was detected in the APP^{null} neurons (Figures 3F and 3G). pThr231Tau/tTau levels were reduced at baseline in the APP^{null} neurons (Figure S3I). Interestingly, in these APP^{null} neurons, simvastatin still reduced pThr231Tau/tTau in a dose-dependent manner identical to that of its isogenic control lines (Figure 3H). Similarly, simvastatin reduced pThr231Tau/tTau the same in a previously generated isogenic set of NDC (APP^{wt}) and APP^{null} neurons (CV line 151 and IB6) (Fong et al., 2018) (Figure 3H). Together, these data show that CE regulate both pTau and Aβ, but regulation of pTau by CE is APP- and Aβ-independent.

The Effect of CE on Aβ Is Mediated by a Cholesterol-Binding Domain in APP

To understand the respective pathways by which CE regulate pTau and Aβ, and to verify that these pathways are indeed

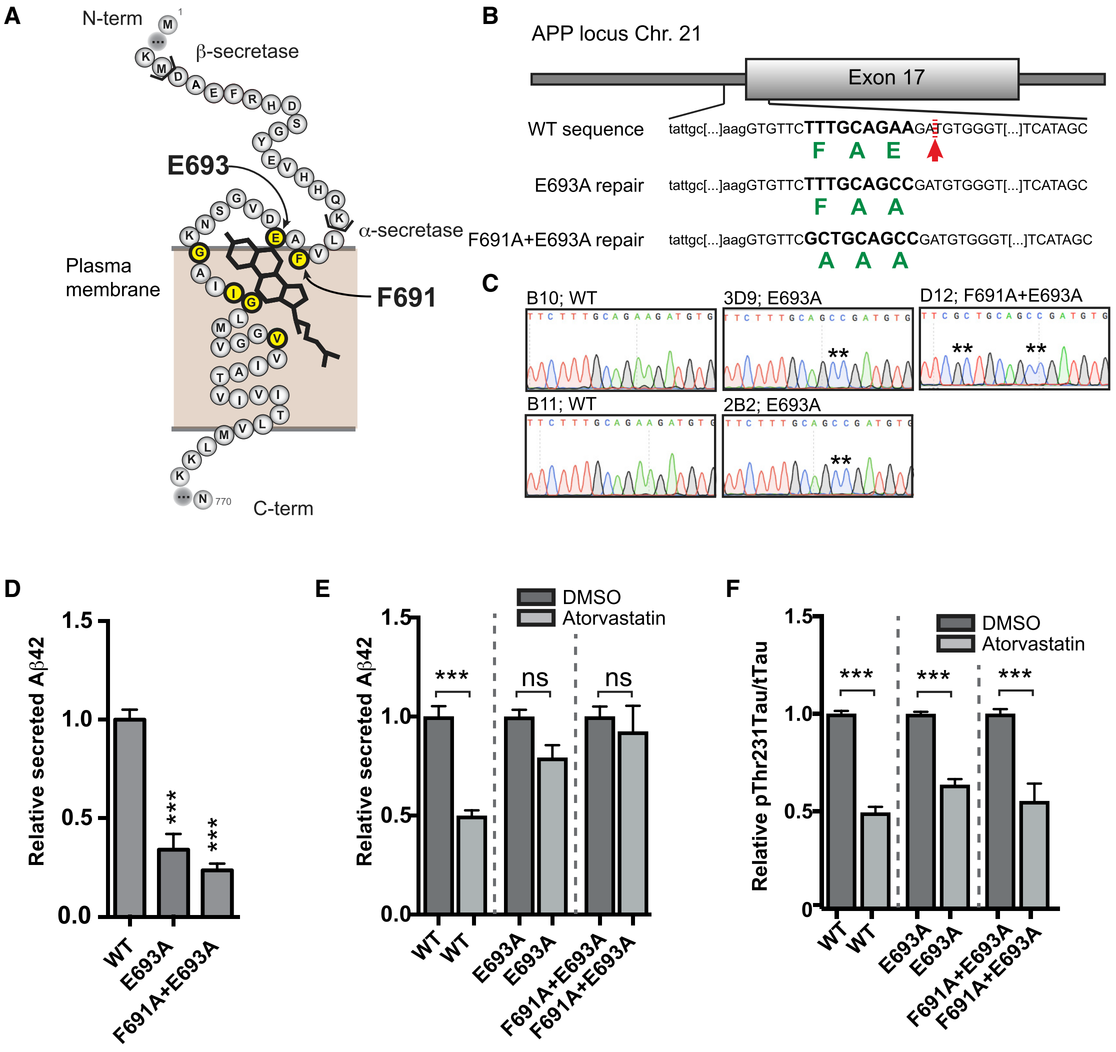

Figure 4. Regulation of Aβ by CE Is Mediated by a Cholesterol Binding Domain in APP

(A) Schematic representation of the transmembrane domain of APP with amino acids essential for cholesterol binding indicated in yellow.

(B) Schematic overview of the gene-editing strategy to generate APP-Δcholesterol lines. Green indicates amino acid sequence. Red arrow indicates CRISPR/Cas9 cut site.

(C) Sequencing results verifying correct incorporation of desired mutations in the APP-Δcholesterol lines. Two E693A (line 3D9 and 2B2) and one F691A+E693A line (line D12) were generated, as well as two non-gene-edited, but clonally expanded, WT lines (B10 and B11).

(D–F) Measurements made using APP-Δcholesterol neurons with the following genotypes: WT (average from 2 independent lines), E693A (2 independent lines), and F691A+E693A (1 line) (mean ± SEM, n ≥ 3 per line). (D) Relative secreted Aβ42 levels in conditioned media from purified CD184$^-$, CD44$^-$, and CD24$^+$ neurons (mean ± SEM, n ≥ 3 per line). (E) Relative secreted Aβ42 in response to atorvastatin treatment (10 μM, 3 days) (mean ± SEM, n ≥ 3 per line) (F) pThr231Tau/tTau in response to atorvastatin treatment (10 μM, 3 days) in APP-Δcholesterol neurons (mean ± SEM, n ≥ 3 per line).

See also Figure S4.

separate, we first studied the relationship between CE and Aβ secretion in more detail. We hypothesized that the effect of CE on Aβ secretion could be mediated by a recently identified cholesterol-binding domain in APP β-CTF (Barrett et al., 2012). We used CRISPR/Cas9 to mutate the cholesterol-binding domain in the endogenous APP locus (Figures 4A–4C, S4A, and S4B) and created two mutations that had previously been shown to abolish APP β-CTF-cholesterol interactions (Barrett et al., 2012), APP E693A and APP F691A+E693A. We observed that Aβ42 secretion in these isogenic APP-Δcholesterol lines was reduced under steady state conditions (Figure 4D) indicating that the cholesterol-binding domain affects APP processing and Aβ secretion. More importantly, atorvastatin treatment (Figure 4E) did not affect Aβ42 secretion in these neurons indicating that the effect of lowering CE on Aβ42 is mediated by the cholesterol-binding domain in APP. While Aβ secretion was no longer regulated by atorvastatin in these neurons, atorvastatin still decreased pT231Tau/tTau ratio (in an identical manner as in their isogenic wild-type controls) (Figure 4F), again confirming that the effect of CE on pTau and the effect of CE on Aβ are regulated through two separate pathways. We next sought to determine how CE regulate pTau.

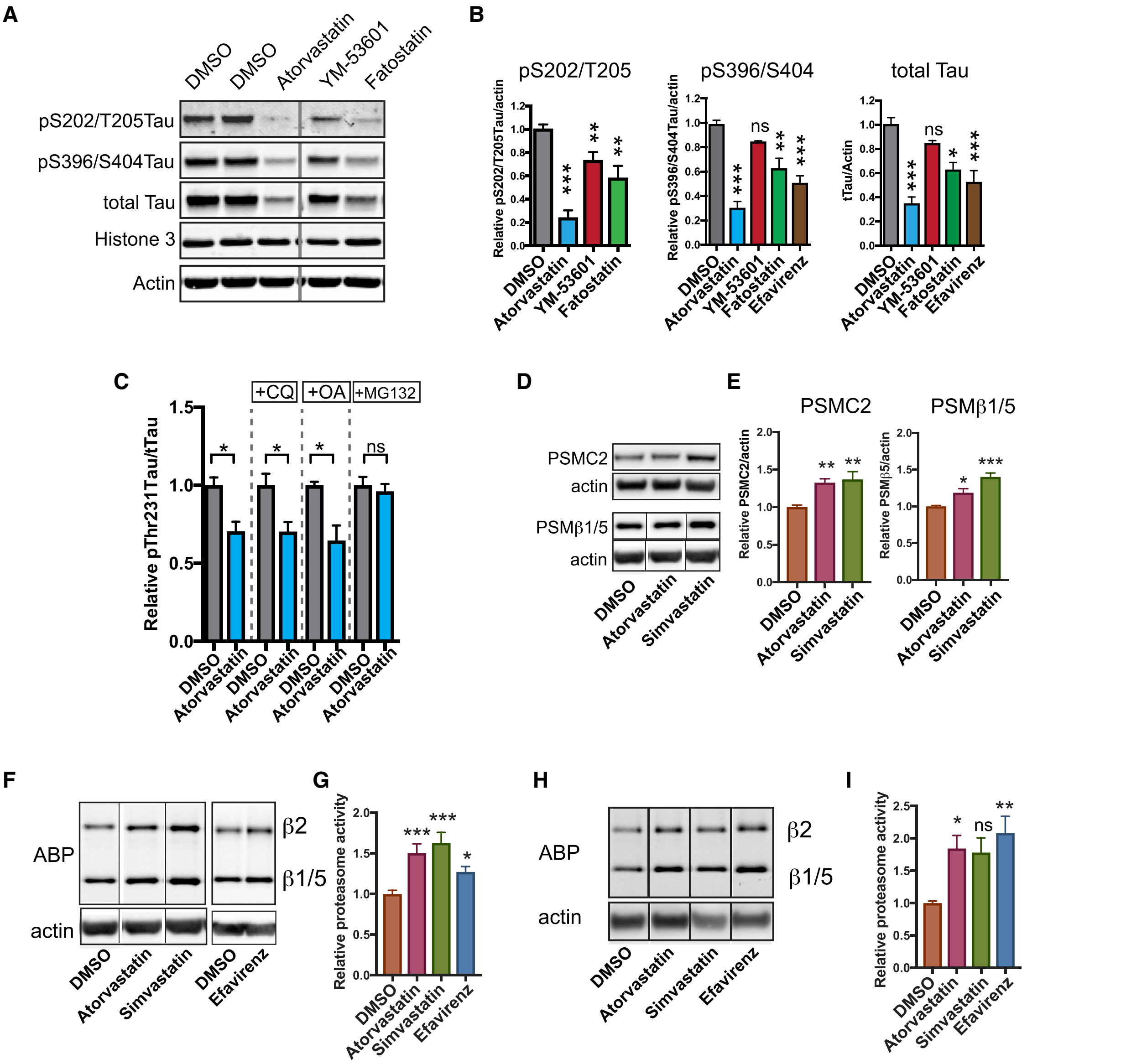

Figure 5. Regulation of pTau by CE Is Mediated by the Proteasome

(A and B) The effect of CE lowering treatments on Tau levels and Tau phosphorylation as assessed by western blot (A), quantified in (B) (mean ± SEM, n ≥ 3). Image is a composite of different loading positions on same blot, stitch is indicated by vertical line.

(C) pThr231Tau/tTau levels in APP^{dp}1-6 neurons co-treated with atorvastatin (10 μm) and a lysosomal inhibitor (chloroquine, CQ 25 μM), a phosphatase inhibitor (okadaic acid, 1.25nM) or a proteasome inhibitor (MG132, 5 μM) for 3 days as measured by ELISA (mean ± SEM, n ≥ 3).

(D and E) APP^{dp}1-6 neurons were treated for 3 days with DMSO, simvastatin (10 μM) or atorvastatin (10 μM) and levels of proteasome subunits PSMC2 and PSMβ1/5 were assessed by western blot (D). Quantified in (E) (mean ± SEM n ≥ 3). PSMβ1/5/actin image is a composite of different loading positions on same blot, stiches are indicated by vertical line.

(F–I) APP^{dp}1-6 (F and G) or NDC CV4a (H and I) neurons were treated for 3 days with DMSO, simvastatin (10 μM), atorvastatin (10 μM), or efavirenz (10 μM) and incubated with a proteasome activity binding probe (ABP) for 1 h. Cells were lysed, run on SDS-page, and ABP fluorescence from the gel was determined (mean ± SEM, n ≥ 5). (G) Quantification of the western blots from (F). (I) Quantification of western blots from (H). Images are composite of different loading positions on same blot, stiches are indicated by vertical lines.

See also Figure S5.

The Effect of CE on pTau Levels Is Mediated by the Proteasome

Previous reports indicate that accumulation of pTau in FAD neurons can be downstream of altered proteostatic regulation of (p)Tau (Moore et al., 2015). To assess whether CE affect the proteostatic regulation of pTau, we performed quantitative western blot on APP^{dp} neurons treated with different CE-targeting drugs. In addition to a reduction of pS396/S404Tau and pS202/T205Tau, CE reduction also reduced total Tau (Figures 5A, 5B, and S5A). The reduction of pTau and total Tau by our

treatments was not explained by a specific loss of neurons in the cultures (Figures S5B–S5D). The reduction of both pTau as well as tTau could indicate a proteostatic regulatory event, rather than altered Tau phosphorylation and dephosphorylation events. This was further substantiated by the finding that activity of GSK3β (a major Tau kinase) was not affected by statin-mediated CE reduction (Figure S5E). Next, we attempted to rescue the effect of CE reduction on pThr231Tau/tTau with inhibitors of phosphatase activity (okadaic acid), proteasomal (MG132), and/or or lysosomal and autophagosomal (chloroquine) degradation (Figures 5C and S5F–S5M). Only proteasomal inhibition abrogated the decrease in pThr231Tau/tTau ratio induced by atorvastatin (Figures 5C and 5N), indicating that the effect of CE on pTau is mediated by the proteasome. Interestingly, in addition to cholesterol-synthetic genes, we found that the 26S proteasome regulatory subunit 7 (PSMC2) was also upregulated after statin treatment in NDC neurons (Figure S2D; Table S2). We validated by western blot in APP^{dp} neurons that statin treatment increased the levels of proteasomal subunit PSMC2 (Figures 5D and 5E). Levels of another proteasome subunit, proteasome subunit beta type-5 (PSMβ5) in the core of the proteasome, were also increased (Figures 5D and 5E). This statin-dependent increase in proteasome levels is not mediated by transcriptional upregulation of proteasomal subunits (Figure S5N). Using a proteasome activity binding probe (Berkers et al., 2007; Leestemaker et al., 2017), we found that CE reduction through either statins or efavirenz increased total cellular proteasome activity in both APP^{dp} and NDC neurons (Figures 5F–5I). The effect of CE on proteasome function was not mediated by mechanistic target of rapamycin (mTor) (Figures S5O–S5Q). Together, our data show that reducing neuronal CE enhances proteasome levels, increases total cellular proteasome activity, and induces the proteasomal degradation of pTau indicative of a CE-proteasome-Tau axis.

CYP46A1 Activation Is a Neuron-Specific CE-Reducing Approach that Is Better Tolerated by Astrocytes Than HMGCR Inhibitors (Statins)

Our data indicate that neuronal CE are a potential therapeutic target to activate the proteasome and prevent aberrant pTau accumulation in AD. To identify possible adverse effects of candidate CE-lowering strategies on other non-neuronal brain cells, we tested the toxicity of selected compounds on iPSC-derived astrocytes. All iPSC-derived astrocytes displayed radial morphology of astrocytes and expressed the glial marker GFAP, while the neural stem cell marker SOX2 was not expressed (Figure 6A). We concentrated on two different drugs that currently have Food and Drug Administration (FDA) approval for other indications, statins (to lower cholesterol synthesis) and efavirenz (that activates the neuron-specific enzyme CYP46A1). Already, at low concentrations, simvastatin and atorvastatin induced major astrocyte cell death (Figures 6B, 6C, S6A, and S6B), whereas efavirenz did not affect astrocyte viability even at high doses (Figures 6D and S6C). 24-Hydroxycholesterol was not detected in astrocyte media before or after treatment with efavirenz. Efavirenz increased 24-hydroxycholesterol secretion from APP^{dp} and APP^{null} neurons in a dose-dependent manner that correlated well with its effect on pThr231Tau/tTau in these neurons (Figures 6D and S6D). Together, these data show that allosteric activation of CYP46A1 is a neuron-specific CE- and pTau-lowering treatment with less adverse effects on astrocytes and provides a therapeutic approach to reduce pTau accumulation in early AD-neurons.

DISCUSSION

Here, we used AD-patient iPSC-derived neurons in a phenotypic drug screen to identify compounds that reduce aberrant pTau accumulation in FAD neurons (Israel et al., 2012; Moore et al., 2015; Muratore et al., 2014; Ochalek et al., 2017). From a library of >1,600 compounds, we successfully identify 42 compounds that reduced pTau levels, including six previously reported modulators of pTau and 36 novel pTau targeting compounds (Figure 1). From the 42 identified compounds, we selected cholesterol-targeting compounds (statins) to study in more detail. Cholesterol metabolism has previously been implicated in AD (Di Paolo and Kim, 2011; Puglielli et al., 2003) and CE, the esterified storage products of cholesterol, accumulate in AD patient brains (Chan et al., 2012) and in APP-transgenic mice (Chan et al., 2012; Tajima et al., 2013; Yang et al., 2014). We show that reducing CE, through a number of mechanistically different drugs, reduces levels of pTau at multiple phosphorylation epitopes in both FAD, SAD, and NDC subject neurons. CE have previously also been shown to regulate APP processing and Aβ generation (Hutter-Paier et al., 2004; Huttunen et al., 2009; Puglielli et al., 2001), and we confirmed that CE also regulate Aβ secretion from human iPSC-derived AD patient neurons. Interestingly, we find that the effect of CE on Aβ is independent of the effect of CE on pTau (Figures 3 and 4), and pTau and Aβ are thus co-regulated by CE through independent pathways. Similar co-regulation of Aβ and Tau through independent pathways have recently been shown for ApoE (Wang et al., 2018) and the retromer complex (Young et al., 2018). These findings, together with our findings, reinforce the notion that common upstream (pathogenic) pathways in SAD, such as CE, can drive increased levels of pTau and Aβ through separate pathways (Small and Duff, 2008), rather than only through a direct linear pathway directly from Aβ to Tau.

Pathways that Mediate the Effect of CE on pTau and Aβ

We investigated the separate pathways that underlie CE-dependent regulation of pTau and Aβ, respectively, in early AD neurons. We find that the effect of CE on Aβ is mediated by a cholesterol binding-site in APP, whereas the effect of CE on pTau is mediated by the proteasome. Surprisingly, in our system, we did not observe significant differences in free cholesterol levels for the treatments that reduced CE, Aβ, and pTau. As also previously observed (Puglielli et al., 2001), these data indicate that CE mediates the effect of cholesterol-targeting drugs on Aβ. We show that the effect of CE on Aβ is mediated by a domain in APP that has previously been shown to bind cholesterol (Barrett et al., 2012), possibly suggesting that this cholesterol-binding domain can also sense CE. Alternatively, localized reductions in cholesterol in specific domains such as lipid rafts (Ehehalt et al., 2003), specific organelles, or localized changes in *de novo* synthesized cholesterol under the detection limit of our measurements could mediate the effect of statins on APP processing via the APP-cholesterol binding domain. In regard to CE-dependent regulation of pTau, we find that reduction of

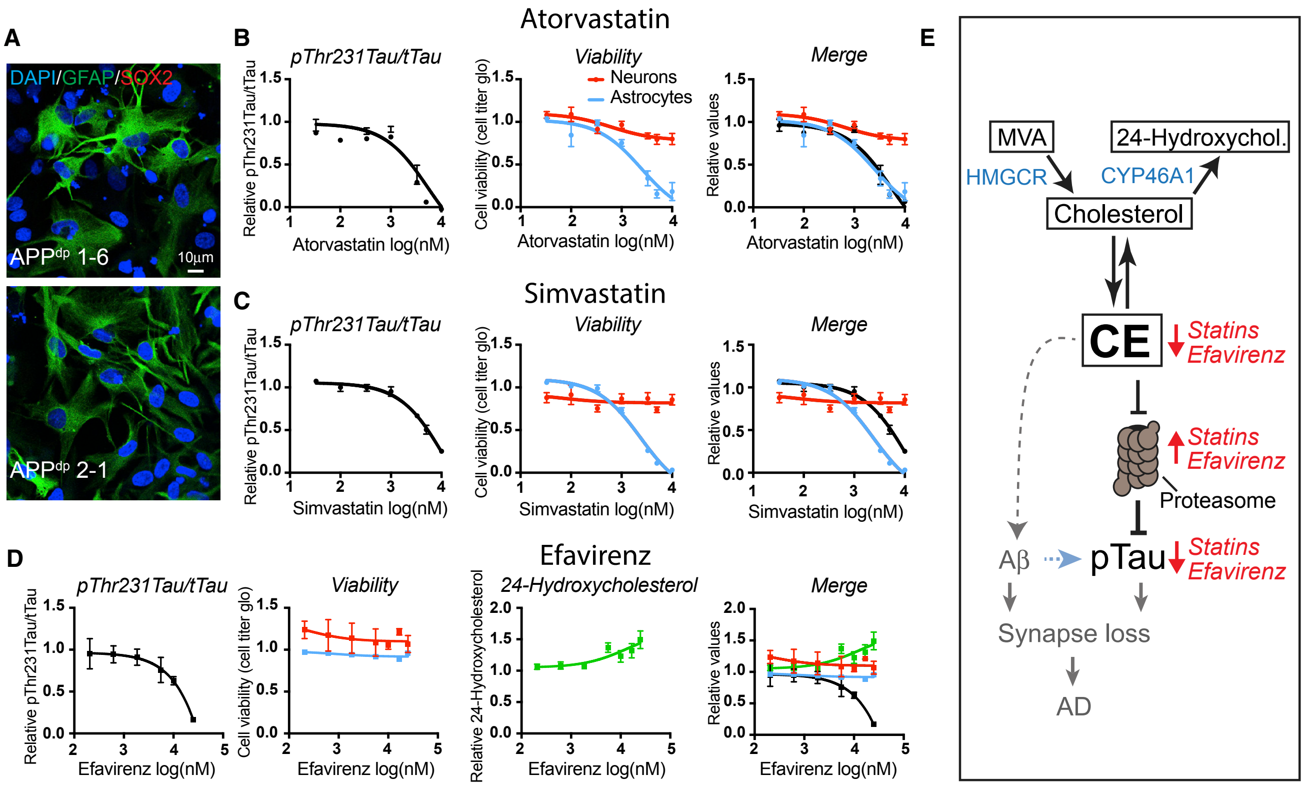

Figure 6. CYP46A1 Activation Is a Neuron-Specific CE-Reducing Approach that Is Better Tolerated by Astrocytes

(A) iPSC-derived astrocytes were fixed and stained with indicated antibodies. Scale bar, 10 μm.

(B–D) iPSC-derived APP^{dp}1-6 astrocytes were treated for 3 days with increasing concentrations of (B) atorvastatin, (C) simvastatin, and (D) efavirenz, and viability was measured (cell titer glo). Astrocytic viability (blue line) was plotted against results from Figures 1D and S1A for statins (neuronal viability and pThr231Tau/Tau ratio). For efavirenz, dose responses to measure pThr231Tau/tTau and neuronal viability were performed in APP^{dp}1-6 neurons (mean ± SEM, n $\geq$ 3–6).

(E) Model depicting the relationship between CE, pTau, and Aβ in early AD neurons. Statins reduce CE levels through inhibition of the cholesterol-synthetic pathway, while efavirenz enhances the turnover of cholesterol to 24-hydroxycholesterol that causes conversion of CE to cholesterol and a reduction in CE. Reduced CE cause proteasomal upregulation and degradation of pTau. In a correlated, but independent pathway, CE regulate APP processing and Aβ generation.

See also Figure S6.

CE increases the level of proteasomal subunits, overall proteasomal activity, and proteasomal degradation of pTau (see model Figure 6E). This indicates a neuronal CE-proteasome-pTau axis that regulates turnover of neuronal pTau. The effect of CE on proteasome levels is not mediated by enhanced transcription of proteasomal subunits, and the exact mechanism by which CE control proteasomal activity needs to be further determined. Interestingly, in other model systems, lipid droplets (the storage site of CE) have been shown to be active signaling organelles that regulate proteasome activity (Arrese et al., 2014; Keembiyehetty et al., 2011). The neuronal proteasome also associates with plasma membranes (Ramachandran and Margolis, 2017), offering another possible neuron-specific site of convergence between CE and the proteasome. CE-dependent regulation of the proteasome is also relevant for other neurodegenerative diseases in which altered cholesterol-homeostasis and protein aggregation occurs such as Niemann-Pick type C and Huntington disease (Karasinska and Hayden, 2011). Another open issue is why pTau is regulated by CE. Direct coupling of CE to pTau levels could indicate a pathway that coordinates the speed of Tau-regulated axonal transport or growth, with the availability of (stored) neuronal cholesterol required for membrane growth or synapse formation. Overall, our data here indicate that CE regulate pTau and Aβ by two separate pathways and suggests that CE could be an upstream driver of both Aβ secretion and pTau accumulation.

CE and AD

Our findings provide a mechanistic explanation for how changes in CE, induced by APP mutations or SAD genetic risk variants in APP (Chan et al., 2012; Tajima et al., 2013; Yang et al., 2014) could contribute to Tau pathology. Interestingly, CE production is overactive in human SAD fibroblasts (Pani et al., 2009) and APOE, the major genetic risk factor for SAD, acts as a CE transporter in brain (Liu et al., 2013; Michikawa et al., 2000; Minagawa et al., 2009). Our findings in human iPSC-derived AD neurons indicate that alterations in neuronal CE could drive pTau accumulation and are supported by previous *in vivo* observations in mouse models showing that statins reduce NFT load in animal models of tauopathy (Boimel et al., 2009), and genetic inhibition of cholesterol esterification by ACAT1 reduces tauopathy in an AD-mouse model (Shibuya et al., 2015), indicating that the

interactions between CE and Tau are conserved in the adult (mouse) brain. We describe several pharmaceutical strategies to reduce CE in early AD neurons including LXR target gene-mediated cholesterol export, conversion of cholesterol to hydroxycholesterol by CYP46A1 activators, and direct inhibition of cholesterol esterification by ACAT inhibitors. In humans, long-term statin usage has been shown to correlate with reduced AD incidence in some studies (Shepardson et al., 2011a, 2011b; Zissimopoulos et al., 2017), although the underlying mechanisms have been debated. Statins also reduce pTau levels in CSF from AD patients (Riekse et al., 2006). Here, we show that the effects of statins on pTau can be directly mediated by CE and are not merely a consequence of altered APP processing or peripheral effects of cholesterol-targeting drugs. However, our data do not indicate that statins are the best candidate drugs for targeting Tau accumulation in AD. Statins affect the levels of pTau in human neurons only at relatively high concentrations (Figure 1) unlikely reached in human brain (Björkhem-Bergman et al., 2011) and have strong adverse effects on human astrocytes at these high concentrations (Figure 6). We cannot exclude the possibility that *in vivo* statins lower neuronal CE through indirect effects on astrocytic cholesterol and/or CE production, but our data do indicate that additional potency could be gained from enhanced targeting of neuronal CE. We show that allosteric activators of CYP46A1 could provide a neuron-specific approach to reduce CE and pTau in early AD neurons. Regulation of pTau by CYP46A1 is also conserved in adult (mice) brains where genetic inhibition of CYP46A1 enhances abnormal phosphorylation of Tau (Djelti et al., 2015), and overexpression of CYP46A1 in transgenic Tau mice rescues cognitive decline (Burlot et al., 2015).

We targeted CYP46A1 through allosteric activation by the small molecule efavirenz (Anderson et al., 2016) and show that efavirenz reduces pTau in early human AD neurons without affecting astrocyte viability. Efavirenz, originally marketed as an HIV-medication (brand name Sustiva), has recently also been shown to reduce amyloid pathology in AD mice (Mast et al., 2017b) indicating that efavirenz could possibly be repurposed for AD. However, when given to HIV patients at high doses, efavirenz has significant adverse effects that include neurotoxicity (Apostolova et al., 2017). Major adverse effects were not observed in mice at lower concentrations of efavirenz that sufficed to alter brain cholesterol metabolism and reduce amyloid pathology, indicative of an appropriate therapeutic window (Mast et al., 2017b). Other allosteric activators of CYP46A1 have also recently been identified (Mast et al., 2017a). The *in vivo* data of CYP46A1 activators on inhibitors of amyloid pathology (Mast et al., 2017b), together with our findings that CYP46A1 activators reduce Tau accumulation in human AD neurons, support the development of allosteric activators of CYP46A1 as therapies for AD.

In conclusion, using high-throughput phenotypic screening, we identified >40 FDA-approved drugs with diverse biological targets that reduce pTau in early AD neurons. By pathway mapping, we identify a CYP46A1-CE-Tau axis as a druggable pathway in early AD. We find that reducing CE potently decreases neuronal pTau levels through proteasomal upregulation and degradation of pTau in an APP- and Aβ-independent manner. In a separate pathway, Aβ secretion is regulated by CE through a cholesterol-binding domain in APP. We find that allosteric activation of CYP46A1 is a neuron-specific CE-lowering strategy that is well tolerated by human astrocytes. Together, our data indicate CE as a dual upstream regulator of pTau and Aβ, and we propose CE-reduction, particularly through CYP46A1 activation, as a therapeutic approach to independently reduce accumulation of pTau and Aβ in AD patients.

STAR★METHODS

Detailed methods are provided in the online version of this paper and include the following:

- KEY RESOURCES TABLE
- CONTACT FOR REAGENT AND RESOURCE SHARING
- EXPERIMENTAL MODEL AND SUBJECT DETAILS
  - Cultured Mouse Cortical Neurons
  - Human iPSC Derived Cell lines
- METHOD DETAILS
  - Reagents
  - Phenotypic high-throughput screen
  - Aβ, pThr231Tau/total Tau, pSer9GSK3b/total Gsk3b, Phospho-4E-BP1(Thr37/46)/total 4E-BP1 ELISA Measurements
  - Cell viability
  - Mass spectrometry sample preparation and LC-MS-MS
  - Lipid measurements
  - Microscopy
  - RNA expression analysis
  - Quantitative western blot
  - Proteasomal levels and activity
  - Vectors and viral transduction
- QUANTIFICATION AND STATISTICAL ANALYSIS
  - Statistical Analysis

SUPPLEMENTAL INFORMATION

Supplemental Information includes six figures and six tables and can be found with this article online at https://doi.org/10.1016/j.stem.2018.12.013.

ACKNOWLEDGMENTS

We would like to acknowledge Majid Ghassemian and the UCSD Biomolecular and Proteomic Mass Spectrometry Facility for assistance with the generation of the mass spectrometry data. We would like to acknowledge Eline Kompanje, Ruben Ruiz, and Robbert Zalm for experimental support; Somasish Dastidar for help with the mice neuronal cultures; and Matthijs Verhage, Vivi Heine, and Charlotte Teunissen for generously sharing reagents and equipment. We would like to acknowledge Elsa Rodrigues (University of Lisbon) for the generous sharing of CYP46A1 plasmids. R.v.d.K. was supported by an Alzheimer Netherlands Fellowship (WE.15-2013-01) and an ERC Marie Curie International Outgoing fellowship (622444, APPtoTau). V.F.L. was supported by an NIH T32 training grant (5T32AG000216-24). This work is supported by NIA 1RF1AG048083-01 and CIRM RB5-07011 grants to L.S.G. We would like to dedicate this paper to our friend and colleague, Daniel Williams.

AUTHOR CONTRIBUTIONS

R.v.d.K. and V.F.L. designed and performed the experiments and wrote the paper together with L.S.B.G. C.M.H., D.A.W., and A.G.B. performed the drug screen. L.K.F. generated the APP knockout lines. Y.L. and H.O.

performed the experiments with proteasome binding probe. E.S. and M.G. performed lipid measurements for figures in the manuscript. J.F.B. and J.B.H. performed pilot measurements for cholesterol precursors. S.L.W. and K.D.R. were involved in screen design and compound identification.

DECLARATION OF INTERESTS

The authors declare no competing interests.

Received: March 9, 2018
Revised: September 26, 2018
Accepted: December 17, 2018
Published: January 24, 2019

REFERENCES

Anderson, K.W., Mast, N., Hudgens, J.W., Lin, J.B., Turko, I.V., and Pikuleva, I.A. (2016). Mapping of the allosteric site in cholesterol hydroxylase CYP46A1 for efavirenz, a drug that stimulates enzyme activity. J. Biol. Chem. *291*, 11876–11886.

Apostolova, N., Blas-Garcia, A., Galindo, M.J., and Esplugues, J.V. (2017). Efavirenz: what is known about the cellular mechanisms responsible for its adverse effects. Eur. J. Pharmacol. *812*, 163–173.

Arrese, E.L., Saudale, F.Z., and Soulages, J.L. (2014). Lipid droplets as signaling platforms linking metabolic and cellular functions. Lipid Insights *7*, 7–16.

Barrett, P.J., Song, Y., Van Horn, W.D., Hustedt, E.J., Schafer, J.M., Hadziselimovic, A., Beel, A.J., and Sanders, C.R. (2012). The amyloid precursor protein has a flexible transmembrane domain and binds cholesterol. Science *336*, 1168–1171.

Berkers, C.R., van Leeuwen, F.W.B., Groothuis, T.A., Peperzak, V., van Tilburg, E.W., Borst, J., Neefjes, J.J., and Ovaa, H. (2007). Profiling proteasome activity in tissue with fluorescent probes. Mol. Pharm. *4*, 739–748.

Björkhem-Bergman, L., Lindh, J.D., and Bergman, P. (2011). What is a relevant statin concentration in cell experiments claiming pleiotropic effects? Br. J. Clin. Pharmacol. *72*, 164–165.

Boimel, M., Grigoriadis, N., Lourbopoulos, A., Touloumi, O., Rosenmann, D., Abramsky, O., and Rosenmann, H. (2009). Statins reduce the neurofibrillary tangle burden in a mouse model of tauopathy. J. Neuropathol. Exp. Neurol. *68*, 314–325.

Brownjohn, P.W., Smith, J., Portelius, E., Serneels, L., Kvartsberg, H., De Strooper, B., Blennow, K., Zetterberg, H., and Livesey, F.J. (2017). Phenotypic screening identifies modulators of amyloid precursor protein processing in human stem cell models of Alzheimer's disease. Stem Cell Reports *8*, 870–882.

Buerger, K., Teipel, S.J., Zinkowski, R., Blennow, K., Arai, H., Engel, R., Hofmann-Kiefer, K., McCulloch, C., Ptok, U., Heun, R., et al. (2002). CSF tau protein phosphorylated at threonine 231 correlates with cognitive decline in MCI subjects. Neurology *59*, 627–629.

Burlot, M.-A., Braudeau, J., Michaelsen-Preusse, K., Potier, B., Ayciriex, S., Varin, J., Gautier, B., Djelti, F., Audrain, M., Dauphinot, L., et al. (2015). Cholesterol 24-hydroxylase defect is implicated in memory impairments associated with Alzheimer-like Tau pathology. Hum. Mol. Genet. *24*, 5965–5976.

Chan, R.B., Oliveira, T.G., Cortes, E.P., Honig, L.S., Duff, K.E., Small, S.A., Wenk, M.R., Shui, G., and Di Paolo, G. (2012). Comparative lipidomic analysis of mouse and human brain with Alzheimer disease. J. Biol. Chem. *287*, 2678–2688.

Choi, S.H., Kim, Y.H., Hebisch, M., Sliwinski, C., Lee, S., D'Avanzo, C., Chen, H., Hooli, B., Asselin, C., Muffat, J., et al. (2014). A three-dimensional human neural cell culture model of Alzheimer's disease. Nature *515*, 274–278.

D'Antonio, M., Woodruff, G., Nathanson, J.L., D'Antonio-Chronowska, A., Arias, A., Matsui, H., Williams, R., Herrera, C., Reyna, S.M., Yeo, G.W., et al. (2017). High-throughput and cost-effective characterization of induced pluripotent stem cells. Stem Cell Reports *8*, 1101–1111.

de Jong, A., Schuurman, K.G., Rodenko, B., Ovaa, H., and Berkers, C.R. (2012). Fluorescence-based proteasome activity profiling. Methods Mol. Biol. *803*, 183–204.

Di Paolo, G., and Kim, T.-W. (2011). Linking lipids to Alzheimer's disease: cholesterol and beyond. Nat. Rev. Neurosci. *12*, 284–296.

Dickey, C.A., Ash, P., Klosak, N., Lee, W.C., Petrucelli, L., Hutton, M., and Eckman, C.B. (2006). Pharmacologic reductions of total tau levels; implications for the role of microtubule dynamics in regulating tau expression. Mol. Neurodegener. *1*, 6.

Djelti, F., Braudeau, J., Hudry, E., Dhenain, M., Varin, J., Bièche, I., Marquer, C., Chali, F., Ayciriex, S., Auzeil, N., et al. (2015). CYP46A1 inhibition, brain cholesterol accumulation and neurodegeneration pave the way for Alzheimer's disease. Brain *138*, 2383–2398.

Ehehalt, R., Keller, P., Haass, C., Thiele, C., and Simons, K. (2003). Amyloidogenic processing of the Alzheimer beta-amyloid precursor protein depends on lipid rafts. J. Cell Biol. *160*, 113–123.

Foley, P. (2010). Lipids in Alzheimer's disease: a century-old story. Biochim. Biophys. Acta *1801*, 750–753.

Fong, L.K., Yang, M.M., Dos Santos Chaves, R., Reyna, S.M., Langness, V.F., Woodruff, G., Roberts, E.A., Young, J.E., and Goldstein, L.S.B. (2018). Full-length amyloid precursor protein regulates lipoprotein metabolism and amyloid-β clearance in human astrocytes. J. Biol. Chem. *293*, 11341–11357.

Giera, M., Plössl, F., and Bracher, F. (2007). Fast and easy in vitro screening assay for cholesterol biosynthesis inhibitors in the post-squalene pathway. Steroids *72*, 633–642.

Gore, A., Li, Z., Fung, H.-L., Young, J.E., Agarwal, S., Antosiewicz-Bourget, J., Canto, I., Giorgetti, A., Israel, M.A., Kiskinis, E., et al. (2011). Somatic coding mutations in human induced pluripotent stem cells. Nature *471*, 63–67.

Hamilton, L.K., Dufresne, M., Joppé, S.E., Petryszyn, S., Aumont, A., Calon, F., Barnabé-Heider, F., Furtos, A., Parent, M., Chaurand, P., and Fernandes, K.J.L. (2015). Aberrant lipid metabolism in the forebrain niche suppresses adult neural stem cell proliferation in an animal model of Alzheimer's disease. Cell Stem Cell *17*, 397–411.

Han, D.H., Na, H.-K., Choi, W.H., Lee, J.H., Kim, Y.K., Won, C., Lee, S.-H., Kim, K.P., Kuret, J., Min, D.-H., and Lee, M.J. (2014). Direct cellular delivery of human proteasomes to delay tau aggregation. Nat. Commun. *5*, 5633.

Hutter-Paier, B., Huttunen, H.J., Puglielli, L., Eckman, C.B., Kim, D.Y., Hofmeister, A., Moir, R.D., Domnitz, S.B., Frosch, M.P., Windisch, M., and Kovacs, D.M. (2004). The ACAT inhibitor CP-113,818 markedly reduces amyloid pathology in a mouse model of Alzheimer's disease. Neuron *44*, 227–238.

Huttunen, H.J., Peach, C., Bhattacharyya, R., Barren, C., Pettingell, W., Hutter-Paier, B., Windisch, M., Berezovska, O., and Kovacs, D.M. (2009). Inhibition of acyl-coenzyme A: cholesterol acyl transferase modulates amyloid precursor protein trafficking in the early secretory pathway. FASEB J. *23*, 3819–3828.

Huttunen, H.J., Havas, D., Peach, C., Barren, C., Duller, S., Xia, W., Frosch, M.P., Hutter-Paier, B., Windisch, M., and Kovacs, D.M. (2010). The acyl-coenzyme A: cholesterol acyltransferase inhibitor CI-1011 reverses diffuse brain amyloid pathology in aged amyloid precursor protein transgenic mice. J. Neuropathol. Exp. Neurol. *69*, 777–788.

Ikonen, E. (2008). Cellular cholesterol trafficking and compartmentalization. Nat. Rev. Mol. Cell Biol. *9*, 125–138.

Israel, M.A., Yuan, S.H., Bardy, C., Reyna, S.M., Mu, Y., Herrera, C., Hefferan, M.P., Van Gorp, S., Nazor, K.L., Boscolo, F.S., et al. (2012). Probing sporadic and familial Alzheimer's disease using induced pluripotent stem cells. Nature *482*, 216–220.

Kamisuki, S., Mao, Q., Abu-Elheiga, L., Gu, Z., Kugimiya, A., Kwon, Y., Shinohara, T., Kawazoe, Y., Sato, S., Asakura, K., et al. (2009). A small molecule that blocks fat synthesis by inhibiting the activation of SREBP. Chem. Biol. *16*, 882–892.

Karasinska, J.M., and Hayden, M.R. (2011). Cholesterol metabolism in Huntington disease. Nat. Rev. Neurol. *7*, 561–572.

Keck, S., Nitsch, R., Grune, T., and Ullrich, O. (2003). Proteasome inhibition by paired helical filament-tau in brains of patients with Alzheimer's disease. J. Neurochem. *85*, 115–122.

Keembiyehetty, C.N., Krzeslak, A., Love, D.C., and Hanover, J.A. (2011). A lipid-droplet-targeted O-GlcNAcase isoform is a key regulator of the proteasome. J. Cell Sci. *124*, 2851–2860.

Keller, J.N., Hanni, K.B., and Markesbery, W.R. (2000). Impaired proteasome function in Alzheimer's disease. J. Neurochem. *75*, 436–439.

Kondo, T., Imamura, K., Funayama, M., Tsukita, K., Miyake, M., Ohta, A., Woltjen, K., Nakagawa, M., Asada, T., Arai, T., et al. (2017). iPSC-based compound screening and in vitro trials identify a synergistic anti-amyloid β combination for Alzheimer's disease. Cell Rep. *21*, 2304–2312.

Lee, H.T., Sliskovic, D.R., Picard, J.A., Roth, B.D., Wierenga, W., Hicks, J.L., Bousley, R.F., Hamelehle, K.L., Homan, R., Speyer, C., et al. (1996). Inhibitors of acyl-CoA: cholesterol O-acyl transferase (ACAT) as hypocholesterolemic agents. CI-1011: an acyl sulfamate with unique cholesterol-lowering activity in animals fed noncholesterol-supplemented diets. J. Med. Chem. *39*, 5031–5034.

Lee, M.J., Lee, J.H., and Rubinsztein, D.C. (2013). Tau degradation: the ubiquitin-proteasome system versus the autophagy-lysosome system. Prog. Neurobiol. *105*, 49–59.

Leestemaker, Y., de Jong, A., Witting, K.F., Penning, R., Schuurman, K., Rodenko, B., Zaal, E.A., van de Kooij, B., Laufer, S., Heck, A.J.R., et al. (2017). Proteasome activation by small molecules. Cell Chem. Biol. *24*, 725–736.

Liu, C.-C., Liu, C.-C., Kanekiyo, T., Xu, H., and Bu, G. (2013). Apolipoprotein E and Alzheimer disease: risk, mechanisms and therapy. Nat. Rev. Neurol. *9*, 106–118.

Lokireddy, S., Kukushkin, N.V., and Goldberg, A.L. (2015). cAMP-induced phosphorylation of 26S proteasomes on Rpn6/PSMD11 enhances their activity and the degradation of misfolded proteins. Proc. Natl. Acad. Sci. USA *112*, E7176–E7185.

Luna-Muñoz, J., Chávez-Macías, L., García-Sierra, F., and Mena, R. (2007). Earliest stages of tau conformational changes are related to the appearance of a sequence of specific phospho-dependent tau epitopes in Alzheimer's disease. J. Alzheimers Dis. *12*, 365–375.

Mast, N., Anderson, K.W., Johnson, K.M., Phan, T.T.N., Guengerich, F.P., and Pikuleva, I.A. (2017a). *In vitro* cytochrome P450 46A1 (CYP46A1) activation by neuroactive compounds. J. Biol. Chem. *292*, 12934–12946.

Mast, N., Saadane, A., Valencia-Olvera, A., Constans, J., Maxfield, E., Arakawa, H., Li, Y., Landreth, G., and Pikuleva, I.A. (2017b). Cholesterol-metabolizing enzyme cytochrome P450 46A1 as a pharmacologic target for Alzheimer's disease. Neuropharmacology *123*, 465–476.

Merrick, S.E., Demoise, D.C., and Lee, V.M. (1996). Site-specific dephosphorylation of tau protein at Ser202/Thr205 in response to microtubule depolymerization in cultured human neurons involves protein phosphatase 2A. J. Biol. Chem. *271*, 5589–5594.

Michikawa, M., Fan, Q.W., Isobe, I., and Yanagisawa, K. (2000). Apolipoprotein E exhibits isoform-specific promotion of lipid efflux from astrocytes and neurons in culture. J. Neurochem. *74*, 1008–1016.

Minagawa, H., Gong, J.-S., Jung, C.-G., Watanabe, A., Lund-Katz, S., Phillips, M.C., Saito, H., and Michikawa, M. (2009). Mechanism underlying apolipoprotein E (ApoE) isoform-dependent lipid efflux from neural cells in culture. J. Neurosci. Res. *87*, 2498–2508.

Moore, S., Evans, L.D.B., Andersson, T., Portelius, E., Smith, J., Dias, T.B., Saurat, N., McGlade, A., Kirwan, P., Blennow, K., et al. (2015). APP metabolism regulates tau proteostasis in human cerebral cortex neurons. Cell Rep. *11*, 689–696.

Moutinho, M., Nunes, M.J., and Rodrigues, E. (2016). Cholesterol 24-hydroxylase: brain cholesterol metabolism and beyond. Biochim. Biophys. Acta *1861* (12 Pt A), 1911–1920.

Müller, C., Binder, U., Bracher, F., and Giera, M. (2017). Antifungal drug testing by combining minimal inhibitory concentration testing with target identification by gas chromatography-mass spectrometry. Nat. Protoc. *12*, 947–963.

Muratore, C.R., Rice, H.C., Srikanth, P., Callahan, D.G., Shin, T., Benjamin, L.N.P., Walsh, D.M., Selkoe, D.J., and Young-Pearse, T.L. (2014). The familial Alzheimer's disease APPV717I mutation alters APP processing and Tau expression in iPSC-derived neurons. Hum. Mol. Genet. *23*, 3523–3536.

Myeku, N., Clelland, C.L., Emrani, S., Kukushkin, N.V., Yu, W.H., Goldberg, A.L., and Duff, K.E. (2016). Tau-driven 26S proteasome impairment and cognitive dysfunction can be prevented early in disease by activating cAMP-PKA signaling. Nat. Med. *22*, 46–53.

Naldini, L., Blömer, U., Gallay, P., Ory, D., Mulligan, R., Gage, F.H., Verma, I.M., and Trono, D. (1996). In vivo gene delivery and stable transduction of nondividing cells by a lentiviral vector. Science *272*, 263–267.

Ochalek, A., Mihalik, B., Avci, H.X., Chandrasekaran, A., Téglási, A., Bock, I., Giudice, M.L., Táncos, Z., Molnár, K., László, L., et al. (2017). Neurons derived from sporadic Alzheimer's disease iPSCs reveal elevated TAU hyperphosphorylation, increased amyloid levels, and GSK3B activation. Alzheimers Res. Ther. *9*, 90.

Pani, A., Dessì, S., Diaz, G., La Colla, P., Abete, C., Mulas, C., Angius, F., Cannas, M.D., Orru, C.D., Cocco, P.L., et al. (2009). Altered cholesterol ester cycle in skin fibroblasts from patients with Alzheimer's disease. J. Alzheimers Dis. *18*, 829–841.

Parrales, A., Ranjan, A., Iyer, S.V., Padhye, S., Weir, S.J., Roy, A., and Iwakuma, T. (2016). DNAJA1 controls the fate of misfolded mutant p53 through the mevalonate pathway. Nat. Cell Biol. *18*, 1233–1243.

Puglielli, L., Konopka, G., Pack-Chung, E., Ingano, L.A., Berezovska, O., Hyman, B.T., Chang, T.Y., Tanzi, R.E., and Kovacs, D.M. (2001). Acyl-coenzyme A: cholesterol acyltransferase modulates the generation of the amyloid beta-peptide. Nat. Cell Biol. *3*, 905–912.

Puglielli, L., Tanzi, R.E., and Kovacs, D.M. (2003). Alzheimer's disease: the cholesterol connection. Nat. Neurosci. *6*, 345–351.

Ramachandran, K.V., and Margolis, S.S. (2017). A mammalian nervous-system-specific plasma membrane proteasome complex that modulates neuronal function. Nat. Struct. Mol. Biol. *24*, 419–430.

Riekse, R.G., Li, G., Petrie, E.C., Leverenz, J.B., Vavrek, D., Vuletic, S., Albers, J.J., Montine, T.J., Lee, V.M.-Y., Lee, M., et al. (2006). Effect of statins on Alzheimer's disease biomarkers in cerebrospinal fluid. J. Alzheimers Dis. *10*, 399–406.

Sharpe, L.J., Cook, E.C.L., Zelcer, N., and Brown, A.J. (2014). The UPS and downs of cholesterol homeostasis. Trends Biochem. Sci. *39*, 527–535.

Shepardson, N.E., Shankar, G.M., and Selkoe, D.J. (2011a). Cholesterol level and statin use in Alzheimer disease: II. Review of human trials and recommendations. Arch. Neurol. *68*, 1385–1392.

Shepardson, N.E., Shankar, G.M., and Selkoe, D.J. (2011b). Cholesterol level and statin use in Alzheimer disease: I. Review of epidemiological and preclinical studies. Arch. Neurol. *68*, 1239–1244.

Shi, Y., Kirwan, P., Smith, J., MacLean, G., Orkin, S.H., and Livesey, F.J. (2012). A human stem cell model of early Alzheimer's disease pathology in Down syndrome. Sci. Transl. Med. *4*, 124ra29.

Shi, Y., Inoue, H., Wu, J.C., and Yamanaka, S. (2017). Induced pluripotent stem cell technology: a decade of progress. Nat. Rev. Drug Discov. *16*, 115–130.

Shibuya, Y., Niu, Z., Bryleva, E.Y., Harris, B.T., Murphy, S.R., Kheirollah, A., Bowen, Z.D., Chang, C.C.Y., and Chang, T.-Y. (2015). Acyl-coenzyme A:cholesterol acyltransferase 1 blockage enhances autophagy in the neurons of triple transgenic Alzheimer's disease mouse and reduces human P301L-tau content at the presymptomatic stage. Neurobiol. Aging *36*, 2248–2259.

Small, S.A., and Duff, K. (2008). Linking Abeta and tau in late-onset Alzheimer's disease: a dual pathway hypothesis. Neuron *60*, 534–542.

Tajima, Y., Ishikawa, M., Maekawa, K., Murayama, M., Senoo, Y., Nishimaki-Mogami, T., Nakanishi, H., Ikeda, K., Arita, M., Taguchi, R., et al. (2013). Lipidomic analysis of brain tissues and plasma in a mouse model expressing mutated human amyloid precursor protein/tau for Alzheimer's disease. Lipids Health Dis. *12*, 68.

Wang, C., Najm, R., Xu, Q., Jeong, D.-E., Walker, D., Balestra, M.E., Yoon, S.Y., Yuan, H., Li, G., Miller, Z.A., et al. (2018). Gain of toxic apolipoprotein

E4 effects in human iPSC-derived neurons is ameliorated by a small-molecule structure corrector. Nat. Med. *24*, 647–657.

Xie, H., Litersky, J.M., Hartigan, J.A., Jope, R.S., and Johnson, G.V. (1998). The interrelationship between selective tau phosphorylation and microtubule association. Brain Res. *798*, 173–183.

Yang, D.-S., Stavrides, P., Saito, M., Kumar, A., Rodriguez-Navarro, J.A., Pawlik, M., Huo, C., Walkley, S.U., Saito, M., Cuervo, A.M., and Nixon, R.A. (2014). Defective macroautophagic turnover of brain lipids in the TgCRND8 Alzheimer mouse model: prevention by correcting lysosomal proteolytic deficits. Brain *137*, 3300–3318.

Young, J.E., Boulanger-Weill, J., Williams, D.A., Woodruff, G., Buen, F., Revilla, A.C., Herrera, C., Israel, M.A., Yuan, S.H., Edland, S.D., and Goldstein, L.S.B. (2015). Elucidating molecular phenotypes caused by the SORL1 Alzheimer's disease genetic risk factor using human induced pluripotent stem cells. Cell Stem Cell *16*, 373–385.

Young, J.E., Fong, L.K., Frankowski, H., Petsko, G.A., Small, S.A., and Goldstein, L.S.B. (2018). Stabilizing the retromer complex in a human stem cell model of Alzheimer's disease reduces TAU phosphorylation independently of amyloid precursor protein. Stem Cell Reports *10*, 1046–1058.

Yuan, S.H., Martin, J., Elia, J., Flippin, J., Paramban, R.I., Hefferan, M.P., Vidal, J.G., Mu, Y., Killian, R.L., Israel, M.A., et al. (2011). Cell-surface marker signatures for the isolation of neural stem cells, glia and neurons derived from human pluripotent stem cells. PLoS ONE *6*, e17540.

Zissimopoulos, J.M., Barthold, D., Brinton, R.D., and Joyce, G. (2017). Sex and race differences in the association between statin use and the incidence of Alzheimer disease. JAMA Neurol. *74*, 225–232.

STAR★METHODS

KEY RESOURCES TABLE

REAGENT or RESOURCE	SOURCE	IDENTIFIER
Antibodies		
anti-total Tau clone 7 (IF 1:100, WB 1:1000)	EMD Millipore	MAB2239 RRID:AB_1587549
anti-pS202/T205 Tau (IF 1:50, WB 1:500)	Peter Davies	CP13 RRID:AB_2314223
anti-pS396/S404 Tau (IF 1:50, WB 1:500)	Peter Davies	PHF1 RRID:AB_2315150
anti-pThr231 conformational (IF 1:50)	Peter Davies	TG3 RRID:AB_2716726
anti-MAP2 (IF 1:1000)	Abcam	ab5392 RRID:AB_2138153
anti-neurofilament 131/132 (IF 1:5000)	Covance	SMI31 RRID:AB_2314901
anti-Actin clone C4 (WB 1:50,000)	EMD Millipore	MAB1501 RRID:AB_2223041
anti-APLP2 (WB 1:1000)	Calbiochem/Millipore	171617 RRID:AB_565357
anti-APP A4 clone 22C11 (WB 1:1000)	EMD Millipore	MAB348 RRID:AB_94882
anti-APP C-Terminal (WB 1:500)	EMD Millipore	171610 RRID:AB_211444
anti-total Tau (WB 1:1000)	Sigma	T6402 RRID:AB_261728
anti-histone 3 clone (WB 1:1000)	Upstate/Millipore	06-755 RRID:AB_11211742
anti-LC3b (WB 1:1000)	Novus biologicals	NB600-1384 RRID:AB_669581
anti-ubiquitin (WB 1:250)	EMD Millipore	MAB1510 RRID:AB_2180556
anti-Phospho-4E-BP1 (Thr37/46) (WB 1:1000)	Cell Signaling	Antibody #9459 RRID:AB_330985
anti-p70 S6 Kinase Antibody (WB 1:1000)	Cell Signaling	Antibody #9202 RRID:AB_331676
anti-4E-BP1 Antibody (WB 1:1000)	Cell Signaling	Antibody #9452 RRID:AB_331692
anti-Phospho-p70 S6 Kinase (Thr389) (WB 1:1000)	Cell Signaling	108D2, Antibody RRID:AB_2269803
Anti-PSMC2 (D5T1T)) (WB 1:1000)	Cell Signaling	#14395 RRID:AB_2752224
anti-PSMB5 (WB 1:1000)	Enzo Lifesciences	Cat# BML-PW8895 RRID: AB_10540901
anti-GAPDH (WB 1:1000)	Life Technologies	AM4300 RRID: AB_2536381
anti-Nestin Clone 25/NESTIN (RUO) (1:500)	BD	611658 RRID:AB_399176
anti-S100b (1:500)	Proteintech	15146-1-AP RRID:AB_2254244
anti-Vamp2 (aka synaptobrevin 2) (1:1000)	synaptic systems	104 211 RRID:AB_887811
anti- Syntaxin 1 (1:5000)	Gift from the Thomas Sudhof lab	polyclonal #I379
anti-Synaptotagmin (1:2000	Gift from the Thomas Sudhof lab	polyclonal #W855
Tra-181-647	BD	560124 RRID:AB_1645449
CD184-APC (FC 1:10)	BD	555976 RRID:AB_398616
CD44-PE (FC 1:10)	BD	555479 RRID:AB_395871
CD24-PECy7 (FC 1:40)	BD	561646 RRID:AB_10892826
CD271-PE	BD	557196 RRID:AB_396599
Chemicals, Peptides, and Recombinant Proteins		
T0901317	Sigma	T2320
GW501516	Enzo life sciences	89150-762
Rosiglitazone	Sigma	R2408
Atorvastatin calcium salt	Sigma	PZ001
Simvastatin	Sigma	S6196
Rosuvastatin	Sigma	SML1264
Fluvastatin	Sigma	SML0038
Mevastatin	Tocris	1526
Lovastatin	Sigma	PHR1285
Mevalonolactone (Mevalonic Acid)	Sigma	M4777

(*Continued on next page*)

Continued

REAGENT or RESOURCE	SOURCE	IDENTIFIER
(R)-Mevalonic acid 5-pyrophosphoate tetralithium salt	Sigma	77631
Mevalonic acid 5-phosphate thrilithium salt hydrate	Sigma	79849
Methyl-β-cyclodextrin	Sigma	C4555
YM-53601	Cayman Chemicals	18113
AY 9944 dihydrochloride	Tocris	1639
FTI-227 trifluoroacetate salt	Sigma	F9803
GGTI-298 trifluoracetate salt hydrate	Sigma	G5169
Fatostatin hydrobromide	Sigma	F8932
24(S)-hydroxycholesterol	Cayman Chemicals	10009931
chloroquine diphosphate salt	Sigma	C6628
MG-132 (InSolution)	Calbiochem	37391
Cholestane	Sigma-Aldrich	C8003
N-Methyl-*N*-(trimethylsilyl)trifluoroacetamide (MSTFA)	ThermoFisher	TS48910
dry pyridine	Sigma-Aldrich	270970
sodium hydroxide solution 10M (BioUltra)	Sigma Aldrich	72068
Ri, Y-27632 dihydrochloride	Abcam	ab120129
SB431542	Stemgent	4-0010
Noggin	R&D	3344-NG
Proteasome activity probe 1	Berkers et al., 2007; de Jong et al., 2012	N/A
SCR7	Excess Bioscience	M60082-2
L-755,507	Sigma	SML1362
K-604	Sigma	SML1837
**Critical Commercial Assays**		
Phospho(Thr231)/Total Tau Kit	MSD	K15121D-3
V-PLEX Ab Peptide Panel 1 (6E10) Kit (25 Plate)	MSD	K15200E-4
24(S)-Hydroxycholesterol ELISA kit	Enzo	ADI-900-210-0001
CellTiter-Glo® Luminescent Cell Viability Assay	Promega	G7571
CellTiter 96® AQueous One Solution Cell Proliferation Assay	Promega	G3580
Pierce LDH Cytotoxicity Assay Kit	Thermo Fisher Scientific	88954
Amplex Red Cholesterol Assay Kit	ThermoFisher Scientific	A12216
Lipidyzer	Sciex	N/A
**Experimental Models: Cell Lines**		
CV (NPC)	Gore et al., 2011	RRID:CVCL_1N86
151 (CV WT) (iPSC, NPC)	Fong et al., 2018	RRID: CVCL_UI49
IB6 (APP^{null} in CV background) (iPSC, NPC)	Fong et al., 2018	RRID:CVCL_UI22
B10 (CV Wt) (iPSC, NPC)	This Paper	RRID:CVCL_VR50
B11 (CV Wt) (iPSC, NPC)	This Paper	RRID:CVCL_VR51
3d9 (CV APP E693A) (iPSC, NPC)	This Paper	RRID:CVCL_VR53
2b2 (CV APP E693A) (iPSC, NPC)	This Paper	RRID:CVCL_VR52
D12 (CV APP E693A+F691A) (iPSC, NPC)	This Paper	RRID:CVCL_VR54
APP^{dp}1.2 (iPSC, NPC)	Israel et al., 2012	RRID:CVCL_EJ97
APP^{dp} 1.6 (iPSC, NPC)	Israel et al., 2012	RRID:CVCL_EJ96
APP^{dp} 2.1 (iPSC, NPC)	Israel et al., 2012	RRID:CVCL_EJ99
APP 1KO (APP^{null} in APP^{dp}1.2 background) (iPSC, NPC)	This Paper	RRID: CVCL_UI23
NDC1 (M) NPC	Israel et al., 2012; Young et al. 2015	RRID:CVCL_EJ84
NDC2 (M) NPC	Israel et al., 2012; Young et al., 2015	RRID:CVCL_EJ87

(*Continued on next page*)

Continued

REAGENT or RESOURCE	SOURCE	IDENTIFIER
NDC3 (M) NPC	Young et al., 2015	RRID: CVCL_UB88
NDC4 (M) NPC	Young et al., 2015	RRID: CVCL_UB89
NDC5 (M) NPC	Young et al., 2015	RRID: CVCL_UB90
SAD1 (F) NPC	Israel et al., 2012; Young et al., 2015	RRID: CVCL_EJ90
SAD2 (M) NPC	Israel et al., 2012; Young et al., 2015	RRID: CVCL_EJ93
SAD3 (M) NPC	Young et. al. 2015	RRID: CVCL_UB91
SAD4 (F) NPC	Young et. al. 2015	RRID: CVCL_UB92
SAD5 (F) NPC	Young et. al. 2015	RRID: CVCL_UB93
SAD6 (M) NPC	Young et. al. 2015	RRID: CVCL_UB94
SAD7 (M) NPC	Young et. al. 2015	RRID: CVCL_UB95
Experimental Models: Organisms/Strains		
C57BL/6J Mice	Jackson Laboratory	000664
Oligonucleotides		
Repair Oligos used to make CRISPR mutant lines See Table S6	This Paper	N/A
Primers for amplification of APP Cholesterol Binding region See Table S6	This Paper	N/A
APP Cholesterol binding region genomic DNA Sequencing Primer See Table S6	This Paper	N/A
qPCR Primers See Table S6	This Paper	N/A
Recombinant DNA		
G7A: TOPO guide RNA plasmid for Generating cholesterol-mutant lines with target sequence: 5′GTGTTCTTTGCAGAAGATGTGGG3′	This Paper	N/A
Guide RNA plasmid for generating APP^{null} line in APP^{dp}-patient background with target sequence: 5′GGAGATCTCTGAAGTGAAGATGG3′	This Paper	N/A
CMV::Cas9-2A-eGFP	Sigma-Aldrich	CAS9GFPP-1EA
CYP46A1-pDESTlentiFGA1.0.	This Paper	N/A
GFP-only plasmid-pDESTlentiFGA1.0. (pSyn(pr)EGFPLL3.7)	This Paper	N/A

CONTACT FOR REAGENT AND RESOURCE SHARING

Further information and requests for resources and reagents should be directed to and will be fulfilled by the Lead Contact, Larry Goldstein (lgoldstein@ucsd.edu).

All cell line requests will require a material transfer agreement (MTA).

EXPERIMENTAL MODEL AND SUBJECT DETAILS

Cultured Mouse Cortical Neurons

Primary cortical neurons were cultured from postnatal day zero C57BL/6J pups. Briefly, pups were decapitated into Hanks' medium without Ca^{2+} and Mg^{2+}, and cortices were dissected in Neurobasal-A medium supplemented with 10 mm HEPES. After dissection, cortices were trypsinized for 25 min at 37°C and dissociated. Neurons were plated in Neurobasal-A with B27 supplement and drug-treatment was performed after 2 weeks.

Human iPSC Derived Cell lines

Cell lines

Novel NPC lines were generated from previously established iPSC lines. The APP^{dp} AD-patient NPC lines used in this study are; APP^{dp}1-6 (derived from iPSC-line APP^{dp}1.1 in Israel et al. [2012]), APP^{dp}1-2 (derived from iPSC-line APP^{dp}1.2 in Israel et al. [2012]) and APP^{dp}2-1 (derived from iPSC-line APP^{dp}2.3 in Israel et al. [2012]). See Israel et al. (2012) for generation and

characterization of these APP^{dp} iPSC lines and patient details. SAD and NDC NPC lines used are CV4a, SAD1, SAD2, SAD5, SAD5, SAD7, NDC1, NDC2, NDC4 and NDC5 described in Young et al. (2015);

To generate isogenic APP knockout iPSCs in an APP^{dp} patient 1 background, iPSC-line APP^{dp}1-2 was gene edited to generate the iPSC and NPC-line APP^{dp}1KO. To generate isogenic APP knockout lines in wild-type (Craig Venter, CV) background, iPSC-line CVB (Gore et al., 2011) was gene edited to generate IPSC and NPC-line IB6 (CV APP^{null}) and 151 (CV wt) (Fong et al., 2018). To generate isogenic Δcholesterol APP mutants the iPSC-line CVB (Gore et al., 2011) was gene edited to generate iPSC and NPC-lines B10 (wt), B11 (wt), 3D9 (homozygous E693A), 2B2b (homozygous E693A) and D12 (homozygous F691A+E693A).

iPSC genome editing

Generation of isogenic APP knockout lines in a wild-type (CV iPSC) background, IB6 (APP knockout) and 151 (wt) was described previously (Fong et al., 2018). For gene-editing, iPSCs were pretreated with 10uM Rock inhibitor (Ri, Y-27632 dihydrochloride, Abcam) 2 hours prior to nucleofection. iPSC's were dissociated with Accutase and filtered through 100uM filter and spun down at 1000rpm for 5 minutes. Two million cells were nucleofected according to manufacturer instructions using the Amaxa Human Stem Cell Nucleofector Kit 1 (Lonza).

To generate an isogenic APP^{null} line in an APP^{dp}-patient background, the iPSC-line APP^{dp}1.2 was nucleofected with 6 μg CMV::Cas9-2A-eGFP vector and 3 μg U6::gRNA vector. To target the CRISPR/Cas9 we used the gRNA target sequence: 5′-GGA GATCTCTGAAGTGAAGATGG-3′ (see Table S6 for all oligos used). Nucleofected iPSCs were replated into a 6 well of MEF feeder cells in the presence of Ri and allowed to recover for 72 hours, 1×10^4 GFP$^+$ iPSCs were FACS sorted (FACS Aria IIu, BD Biosciences) and (sparsely) plated on 10 cm MEF-feeder plates in the presence of Ri. After 7 days, individual colonies were manually picked and cultured in individual wells of a 96-well plate. Cells were split and DNA was isolated from individuals wells (quickextract kit, Epicenter), PCR amplified using the primers (APPex16-F: CCC GTA AGC CAA GCC AAC AT, APPex16-R: CAT GCA CGA ACT TTG CTG CC) and sequenced using the primer AGGCAGCAGAAGCCTT and aligned against the APP wild-type sequence. Amplified PCR-fragments from non-wild-type colonies were selected, cloned using the Zero Blunt TOPO PCR Cloning Kit (Invitrogen) and sequenced to analyze the (edited) genomic DNA sequence on both alleles. One confirmed APP^{null} line was expanded and differentiated to neural progenitor cells and characterized as detailed in Figure S3. To generate isogenic APPΔcholesterol lines in a CV background, the CV iPSC-line was nucleofected with 3.4 μg of a CMV::Cas9-2A-eGFP vector, 1.6 μg of a TOPO blunt II::gRNA vector, and 50nmol (4ng) repair single-stranded oligonucleotide. To target the CRISPR/Cas9 we used the gRNA target sequence: 5′-GTGTTCTTTGCAGAA GATGTGGG-3′. Repair oligo's to generate E693A and F691+E693A respectively were 5′-Tattgcatttagaaattaaaattctttttcttaatt tgttttcaaggtgttctttgcagccgatgtgggttcaaacaaaggtgcaatcattggactcatggtgggcggtgttgtcatagc-3′ and E693A+F691A Repair Oligo Sequence: 5′-ttatattgcatttagaaattaaaattctttttcttaatttgttttcaaggtgttcgctgcagccgatgtgggttcaaacaaaggtgcaatcattggactcatggtggg cggtgttgtcat-3′ respectively.

Nucleofected iPSCs were re-plated into a 6 well of MEF feeder cells in the presence of Ri and and 1 μM SCR7 (Excess Bioscience, M60082-2) and/or 5 μM L-755,507 (Sigma, SML1362).

Cells were then allowed to recover for 48 hours after which, live, GFP+ iPSCs were FACS sorted (FACS Aria IIu, BD Biosciences) and plated at 10,000 cells per 10 cm MEF-feeder plates in the presence of Ri. After 7-14 days, individual colonies were manually picked and cultured in individual wells of 96-well plates. 96 well plates were split and DNA was isolated from each well of one of the plates (quickextract kit, Epicenter). The region of interest was PCR amplified using Forward Primer: 5′-CTTCCTCGAACTGGG GAAGC-3′ and Reverse Primer: 5′-TCACGGTAAGTTGCAATGAATGA-3. Coincidentally, mutation of the wild-type sequence to the sequence corresponding to the E693A mutation generates a novel BbvI restriction motif, whereas introduction of the F691A mutation creates a PstI restriction enzyme motif. Therefore, initial screening was performed by performing a restriction digest on the PCR-amplified fragment by incubating the PCR amplicon with BbvI (for E693A) or PstI (F691A+E693A). These fragments were run on a DNA-gel and samples in which full restriction had occurred (homozygous for the desired mutation) were confirmed by sequencing of the amplicon using the primer: 5′-CCAACCAGTTGGGCAGAGAA-3′ Once confirmed, two individual IPSC lines containing the homozygous E693A mutation (3D9, 2B2), one line containing the homozygous F691A+E693A mutation (D12) and two unedited controls lines (B10, B11) that underwent the same clonal expansion process were expanded and differentiated to neural progenitor cells.

Additional Cell Line Information

The Craig Venter Cell line (CV) as well as all gene edited isogenic lines generated from this line are male. The CV line was previously reported and characterized in Gore et al. (2011). Cell lines generated in this background include the isogenic knockout lines IB6 (APP knockout) and 151 (wt), and the Δcholesterol mutant lines 3D9 and 2B2 (APP E693A), D12 (F691A+E693A), and B10 and B11 (WT controls). All APP^{dp}1 (patient 1) derived lines are male, all APP^{dp}2 (patient 2) derived lines are female (Israel et al., 2012).

The lines NDC1, NDC2, NDC3, NDC4, NDC5, SAD2, SAD3, SAD6, and SAD7 are male. The lines SAD1, SAD4, and SAD5 are female. The NDC and SAD lines were previously reported and characterized in Israel et al. (2012) and Young et al. (2015). Analyses of the influence of sex was not evaluated in this study. Rather the effect of drug-treatment on individual cell-lines before and after treatment was compared or between isogenic gene-edited lines.

Copy number determination

200 ng of DNA was hybridized to Illumina HumanCore arrays (Illumina), and stained per Illumina's standard protocol. Copy Number Variation (CNV) calling was carried out in Nexus CN (version 7.5) and manually inspected, visualizing the B-allele frequencies (proportion of A and B alleles at each genotype) and log R ratios (ratio of observed to expected intensities) for each sample, as described in D'Antonio et al. (2017).

Generation of neural progenitor cells
NPC's were generated from iPSCs as described previously (Yuan et al., 2011). In short, 2 × 10^5 FACS-purified iPSC TRA1-81$^+$ cells were seeded onto two 10 cm plates that were seeded the previous day with 5 × 10^5 PA6 cells and were cultured in PA6 differentiation media (450ml Glasgow DMEM, 50ml KO Serum Replacer, 5ml sodium pyruvate, 5ml Nonessential Amino Acids) + 10um SB431542+ 0.5ug/ml Noggin. After 6 days in culture, media was changed to PA6 differentiation media without SB431542 and Noggin. At day 11, cells were dissociated with Accutase and ~5 × 10^5 CD184$^+$CD24$^+$CD44$^-$CD271$^-$ NPCs were FACS-purified and plated onto poly-L-ornithine/laminin-coated plates and cultured with NPCbase + 20ng/ml bFGF (Millipore). Of note, while iPSC are grown in lipid containing media, the derived NPC after sorting (and for neuronal differentiation) are always grown in medium that does not contain an exogenous source of lipids.

Cell culture, generation of neurons and astrocytes
iPSCs were cultured on a MEF feeder layer in HUES medium (KO DMEM, knockout serum, plasmanate, pen-strep, non-essential amino acids, glutamax and β-mercaptoethanol) + 20ng/ml bFGF (Millipore) as described previously (Israel et al., 2012) on a MEF feeder layer and passaged with Accutase (Innovative Cell Technologies). NPCs were cultured on poly-L-ornithine (0.02 mg/ml) and laminin (5 ug/ml) (Sigma)-coated plates in DMEM:F12 + Glutamax, 0.5x N2, 0.5x B27, Pen/Strep (all Life Technologies), and 20 ng/ml FGF, and passaged with Accutase. For neuronal differentiation, NPCs were expanded to confluence, after which FGF was withdrawn from the culture medium and the medium was changed twice weekly. For all experiments, unless stated otherwise, neurons after 3 weeks of differentiation were replated into 96 wells (2 × 10^5 living cells/well) for 2 weeks in 200 ul NPC base + BDNF/GDNF/cAMP. After 2 weeks media was removed and fresh media (200 ul NPC base + BDNF/GDNF/cAMP) containing the tested compounds was added. At indicated time points, the conditioned culture media was harvested from cells and cells were lysed in 70 μL MSD lysis buffer (MSD) with protease (Calbiochem) and phosphatase inhibitors (Halt). For Figures 4E and 4F (cholesterol mutants) 3-week differentiated neurons were plated for 2 weeks and treated with DMSO and Atorvastatin respectively for 2 days. On day 3 a full media change was performed (containing DMSO or Atorvastatin) and 24 hours later the media was collected for Aβ measurements. For experiments in Figure 5C, MG132 was added fresh to the media every day. For astrocytic differentiation, a confluent 10 cm plate of NPCs was scraped in NPC medium, and transferred to 3 wells of a 6 well plate placed on a 90 RPM orbital shaker in the incubator to promote neurosphere formation. 24 hours later, 5 μM RI was supplemented to the medium for 48 hours. After 48 hours the neurospheres were grown in NPC medium (withouth FGF) that was replaced every 2 to 3 days. One week after scraping, media was changed to Astrocyte Growth Medium (AGM) containing 3% FBS, ascorbic acid, rhEGF, GA-1000, insulin, and L-glutamine (Lonza) and cell were cultured for an additional two weeks. After two weeks, the neurospheres were plated on a poly-L-ornithine/laminin-coated 10 cm plate. After seven days the astrocytes emerging from the neurospheres were passaged with Accutase, cultured in AGM, and maintained with the neurospheres.

Neuron FACS sort
For Aβ measurements in purified Δcholesterol mutant neurons (Figure 4D) and tau measurements in the APPdp and APP^{null} cells (Figure S3I), neurons were purified to compare steady state Aβ measurement within different lines. After three weeks of neuronal differentiation in NPCbase media without FGF; CD184$^-$, CD44$^-$, CD24$^+$ (antibodies from BD Bioscience) neurons were purified by FACS (BD Biosciences) and were plated onto poly-L-ornithine/laminin-coated coated plates. Sorted neurons were cultured in NPCbase media + 0.5mM dbCAMP, 20ng/mL BDNF, and 20ng/mL GDNF for 5-7 days before experiments.

METHOD DETAILS

Reagents
Compounds used are T0901317 (Sigma, T2320) GW501516 (Enzo life sciences, 89150-762), Rosiglitazone (Sigma, R2408), Atorvastatin calcium salt (Sigma, PZ001), Simvastatin (Sigma, S6196), Rosuvastatin (Sigma, SML1264), Fluvastatin (Sigma, SML0038), Mevastatin (Tocris, 1526) Lovastatin (Sigma, PHR1285), Mevalonolactone (Mevalonic Acid) (Sigma, M4777), (R)-Mevalonic acid 5-pyrophosphoate tetralithium salt (Sigma, 77631), Mevalonic acid 5-phosphate thrilithium salt hydrate (Sigma, 79849), Methyl-β-cyclodextrin (Sigma, C4555), YM-53601 (Cayman Chemicals, 18113), AY 9944 dihydrochloride (Tocris), FTI-227 trifluoroacetate salt (Sigma, F9803), GGTI-298 trifluoracetate salt hydrate (Sigma, G5169), Fatostatin hydrobromide (Sigma, F8932), 24(S)-hydroxycholesterol (Cayman Chemicals, 10009931), K-604 (Sigma-Aldrich, SML1837), chloroquine diphosphate salt (Sigma, C6628), MG-132 (InSolution, Calbiochem, 37391), Cholestane (Sigma-Aldrich C8003) *N*-Methyl-*N*-(trimethylsilyl)trifluoroacetamide (MSTFA, ThermoFisher TS48910), 1 μL of dry pyridine (Sigma-Aldrich, 270970) and sodium hydroxide solution 10M (BioUltra, Sigma Aldrich, 72068).

The compound collection for the screen consisted of 1684 FDA and EMA approved drugs, as well as drugs that had been tested in clinical trials. This collection was assembled from multiple commercially available libraries, including the Prestwick Chemical Library (Prestwick Chemical), Microsource US and International Drug Collections (Microsource Discovery Systems, Inc.), and NIH clinical collection libraries (http://www.nihclinicalcollection.com). An overview of all compounds in the screen can be found in Table S1.

Phenotypic high-throughput screen
After thawing, APP^{dp}1-6 NPC's (passage 13) were expanded to confluence on 10cm dishes and differentiated at passage 16 (p16) by withdrawal of bFGF. After 3 weeks, differentiated NPC's were washed with PBS and dissociated using Accutase:Accumax

(Innovative Cell Technologies) (1:1). Cells were resuspended in sort buffer (NPCbase Media+ 1% FBS (Mediatech 35-011-CV)+2.5mM EDTA) and filtered through a sterile cell strainer (100um, Fisher scientific, Cat no. 22363549) to remove clumps. Cells were spun down (5 min at 1000RPM) and resuspended in NPCbase + 20 ng/μl BDNF, 20 ng/μl GDNF (Peprotech) and 0.5 mM dbcAMP (Sigma).

Cells were plated in 384 well poly-L-ornithine/laminin-coated plates at 5×10^4 live cells/well in 50 μL and were allowed to further mature for 2 weeks. After 1 week, 25 μL media was aspirated and replaced with fresh media (NPC base + BDNF/GDNF/cAMP). 1 week later (in total 2 weeks on the 384 well plate) 15 μL of media was aspirated (leaving 35 μL in the well) and 35 μL media + 2x compound (NPC base + 2x BDNF/GDNF/cAMP and 10 μM of compound) was added to generate a final concentration of 5 μM compound/well or vehicle (DMSO, 16 wells per 384 well plate) in duplicate. After five days of treatment, media was aspirated and cells were lysed in 70 μL Mesoscale discovery lysis buffer (MSD R60TX-3) with protease (Calbiochem) and phosphatase inhibitors (Halt) and lysates were stored at −80 awaiting analysis. From the lysates viability was measured using CellTiter-Glo® Luminescent Cell Viability Assay (Promega) according to manufacturer instructions with a modified ratio of lysate to CeLLTiterGlo reagent (1:1) and pThr231/tTau levels were determined using Phospho(Thr231)/Total Tau kit (K15121D, Meso Scale Discovery). For each 384 well plate, Z-scores were determined for each data point, as the number of standard deviations from the mean of the control, vehicle-treated (DMSO) wells. Compounds that decreased pThr231Tau/tTau by $Z < -2$ in one of the duplicates were selected for confirmational screening (repeat of the methods described for the primary screen). Compounds that decreased pThr231Tau/tTau in an additional replicate ($Z < -2$) in the secondary screen were defined as hits.

Aβ, pThr231Tau/total Tau, pSer9GSK3b/total Gsk3b, Phospho-4E-BP1(Thr37/46)/total 4E-BP1 ELISA Measurements

For Aβ measurements, 25 μL of the culture media was run on a V-PLEX Aβ Peptide Panel 1 (6E10) (K150SKE) kit, for pThr231Tau/tTau lysate was run on a Phospho (Thr231)/Total Tau Kit (K15121D), for pSer9GSK3β/total Gsk3β lysate was run on pSer9GSK3β /total Gsk3β Kit (K15109D). For Phospho-4E-BP1(Thr37/46)/total 4E-BP1 lysates were run separately on a Phospho-4E-BP1(Thr37/46) kit (K150KHD) and a total 4E-BP1 kit (K151OLD) and ratio was determined by combining these two measures from the same lysates. All kits from Mesoscale discovery. Measurements were performed on Mesoscale discovery sector imager 600. For Figure 4D (cholesterol mutants) purified neurons were replated after FACS and media was collected after 5 days.

Cell viability

From the lysates viability was measured using CellTiter-Glo® Luminescent Cell Viability Assay (Promega) according to manufacturer instructions with a modified ratio of lysate to CeLLTiterGlo reagent (1:1). CellTiter 96® AQueous One Solution Cell Proliferation Assay (Promega) and Pierce LDH Cytotoxicity Assay Kit (Thermo Fisher Scientific) were performed in 96-well plates in accordance with manufacturer instructions.

Mass spectrometry sample preparation and LC-MS-MS

The frozen cell pellet was resuspended in an equal volume of water and then vortexed to thaw the sample. We added 100 ul of cell suspension to 500μl of 6 Molar Guanidine solution followed by vortexing. The sample was then boiled for 5 minutes followed by 5 minutes cooling at room temperature, this step was repeated 3 times. The proteins were precipitated with addition of methanol followed by vortex and centrifugation at 14000 rpm for 10 minutes. The soluble fraction was removed. The pellet was resuspended in 600ul of 8 M Urea made in 100mM Tris pH 8.0 and by vortexing for 5-10 minutes. TCEP was added to final concentration of 10 mM. The sample was then frozen overnight at −20C. Next day the solution was thawed and vortexed for another 5 minutes until the solution became clear. Chloro-acetamide solution was added to final concentration of 40 mM and vortexed for 5 minutes. Equal volume of 50mM Tris pH 8.0 was added to the sample to reduce the urea concentration to 4 M. Lys C was added in 1:500 ratio of LysC to protein content and incubated at 37C in a rotating incubator for 4-6 hours. Equal volume of 50mM Tris pH 8.0 was added to the sample to reduce the urea concentration to 2 M. Trypsin was added in 1:50 ratio of trypsin to protein content. Next day the solution was acidified using TFA (0.5% TFA final concentration) and vortexed for 5 minutes. The sample was centrifuged at 15, 700 g for 5 min to obtain aqueous and organic phases. The lower aqueous phase was collected and desalted using 100 mg C18-StageTips as described by the manufacturer protocol. The peptide concentration of sample was measured using BCA after resuspension in sample loading buffer and the total of 2 ug is injected for each label free quantification run. Trypsin-digested peptides were analyzed by ultra high pressure liquid chromatography (UPLC) coupled with tandem mass spectroscopy (LC-MS/MS) using nano-spray ionization. The nanospray ionization experiments were performed using a Orbitrap fusion Lumos hybrid mass spectrometer (Thermo) interfaced with nano-scale reversed-phase UPLC (Thermo Dionex UltiMate 3000 RSLC nano System) using a 25 cm, 75-micron ID glass capillary packed with 1.7-μm C18 (130) BEH^{TM} beads (Waters corporation). Peptides were eluted from the C18 column into the mass spectrometer using a linear gradient (5%–80%) of ACN (Acetonitrile) at a flow rate of 375 μl/min for 1h. The buffers used to create the ACN gradient were: Buffer A (98% H_2O, 2% ACN, 0.1% formic acid) and Buffer B (100% ACN, 0.1% formic acid). Mass spectrometer parameters are as follows; an MS1 survey scan using the orbitrap detector (mass range (m/z): 400-1500 (using quadrupole isolation), 120000 resolution setting, spray voltage of 2200 V, Ion transfer tube temperature of 275 C, AGC target of 400000, and maximum injection time of 50 ms) was followed by data dependent scans (top speed for most intense ions, with charge state set to only include +2-5 ions, and 5 s exclusion time, while selecting ions with minimal intensities of 50000 at in which the collision event was carried out in the high energy collision cell (HCD Collision Energy of 30%), and the fragment masses where analyzed in the ion trap mass analyzer (With ion trap scan rate of turbo, first mass m/z was 100, AGC Target 5000 and maximum

injection time of 35ms). Protein identification and label free quantification was carried out using Peaks Studio 8.5 (Bioinformatics solutions Inc.)

Lipid measurements

For the analysis of cholesterol and its precursors by means of gas chromatography mass spectrometry (GC-MS), treated neurons were pelleted by centrifugation and resuspended in 50 µL water. 600 µL MTBE and 160 µL methanol was added and samples were vortexed and shaken at room temperature for 30 minutes. For phase separation 200 µL water was added. Samples were spun at 16.100 g for 5 minutes and the organic (soluble) extract (350 µL) was separated from the pelleted protein. Extraction was repeated by the addition of 100 µL methanol, 100 µL water and 300 µL MTBE. After centrifugation the organic extracts were combined (650 µL) and split into two fresh glass vials (one for the total cholesterol fraction (200µL) and one for free cholesterol fraction (450µL)). Samples were dried under a gentle stream of nitrogen. For free cholesterol analysis, 10 µL of an internal standard solution (IS) containing 10 µg/mL cholestane in MTBE, 50 µL of *N*-Methyl-*N*-(trimethylsilyl)trifluoroacetamide (MSTFA), 1 µL of dry pyridine and 39 µL of MTBE were added to the dried extract. The samples were kept at room temperature for 30 minutes in order to complete derivatization before injection. For total cholesterol analysis, 10 µL IS solution, 400 µL LC-MS grade water and 100 µL 10M NaOH were added and samples were heated to 60°C for one hour. After samples had cooled to room temperature sterols were extracted twice using 750 µL MTBE. The combined organic extracts were dried under a gentle stream of nitrogen and dissolved in 50 µL of MSTFA, 1 µL of dry pyridine and 49 µL of MTBE. Samples were kept at room temperature for 30 minutes before injection. GC-MS analysis was carried out on a ScionTQ GC-MS system (Bruker Daltonics). Helium (99.9990%) was used as carrier gas on an Agilent VF-5ms column (5% phenyl-methyl; 25 m × 0.25 mm internal diameter; 0.25 µm film thickness). The injector was kept at 280°C and 1 µL was injected split-less. The oven program started at 50°C, held for 1 minute, followed by a ramp of 50°C/min to 260°C, continued to ramp with 4°C/min to 310°C, held for 0.3 minutes. Sterols were identified based on their relative retention time and comparison of electron ionization mass spectra (Giera et al., 2007; Müller et al., 2017). For quantification of free and total cholesterol an external calibration line from 0-500 µg/mL cholesterol was constructed. Total ion current chromatograms were integrated and quantified by the use of the external calibration lines. Pelleted protein was resuspended in 2% SDS and quantified according to the manufacturers instructions using the Pierce BCA Protein Assay Kit (cat. no. 23225).

Quantitative cholesterol ester and phospholipid analysis was carried out using a commercial platform (The Lipidyzer, Sciex, Redwood, CA, USA). In brief, treated neurons were pelleted by centrifugation and resuspended in 100 µL water and 100 µL internal standard mixture in methyl-tert.-butyl ether (MTBE). An additional 500 µL MTBE and 160 µL methanol was added and samples were vortexed and shaken at room temperature for 30 minutes. For phase separation 200 µL LC-MS grade water was added and samples were spun for 3 minutes at 16.100 × g. The upper organic layer was transferred to a glass vial. The extraction was repeated after the addition of 300 µL MTBE, 100 µL methanol and 100 µL water. The combined organic extracts were dried under a gentle stream of nitrogen and 250 µL running buffer was added. The analysis was carried out using the commercial flow-injection based quantitative lipidomics platform (The Lipidyzer). For analysis of relative cholesterol ester levels the cumulative concentrations of all cholesterol esters detected was normalized over the cumulative concentration of all sphingomyelins detected.

Microscopy

After differentiation, neurons were directly fixed or replated on 96-well imaging plates at a density of 2 × 10^5 living cells per well. Cells were imaged either 1 week (APPΔcholesterol mutants, supplemental 4b) or 2 weeks + 5 days drug treatment (APP^{dp}1-6 lines, Figure 1C) after replating. Cells were fixed with 4% PFA + 4% sucrose for 15 minutes at room temperature, washed 3 times with PBS, permeabilized with 0.1% Triton X-100, blocked with 0.5% BSA/PBS for 30 minutes and incubated with primary antibodies in 0.5% BSA/PBS for 1 hour at room temperature. Cells were subsequently washed 3 times with PBS, incubated with secondary antibodies in 0.5% BSA/PBS for 45 minutes and washed 3 more times with PBS (with the first wash containing 1:1000 DAPI). After 3 washes 100 µL PBS was added to each well and samples were imaged. The antibodies used for immunofluorescence experiments were anti-total Tau clone 7 (MAB2239, 1:100, EMD Millipore), anti-pS202/T205 Tau (CP13, 1:50 Peter Davies), anti-pS396/S404 Tau (PFH1, 1:50 Peter Davies), anti-pThr231 conformational (TG3, 1:50 Peter Davies), anti-MAP2 ab5392 (1:1000, Abcam) and anti-neurofilament 131/132 (SMI31) (1:5000, Covance). Anti-Nestin (BD biosciences, Clone 25/NESTIN (RUO), 611658)

S100b (Proteintech, 15146-1-AP) Secondary antibodies were Alexa Fluor anti-mouse, anti-rabbit and anti-chick (Invitrogen) and used at 1:200. Images for Figures 1E, 6A, S1F, and S4A were acquired on a Leica TCS SPE confocal microscope, images for Figures S1A, S1B, and S5A were acquired on a Nikon A1 confocal microscope.

RNA expression analysis

For mRNA expression analysis, RNA was isolated using the RNeasy Mini Kit (QIAGEN) and DNase-treated using TURBO DNase (Ambion) for one hour at 37°C. cDNA was generated from RNA primed with oligo(dT) using the SuperScript First-Strand Synthesis System (Invitrogen). We performed RT-qPCR using FastStart SYBR Green (Roche) using an Applied Biosystems 7300 RT-PCR system. Data was analyzed using the delta-Ct method and target genes were normalized to the geometric mean of a housekeeping gene (RPL27). The following primers were used: *HMGCR-F*: CGT GGA ATG GCA ATT TTA GGT CC, *HMGCR-R*: ATT TCA AGC TGA CGT ACC CCT, *LDLR-F*: GTC TTG GCA CTG GAA CTC GT, *LDLR-R*: CTG GAA ATT GCG CTG GAC, PSMB5 FW: GCTACCGGT

GAACCAGCG, PSMB5 RV: CAACTATGACTCCATGGCGGA, PSMC2 FW: TGAGAGTGGGCGTGGATAGA, PSMC2 RV: GTACCGGGTGGACCAAAGAG, *RPL27-F*: AAA CCG CAG TTT CTG GAA GA, *RPL27-R*: TGG ATA TCC CCT TGG ACA AA.

Quantitative western blot

NPCs were differentiated in 6 wells for 3 weeks and treated for 5 days with compound after which cells were lysed in RIPA Lysis Buffer (Millipore) with protease (Calbiochem) and phosphatase inhibitors (Halt). Protein concentrations were determined (Pierce BCA Protein Assay Kit, Thermo Fisher) and normalized. 4x LDS Sample buffer (novex) + β-mercaptoethanol was added to the lysate to generate a final concentration of 1x sample buffer + 5% β-mercaptoethanol. Samples were boiled (100°C) for 5 minutes. Equal amounts of lysates were run on NuPAGE 4%–12% Bis-Tris gels (Invitrogen), transferred to nitrocellulose or PVDF membranes, and blocked for one hour at room temperature using Odyssey Blocking Buffer (LI-COR). Blots were probed overnight at 4°C using the corresponding primary antibodies followed by IRDye secondary antibodies (LI-COR) at 1:5,000 and images were acquired by LICOR Odyssey imager. Bands were quantified using ImageJ software. Western blot images within one panel showing a composition of bands of interest for a particular immunogen are always from the same image (identical blot and exposure), but cropped and grouped together for clarity. Stiches are indicated by vertical lines. Antibodies used were: anti-Actin (1:50,000; EMD Millipore), anti-APLP2 (1:1,000; Calbiochem), anti-APP A4 clone 22C11 (1:1,000; EMD Millipore), anti-APP C-Terminal (1:500; EMD Millipore), anti-pS202/T205 Tau (CP13, 1:500 Peter Davies), anti-pS396/S404 Tau (PHF1, 1:500 Peter Davies), anti-total Tau T6402 (1:1000, Sigma), anti-total Tau clone 7 (MAB2239, 1:1000, EMD Millipore), anti-histone 3 clone 06-755 (1:1000, Upstate), anti-LC3b (NB600-1384, 1:1000, Novus biologicals), anti-ubiquitin (MAB1510, 1:250, EMD Millipore), Phospho-4E-BP1 (Thr37/46) (Antibody #9459, 1:1000 Cell Signaling), p70 S6 Kinase Antibody (Antibody #9202, 1:1000 Cell Signaling), 4E-BP1 Antibody (Antibody #9452, 1:1000 Cell Signaling) and Phospho-p70 S6 Kinase (Thr389) (108D2, Antibody #9452, 1:1000 Cell Signaling). Vamp2 (aka synaptobrevin 2) (synaptic systems 104 211) Syntaxin 1 (polyclonal #I379, gift from the Thomas Sudhof lab to the CNCR FGA department), Synaptotagmin (polyclonal #W855, gift from the Thomas Sudhof lab to the CNCR FGA department), Munc18/STXBP1 (BD biosciences, Clone 31/Munc-18 (RUO), 610337)

Proteasomal levels and activity

After treatment of neurons with the indicated drugs for three days, neurons were incubated with Proteasome activity probe 1 (Berkers et al., 2007; de Jong et al., 2012) for two hours (250 nM in media). After labeling, cells were harvested and resuspended in HR buffer (50 mM Tris, 5 mM MgCl2, 250 mM sucrose, 1 mM DTT and 2 mM ATP, pH = 7.4). Cell lysis was achieved by sonication (Bioruptor Pico, Diagenode, high intensity for 5 minutes with an ON/OFF cycle of 30 s) at 4°C. After a centrifugation step (21.100 g for 15 minutes) to remove cell debris, protein concentration of the supernatant was determined by a NanoDrop spectrophotometer (Thermo Fisher Scientific) by measuring the absorbance at 280 nm. Equal amounts of protein were denatured by boiling in LDS (lithium dodecyl sulfate) sample buffer (Invitrogen Life Technologies, Carlsbad, CA, USA) containing 2.5% β-mercaptoethanol. Polypeptides were resolved by 12% SDS-PAGE using the NuPAGE system and MOPS running buffer (Invitrogen Life Technologies, Carlsbad, CA, USA). Wet gel slabs were imaged with a resolution of 50 μm, using the Typhoon FLA9800 imaging system (GE), with appropriate filter settings (λ(ex/em) = 480/530 nm). For Western Blot analysis, proteins were transferred onto PVDF membrane using the Trans-Blot Turbo Transfer System (Bio-Rad). Membranes were blocked with 5% BSA solution and subsequently incubated with primary antibody overnight at 4°C. After washing with PBS-Tween 20 (0.2%) membranes were incubated with secondary antibody and signals visualized by using an Amersham Imager 600 RGB (GE) imager or Odyssey Fc (Li-Cor). Western blot images within one panel showing a composition of bands of interest for a particular immunogen are always from the same image (identical blot and exposure), but cropped and grouped together for clarity. Stiches are indicated by vertical lines. Immunoblotting with an antibody against GADPH or actin was used to verify equal protein loading. Antibodies used were: anti-Actin (1:50,000; EMD Millipore), anti-PSMD7 (1:1000 PSMC2 (D5T1T) Rabbit mAb #14395), anti-PSMB5 (1:1,000; Enzo Lifesciences, AB_105409012), anti-GAPDH (1:1000; Life Technologies, AB_2536381). Secondary antibodies used (1:5000) were HRP-conjugated polyclonal swine anti-rabbit (DAKO Cat# P0217) and HRP-conjugated polyclonal rabbit anti-mouse (DAKO Cat# P0161).

Vectors and viral transduction

pCMV10-CYP46A1-flag vector was a kind gift from Dr. Rodrigues Elsa, University of Lisbon. CYP46A1-Flag was amplified by primers gtacaaaaaagcaggcttaaatggactacaaagaccatgacgg and GTACAAGAAAGCTGGGTAGCggatccTCAGCAGGGGGGTGGT and subcloned into the gateway entry vector pENTRrz1 and subsequently recombined into the destination vector pDESTlentiFGA1.0. Viral particles containing the CYP46A1 plasmid, or an GFP-only plasmid (pSyn(pr)EGFPLL3.7) were produced as described previously (Naldini et al., 1996). In short HEK293T cells were transfected with viral packing plasmids ENV plasmid p.MDG2:PACK plasmid pCMVΔR8.2 together with the transgene vector carrying GFP or CYP46A1. After 24 hours the medium is replaced, and after 48 hours the medium is harvested and concentrated using amicon filters with 100kDA cutoff (UFC910024, Millipore). The concentrated virus is then filtered through a 0.2 micron filter, aliquoted and frozen at −80 degrees. For use, virus is thawed directly prior to vial transduction. For experiments, three week differentiated NPC's (APPdp1-6) were replated on 96-well plates (2×10^5 living cells/well) and left to mature for another two weeks, after which they were infected with virus for 24 hours. After 24 hours, and again after 6 days media was replaced with fresh 200 μl NPC base + BDNF/GDNF/cAMP. 13 days after viral transduction the media was collected for 24-hydroxycholesterol measurements and cells were lysed for pThr231Tau/tTau analysis.

QUANTIFICATION AND STATISTICAL ANALYSIS

Statistical Analysis

The correlation coefficient (CC) for Figure 3E was calculated in excel using the formula

$$Correl(X,Y)=\frac{\sum(x-\overline{x})(y-\overline{y})}{\sqrt{\sum(x-\overline{x})^2\sum(y-\overline{y})^2}}$$

All other data was analyzed using GraphPad Prism Software (GraphPad Software). Statistical analysis comparing multiple groups was performed using a one-way Anova with a Tukey's multiple comparison test. Statistical analysis comparing two groups (treated versus untreated groups within the same genotype were calculated using multiple t tests. Data are depicted with bar graphs of the mean ± SEM of all values. Significance was defined as $p < 0.05$(*** $p < 0.001$, ** $p < 0.01$, *$p < 0.05$). n indicates independent measures from independent wells.

Cell Stem Cell, Volume *24*

Supplemental Information

Cholesterol Metabolism Is a Druggable Axis that Independently Regulates Tau and Amyloid-β in iPSC-Derived Alzheimer's Disease Neurons

Rik van der Kant, Vanessa F. Langness, Cheryl M. Herrera, Daniel A. Williams, Lauren K. Fong, Yves Leestemaker, Evelyne Steenvoorden, Kevin D. Rynearson, Jos F. Brouwers, J. Bernd Helms, Huib Ovaa, Martin Giera, Steven L. Wagner, Anne G. Bang, and Lawrence S.B. Goldstein

Supplemental Figure 1. Characterization of neuronal cultures and statin effects on pTau and viability, Related to Figure 1.

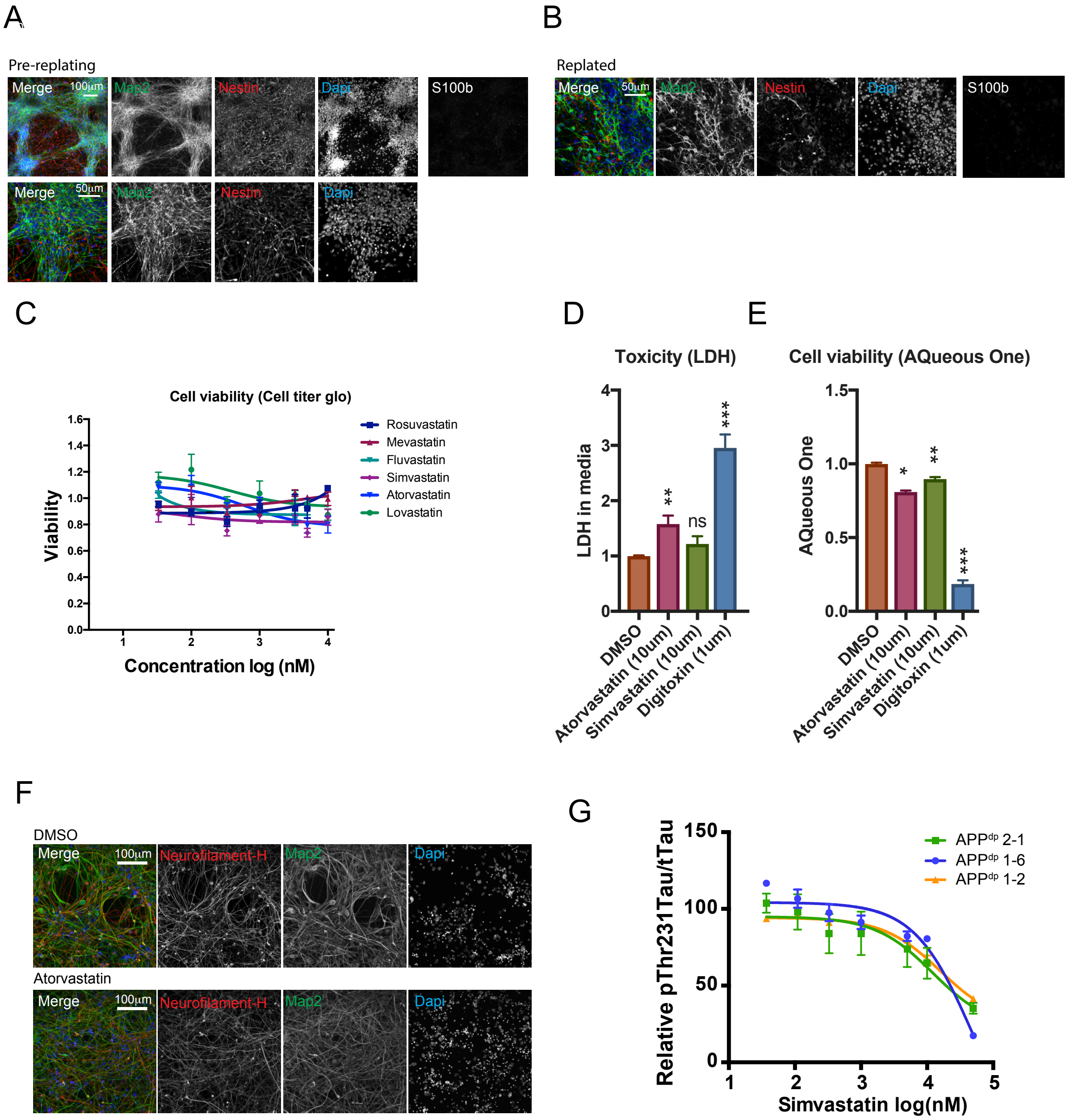

(**A-B**) Three week differentiated neuronal cultures (**A**) and differentiated neurons at point of screening (three weeks of differentiation, replating and two additional weeks of maturation) Scale bar represents 100µm. (**B**). Cultures were fixed and stained with antibodies for mature neurons (MAP2), neuronal progenitor cells (Nestin) and astrocytes (S100b). In keeping with previous observations at these time points the majority of cells are MAP2 neurons with a smaller group of nestin-positive neuronal precursor cells and absence/very low abundance of

astrocytic cells (Yuan et al., 2011). Scale bar represents 100μm. (**C**) Dosage effects of statins (five day treatment) on neuronal viability as measured by CeLLTiter-Glo luminescent cell viability assay. (**D-E**) Effects of a single dose of statins and a known toxic agent (Digitoxin) (five day treatment) on toxicity (**D**) determined by lactate dehydrogenase (LDH) released into the media (mean±SEM, n≥3) and (**E**) cell viability (mean±SEM, n≤3) determined by AQueous one cell viability assay (mean±SEM, n≥3). **(F)** Neuronal cultures (differentiated for three weeks, replated, matured for two more weeks) were treated with atorvastatin or DMSO for five days and stained for MAP2 and Neurofilament-H. Scale bar represents 100μm. **(G)** Dosage effects of simvastatin on pThr231Tau/tTau ratio in an additional FAD line from patient 1 (APP^{dp}1-2) and in an independent FAD APP^{dp} patient line (APP^{dp}2-1) (mean±SEM, n≥3). Dosage effects are compared to the effect of simvastatin on APP^{dp}1-6 from figure 1d. *** p<0.001, **p<0.01, *p<0.05.

Supplemental Figure 2. Effect of cholesterol-metabolism targeting compounds on neuronal viability and cholesterol metabolism, Related to Figure 2.

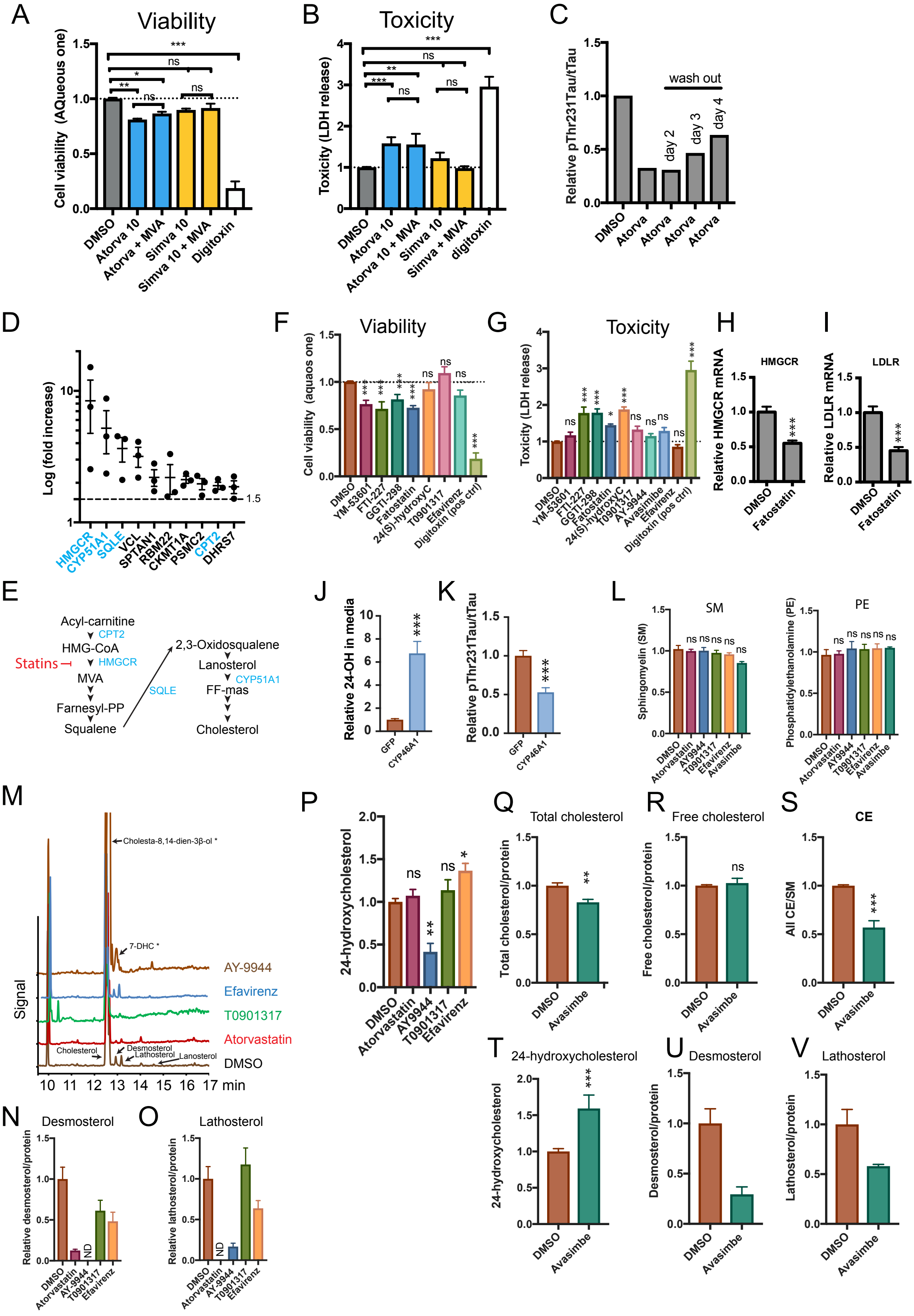

Effects of indicated compounds at same concentrations as in Figure 2 and a known toxic agent (Digitoxin) (five day treatment) on cell viability determined by (**A**) AQueous one cell viability assay (mean±SEM, n≥3). (**B**) lactate dehydrogenase (LDH) released into the media (mean±SEM, n≥3). (**C**) Effect of atorvastatin and atorvastatin washout on pThr231Tau/tTau ratio in APP^{dp}1-6. Neurons were treated for 5 days with DMSO or Atorvastatin and directly harvested, or after 5 days atorvastatin was washed-out and cells were harvested 2-4 days after wash out. (**D**) Purified neurons (NDC line B11 and B10) were treated for three days with atorvastatin or DMSO and harvested for quantitative mass spectrometry. In total three runs from three independent experiments were performed (two different experiments with B11 and one experiment with B10). All proteins that were increased >1.5 fold in all three runs were selected as hits and plotted by their effect on fold change (mean±sem). For complete dataset see Table S4. Proteins with a role in cholesterol synthesis are indicated in blue. HMGCR, 3-Hydroxy-3-Methylglutaryl-CoA Reductase; CYP51A1, Cytochrome P450 Family 51 Subfamily A Member 1 aka Lanosterol 14α-demethylase; SQLE, Squalene Epoxidase; VCL, Vinculin; SPTAN1, spectrin alpha, non-erythrocytic 1; RBM22, RNA Binding Motif Protein 22; CKMT1A, Creatine Kinase, Mitochondrial 1A; PSMC2, 26S proteasome regulatory subunit 7; CPT2, carnitine palmitoyltransferase 2; DHRS7, Dehydrogenase/Reductase 7. **(E)** Schematic depicting the identified hits in (D) with a role in cholesterol synthesis. **(F)** Cell viability (AQueous one) and **(G)** compound toxicity (LDH release) of conditions in Figure 2c. Digitoxin was used as a positive control for cell death (mean±SEM, n≥3). **(H-I)** APP^{dp}1-6 Neurons were treated for 5 days with 20µM fatostatin and mRNA levels of SREBP-target genes (3-hydroxy-3-methylglutaryl-coenzyme A reductase, HMGCR **(H)** and Low-Density Lipoprotein Receptor, LDLR **(I)**) were determined by quantitative real-time polymerase chain reaction (RT-qPCR) and normalized over housekeeping genes (RPLP27) (mean±SEM, n≥12). (**J-K**) Differentiated neurons were transduced with lentivirus carrying CYP46A1 or GFP expression vectors. After

13 days 24-hydroxycholesterol in media (**J**) and neuronal pThr231Tau/tTau ratio was determined (**K**). (**L**) APP^{dp}1-6 neurons were treated with Atorvastatin (10 μM), AY-9944 (10 μM), T0901317 (10μM), Efavirenz (10μM) and Avasimibe (10μM) and lipid analysis was performed to measure the proportion of sphingomyelin (SM) and Phosphatidylethanolamine (PE) (mean±SEM, n≥3). (**M-O**) APP^{dp}1-6 neurons were treated with Atorvastatin (10 μM), AY-9944 (5 μM), T0901317 (10μM) and Efavirenz (10μM) for three days and lipid analysis was performed to measure cholesterol precursors. **(M)** Typical traces from lipid GM-MS showing detected cholesterol precursors in neuronal cultures treated with indicated inhibitors (zoomed-in to show precursor peaks). **(N-O**) Overall precursor levels were low abundant and could be quantified only for desmosterol (**N**) and lathosterol (**O**) (mean±SEM). Efavirenz had previously been shown to increase precursor levels, but in our cultures did not increase (rather modestly decrease) precursor levels. *In the AY-9944 treated neurons 7DHC and Cholesta-8,14-dien-3β-o accumulated as previously reported (Giera et al., 2007). N≥4 for all treatments, expect for desmosterol levels in atorvastatin treated samples, which were below detection in two out of four samples. ND=not detected. (**P**) 24-hydroxycholesterol (five day treatment) levels in media from APP^{dp}1-6 neuronal cultures treated with indicated compounds (mean±SEM, n≥3). (**Q-V**) APP^{dp}1-6 neurons were treated with avasimibe (5μM) and lipid analysis was performed to measure (**Q**) total cholesterol (mean±sem, N≥8), three day treatment, (**R**) free cholesterol (mean±SEM, n≥4), three day treatment (**S**) CE (mean±SEM, n≥8) three day treatment (**T**) 24-hydroxycholesterol in media (mean±SEM, n≥4) (five day treatment) and (**U**) desmosterol and (**V**) lathosterol both (mean±SEM, n≥3) three day treatment. *** $p<0.001$, **$p<0.01$, *$p<0.05$.

Supplemental Figure 3. The effect of CE-reducing drugs on pTau in neurons with different genetic backgrounds, Related to Figure 3.

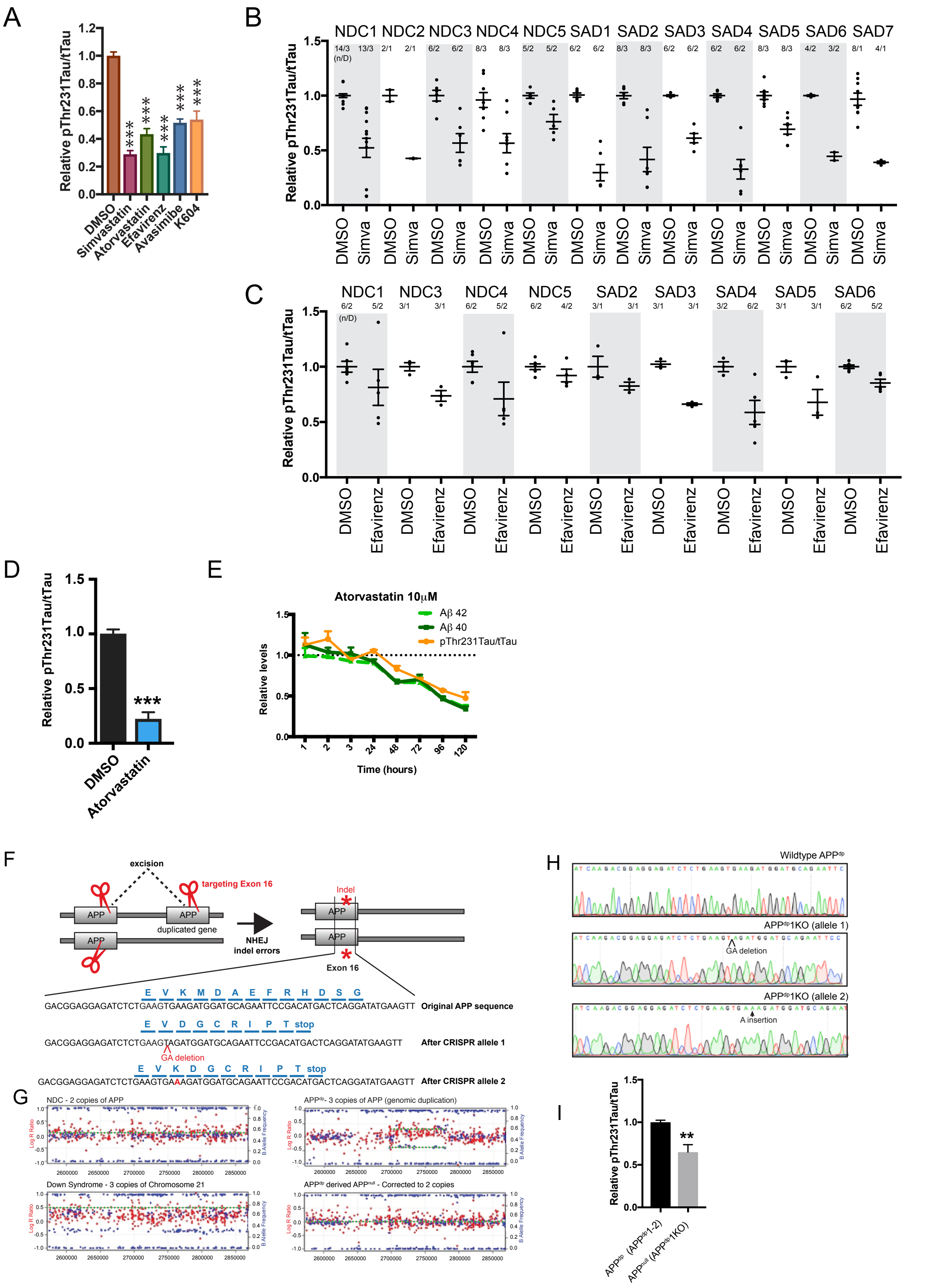

(A) NDC CV4a neurons were treated with indicated compounds; Simvastatin (10 μM), Atorvastatin (10 μM), Efavirenz (25μM), Avasimibe (10μM) and K604 (50μM) pThr231Tau/tTau levels were determined by ELISA (mean±SEM, n≥3). **(B)** Effect of simvastatin (10μm) (**B**) or Efavirenz (10μm) (**C**) treatment on pThr231Tau/tTau in neuronal lines from indicated NDC and SAD subject iPSC-derived neurons (mean±SEM). Same as in main figure 3b-c but here plotted for individual lines. Indicated (n/D) are number of measurements (n) and number of independent differentiations (D). **(D)** Effect of atorvastatin (10μm) treatment on *in vitro* cultured mouse cortical neurons (mean±SEM, n≥3)**. (E**) APP^{dp} 1-6 neurons were treated for indicated time points with atorvastatin. Cell lysates and media were analyzed by ELISA for pThr231Tau/ tTau and Aβ levels. (**F**) Schematic overview of the gene-editing strategy to generate an isogenic APP^{null} line in APP^{dp}-genetic background. The original patient has a duplication of APP (three copies of the gene for APP). Cutting by CRISPR/Cas9 at indicated sites (red scissors) causes the excision of the duplication site between cut-sites and correction of copy number. Non-homologues end-joining (NHEJ) insertion/deletion errors (indel) generated during the repair introduce respective premature stop-codons in the two remaining alleles as indicated. (**G**) Copy number analysis (digital karyotyping) of the region with APP duplication for a non-demented control genome, Down syndrome genome (duplication of the entire chromosome 21) and APP^{dp} line before (3 copies of APP) and after ("corrected to 2 copies of APP") gene editing showing copy number correction. **(H)** Sequencing results from TOPO-cloning of the individual alleles verifying a "GA" deletion on one allele and an "A" insertion in the other allele, compared to wildtype sequence (APPdp) (upper box) generating a premature stop. (**I**) pThr231Tau/tTau ratio in isogenic purified APP^{dp} (APP^{dp}1-2) and APP^{null} (APPdp1KO) neurons (five days after sort) (mean±SEM, n≥3). *** p<0.001, **p<0.01, *p<0.05.

Supplemental Figure 4. Characterization of APP-Δcholesterol neurons, Related to Figure 4.

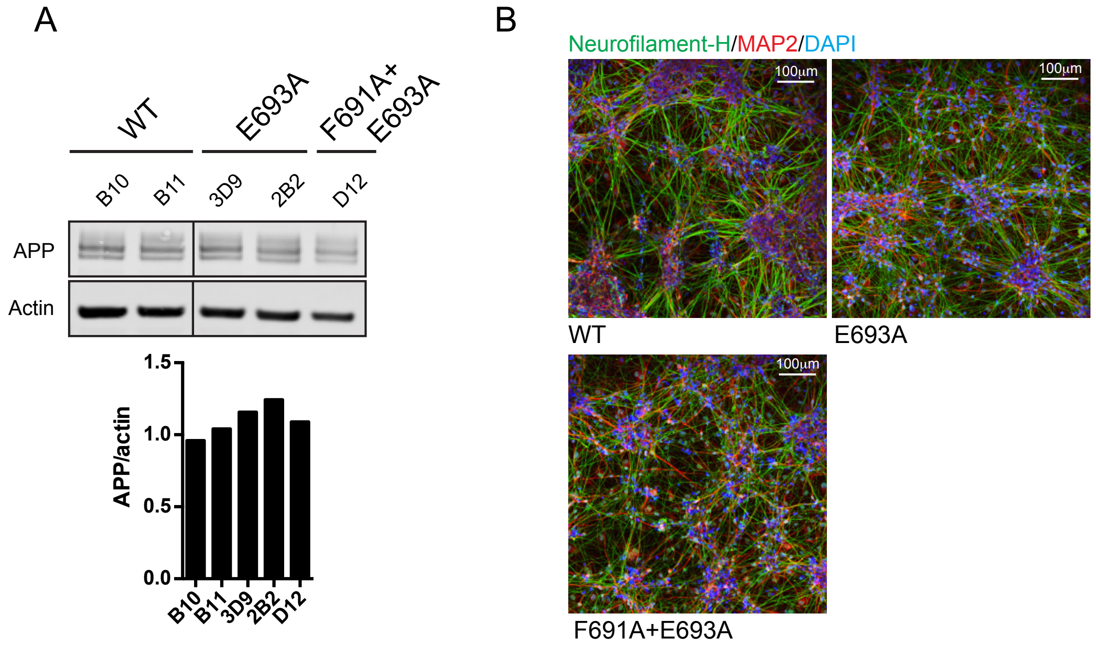

(**A**) Analysis of APP-protein level in control and mutant lines, showing normal expression of full-length APP. Image is a composite of different loading positions on same blot, stich is indicated by vertical line. (**B**) Immunofluorescence staining of wild type and indicated APP-Δcholesterol neurons showing neuronal morphology and polarization. Scale bar represents 100μm.

Supplemental Figure 5. Characterization of drug-treatment effects on neuronal cultures and pTau-mediated turnover by the proteasome, Related to Figure 5.

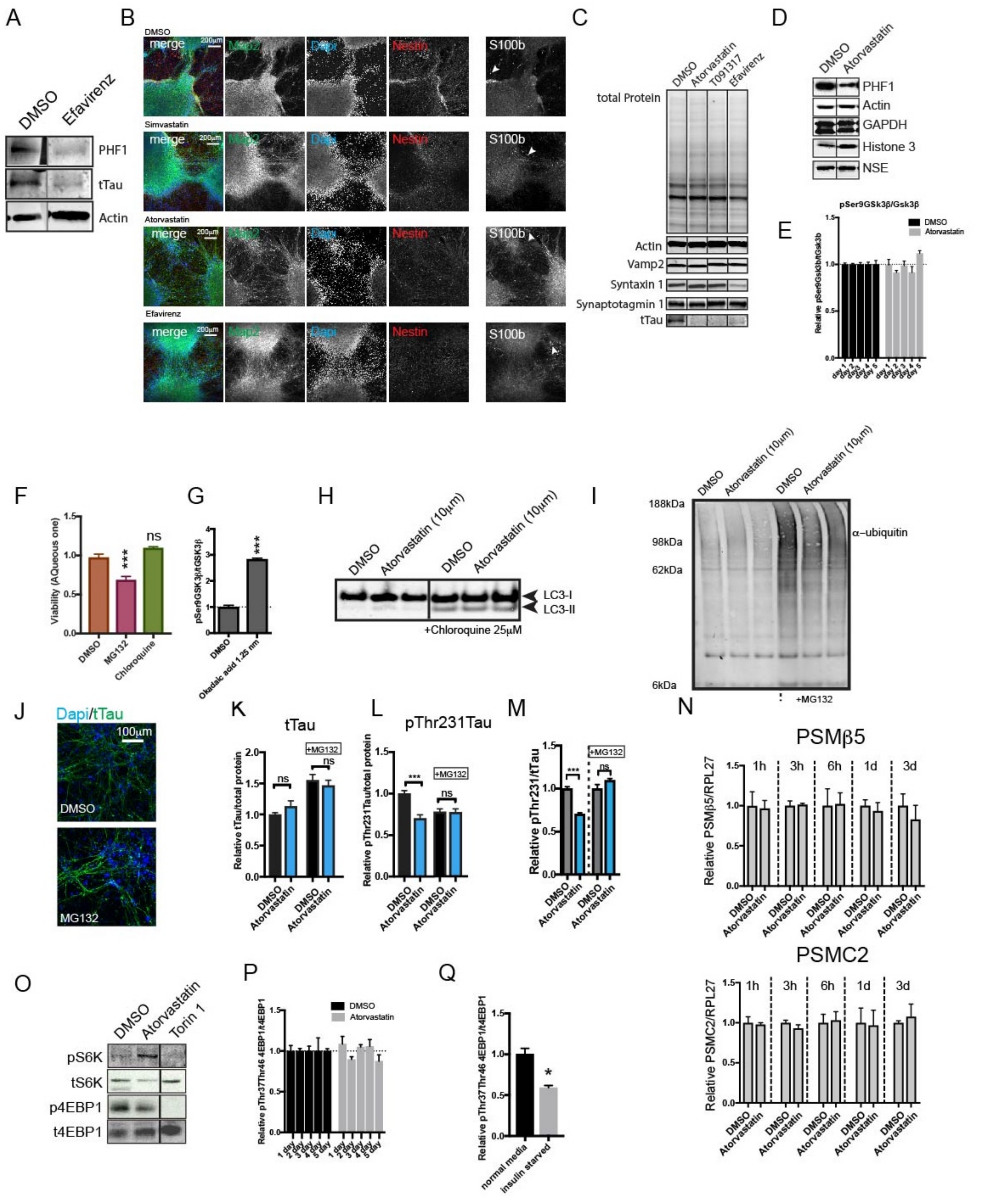

(**A**) The effect of Efavirenz (five day treatment, 10µM, APP^{dp}1-6 neurons) on Tau levels and Tau phosphorylation as assessed by western blot (see quantification in main figure 5B). Image

is a composite of different loading positions on same blot, stich is indicated by vertical line. (**B**) Neuronal (MAP2), precursor (Nestin) and astrocyte (S100b) staining of three-week differentiated non-replated neurons (conditions used for western blot) after five day treatment with DMSO, Simvastatin (10µm), Atorvastatin (10µm) or Efavirenz (25µm) in CV4a neurons. High non-specific background staining for S100b was observed in this line, arrows indicate examples of low abundant above-background S100b positive cells. Scale bar represents 200µm. (**C**) Western blot analysis of indicated synaptic proteins after treatment with DMSO, Atorvastatin (10µm), T0901317 (10µm) Efavirenz (25µm) (five day treatment, APP^{dp}1-6 neurons). Image is a composite of different loading positions on same blot, stiches are indicated by vertical lines. (**D**) Western blot analysis of housekeeping genes and neuron-specific enolase (NSE) after treatment with DMSO or Atorvastatin (10µm) (five day treatment, APP^{dp}1-6 neurons). Image is a composite of different loading positions on same blot, stiches are indicated by vertical lines. (**E**) APP^{dp}1-6 neurons were treated for indicated time points with atorvastatin. Cell lysates were analyzed by ELISA for pSer9GSK3b/ tGsk3b (mean±SEM, n≥3). **(F)** APP^{dp}1-6 neurons were treated for 3 days with 25µM chloroquine (inhibitor of lysosomal acidification and autophagosomal degradation) or 5µM MG-132 (proteasomal inhibitor) and cell viability was determined (mean±sem, N≥3). **(G)** APP^{dp}1-6 neurons were treated for 3 days with 1.25nM okadaic acid (phosphatase inhibitor) and pGSK3b/GSK3b levels were determined by ELISA as a measure of phosphatase inhibition (mean±sem, N≥3). **(H-I)** APP^{dp}1-6 neurons were treated for 3 days with 10µM atorvastatin in the presence of 25µM chloroquine (**H**) or 5µM MG-132 (**I**). Lysates were collected and assessed by western blot staining with antibodies against (**H**) microtubule-associated protein 1A/1B-light chain 3 (LC3) to show inhibition of autophagic flux by chloroquine and staining with antibodies for (**I**) ubiquitin to show accumulation of ubiquitinated proteins in MG-132 treated samples. (**J**) APP^{dp}1-6 neurons were treated for 3 days with 5µM MG-132, fixed and stained with a total Tau antibody. (**K**) tTau/total protein levels

and **(L)** pThr231Tau/total protein in APP^{dp} neurons co-treated with atorvastatin (10µm) and the proteasome inhibitor (MG132, 5µM) for 3 days as measured by ELISA. Results are averages from two independent APP^{dp} lines (APP^{dp} 1-6 and 2-1) (mean±SEM, n≥3 per line). (**M**) pThr231Tau/tTau levels in WT neurons co-treated with atorvastatin (10µm) and a proteasome inhibitor (MG132, 5µM) for 3 days as measured by ELISA. Results are averages from two independent WT lines (CV4a and line 151) (mean±sem, N≥3 per line). MG-132 is unstable in solution and was therefore added fresh every day in all experiments. (**N**) APP^{dp}1-6 Neurons were treated for indicated time points with 10µM atorvastatin and mRNA levels of proteasomal subunits PSMβ5 and PSMC2 were determined by quantitative real-time polymerase chain reaction (RT-qPCR) and normalized over housekeeping genes (RPLP27) (mean±SEM, n≥3). (**O**) APP^{dp}1-6 neurons were treated for 5 days with DMSO, 10µM atorvastatin or Torin1 (an mTor inhibitor) and phosphorylation status of mTor target proteins was determined by western blot. Image is a composite of different loading positions on same blot, stiches are indicated by vertical lines. (**P**) APP^{dp}1-6 neurons were treated for indicated time points with DMSO or 10µM atorvastatin and phosphorylation status of the mTor target 4EBP1 was determined by ELISA (mean±SEM, n≥3) (**Q**) Positive control for 4EBP ELISA. APP^{dp}1-6 neurons were treated for three days with insulin containing media or insulin-free media and phosphorylation status of the mTor target 4EBP1 was determined by ELISA (mean±SEM, n≥3). *** p<0.001, **p<0.01, *p<0.05.

Supplemental Figure 6. Astrocytic and neuronal viability in response to statins and efavirenz, Related to Figure 6.

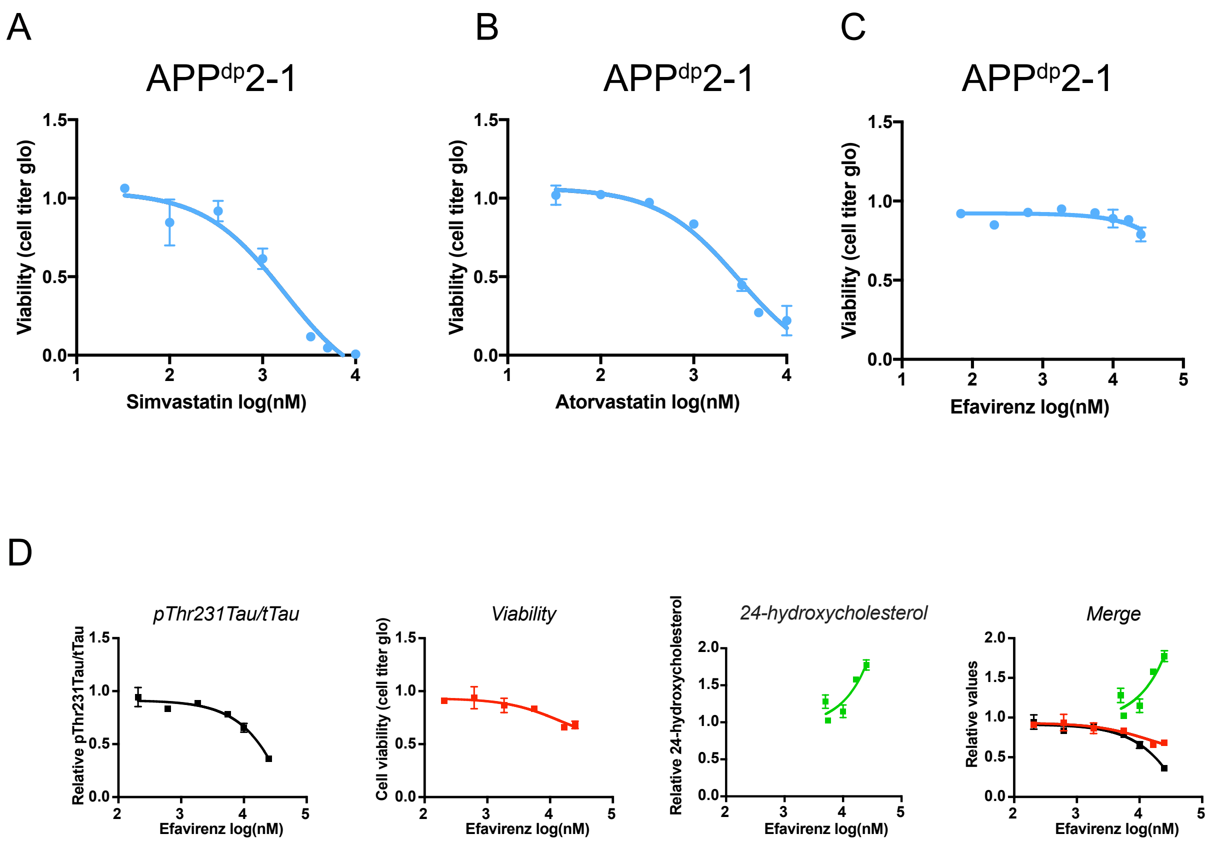

iPSC-derived APP^{dp}2-1astrocytes were treated for three days with increasing concentrations of **(A)** simvastatin, **(B)** atorvastatin and **(C)** efavirenz and viability was determined (cell titer glo). **(D)** APP^{null} (APPdp1KO) neurons were treated for five days with increasing concentrations efavirenz and pThr231Tau/Tau levels, 24-hydroxycholesterol levels and viability was measured (mean±SEM, n≥3).

Supplemental Table 2. Coefficient of variation for screening campaign, Related to Figure 1.

	ptau/total tau ratio			Cell Titer Glo		
plate #	ave	std dev	cv	ave	std dev	cv
1	4.89	1.05	0.21	427566	87006	0.20
2	4.38	0.76	0.17	439798	105410	0.24
3	4.80	0.68	0.14	417468	46284	0.11
4	4.42	0.71	0.16	413676	65711	0.16
5	6.05	0.72	0.12	540691	134415	0.25
6	6.88	0.60	0.09	538648	128503	0.24
7	5.87	1.64	0.28	252201	42936	0.17
8	5.79	1.69	0.29	237674	55155	0.23
9	5.03	1.12	0.22	193892	56904	0.29
10	5.30	1.14	0.21	276671	43889	0.16
11	4.36	1.01	0.23	202913	60936	0.30
12	5.18	1.15	0.22	215490	47718	0.22
13	5.15	1.23	0.24	221407	75762	0.34
14	4.30	0.65	0.15	224197	41101	0.18
15	6.07	1.47	0.24	233372	57994	0.25
16	7.76	0.69	0.09	534782	49171	0.09
17	6.57	1.30	0.20	377836	67072	0.18
18	4.38	1.04	0.24	333269	51268	0.15
19	4.99	1.35	0.27	336305	76970	0.23
20	5.09	0.94	0.19	421618	90686	0.22
			AVERAGE CV			AVERAGECV
			**0.20**			**0.21**

Coefficient of variation (CV) for pThr231Tau/tTau and cell titer glo in DMSO-treated samples for each of the 20 384 well plates used for the screening campaign (primary screen, conformation, dose response experiments)

Supplemental Table 5. Absolute levels of cholesterol, Related to Figure 2.

	free cholesterol ng/ug protein		total cholesterol ng/ug protein	
	mean	stdev	mean	stdev
DMSO	85.9	1.6	119.1	10.1
Atorvastatin	89.5	6.3	99.4	8.3
AY-9944	67.2	2.5	76.6	4.5
T0901317	73.0	6.9	104.2	8.2
Efavirenz	75.1	6.6	88.1	4.5
Avasimibe	88.2	8.4	98.7	9.8

Table depicts the absolute levels of free cholesterol (N=8) and total cholesterol (N=4) over protein corresponding to the relative levels plotted in figure 2d and e.

Supplemental Table 6. Oligonucleotides used in this study, Related to STAR methods.

Oligonucleotides	Source	Identifier
5’tattgcatttagaaattaaaattctttttcttaatttgttttcaaggtgttcttt gcagccgatgtgggttcaaacaaaggtgcaatcattggactcatggtgg gcggtgttgtcatagc3’	This Paper	E693A Repair Oligo
5’ttatattgcatttagaaattaaaattctttttcttaatttgttttcaaggtgttc gctgcagccgatgtgggttcaaacaaaggtgcaatcattggactcatgg tgggcggtgttgtcat3’	This Paper	E693A+F691A Repair Oligo
5’CTTCCTCGAACTGGGGAAGC3’	This Paper	APP Chol mut region forward primer
5’TCACGGTAAGTTGCAATGAATGA3’	This Paper	APP Chol mut region reverse primer
5’CCAACCAGTTGGGCAGAGAA3’	This Paper	APP Chol mut genomic DNA Sequencing Primer
5’CGTGGAATGGCAATTTTAGGTCC3’	This Paper	HMGCR-F (qPCR)
HMGCR-R (qPCR): 5’ATTTCAAGCTGACGTACCCCT3’	This Paper	*HMGCR-R (qPCR)*
LDLR-F (qPCR): 5’GTCTTGGCACTGGAACTCGT3’	This Paper	*LDLR-F (qPCR)*
LDLR-R (qPCR): 5’CTGGAAATTGCGCTGGAC3’	This Paper	LDLR-R (qPCR)
5’GCTACCGGTGAACCAGCG3’	This Paper	PSMB5 FW (qPCR)
5’CAACTATGACTCCATGGCGGA3’	This Paper	PSMB5 RV (qPCR)
5’TGAGAGTGGGCGTGGATAGA3’	This Paper	PSMC2 FW (qPCR)
5’GTACCGGGTGGACCAAAGAG3’	This Paper	PSMC2 RV (qPCR)
5’AAACCGCAGTTTCTGGAAGA3’	This Paper	RPL27-F (qPCR)
5’TGGATATCCCCTTGGACAAA3’	This Paper	RPL27-R (qPCR)
5’gtacaaaaaagcaggcttaaatggactacaaagaccatgacgg3’	This paper	CYP46A1 PCR primer 1
5’GTACAAGAAAGCTGGGTAGCggatccTCAGC AGGGGGGTGGT3’	This paper	CYP46A1 PCR primer 2
